# Global, regional, and national burden of household air pollution, 1990–2021: a systematic analysis for the Global Burden of Disease Study 2021

**DOI:** 10.1016/S0140-6736(24)02840-X

**Published:** 2025-04-05

**Authors:** GBD 2021 HAP Collaborators, Fiona B Bennitt, Sarah Wozniak, Kate Causey, Sandra Spearman, Chukwuma Okereke, Vanessa Garcia, Nadim Hashmeh, Charlie Ashbaugh, Atef Abdelkader, Meriem Abdoun, Muhammed Jemal Abdurebi, Armita Abedi, Roberto Ariel Abeldaño Zuñiga, Richard Gyan Aboagye, Bilyaminu Abubakar, Ahmed Abu-Zaid, Mesafint Molla Adane, Oyelola A Adegboye, Victor Adekanmbi, Abiola Victor Adepoju, Temitayo Esther Adeyeoluwa, Olorunsola Israel Adeyomoye, Rishan Adha, Muhammad Sohail Afzal, Saira Afzal, Feleke Doyore Agide, Aqeel Ahmad, Danish Ahmad, Muayyad M Ahmad, Sajjad Ahmad, Ali Ahmadi, Sepideh Ahmadi, Anisuddin Ahmed, Ayman Ahmed, Haroon Ahmed, Marjan Ajami, Rufus Olusola Akinyemi, Salah Al Awaidy, Hanadi Al Hamad, Muaaz M Alajlani, Mulubirhan Assefa Alemayohu, Adel Ali Saeed Al-Gheethi, Abid Ali, Waad Ali, Sheikh Mohammad Alif, Sami Almustanyir, Nelson Alvis-Guzman, Nelson J Alvis-Zakzuk, Hany Aly, Hubert Amu, Ganiyu Adeniyi Amusa, Tadele Fentabel Anagaw, Boluwatife Stephen Anuoluwa, Iyadunni Adesola Anuoluwa, Saeid Anvari, Ekenedilichukwu Emmanuel Anyabolo, Geminn Louis Carace Apostol, Aleksandr Y Aravkin, Demelash Areda, Brhane Berhe Aregawi, Olatunde Aremu, Akeza Awealom Asgedom, Mubarek Yesse Ashemo, Tahira Ashraf, Seyyed Shamsadin Athari, Sina Azadnajafabad, Ahmed Y Azzam, Giridhara Rathnaiah Babu, Saeed Bahramian, Kiran Bam, Maciej Banach, Biswajit Banik, Mehmet Firat Baran, Francesco Barone-Adesi, Sandra Barteit, Hameed Akande Bashiru, Pritish Baskaran, Mohammad-Mahdi Bastan, Sanjay Basu, Saurav Basu, Sefealem Assefa Belay, Melesse Belayneh, Apostolos Beloukas, Derrick A Bennett, Devidas S Bhagat, Dinesh Bhandari, Pankaj Bhardwaj, Sonu Bhaskar, Ajay Nagesh Bhat, Priyadarshini Bhattacharjee, Gurjit Kaur Bhatti, Manpreet S Singh Bhatti, Cem Bilgin, Mary Sefa Boampong, Sri Harsha Boppana, Samuel Adolf Bosoka, Sofiane Boudalia, Fan Cao, Rama Mohan Chandika, Gashaw Sisay Chanie, Vijay Kumar Chattu, Anis Ahmad Chaudhary, Akhilanand Chaurasia, Guangjin Chen, Yifan Chen, Ritesh Chimoriya, Bryan Chong, Devasahayam J Christopher, Isaac Sunday Chukwu, Aaron J Cohen, Natalia Cruz-Martins, Omid Dadras, Xiaochen Dai, Patience Unekwuojo Daikwo, Samuel Demissie Darcho, Saswati Das, Juana Maria Delgado-Saborit, Belay Desye, Sagnik Dey, Meghnath Dhimal, Daniel Diaz, Thanh Chi Do, Ojas Prakashbhai Doshi, Abdel Rahman E'mar, Alireza Ebrahimi, Hisham Atan Edinur, Aziz Eftekharimehrabad, Temitope Cyrus Ekundayo, Ibrahim Farahat El Bayoumy, Syed Emdadul Haque, Theophilus I Emeto, Habtamu Demelash Enyew, Ayesha Fahim, Adekunle Gregory Fakunle, Sasan Faridi, Timur Fazylov, Alireza Feizkhah, Florian Fischer, Morenike Oluwatoyin Folayan, Sridevi G, Muktar A Gadanya, Xiang Gao, Miglas Welay Gebregergis, Mesfin Gebrehiwot, Teferi Gebru Gebremeskel, Afsaneh Ghasemzadeh, Nermin Ghith, Mahaveer Golechha, Davide Golinelli, Shi-Yang Guan, Zhifeng Guo, Bhawna Gupta, Lalit Gupta, Rabih Halwani, Ahmed I Hasaballah, Md Saquib Hasnain, Simon I Hay, Demisu Zenbaba Heyi, Kamal Hezam, Nguyen Quoc Hoan, Ramesh Holla, Hassan Hosseinzadeh, Chengxi Hu, Hong-Han Huynh, Bing-Fang Hwang, Segun Emmanuel Ibitoye, Oluwatope Olaniyi Idowu, Adalia Ikiroma, Mustapha Immurana, Arit Inok, Muhammad Iqhrammullah, Rakibul M Islam, Sheikh Mohammed Shariful Islam, Vinothini J, Ammar Abdulrahman Jairoun, Abhishek Jaiswal, Mihajlo Jakovljevic, Reza Jalilzadeh Yengejeh, Manthan Dilipkumar Janodia, Shubha Jayaram, Alelign Tasew Jema, Ravi Prakash Jha, Jost B Jonas, Nitin Joseph, Vidya Kadashetti, Kehinde Kazeem Kanmodi, Sushil Kumar Kansal, Ibraheem M Karaye, Gbenga A Kayode, Himanshu Khajuria, Amirmohammad Khalaji, Vishnu Khanal, Khaled Khatab, Khalid A Kheirallah, Atulya Aman Khosla, Majid Khosravi, Shivakumar KM, Luke D Knibbs, Gerbrand Koren, Parvaiz A Koul, Kewal Krishan, Barthelemy Kuate Defo, Mohammed Kuddus, Mukhtar Kulimbet, Vishnutheertha Kulkarni, Ashish Kumar, Dewesh Kumar, Nithin Kumar, Om P Kurmi, Chandrakant Lahariya, Hanpeng Lai, Tuo Lan, Paolo Lauriola, Nhi Huu Hanh Le, Munjae Lee, Seung Won Lee, Stephen S Lim, Gang Liu, Shuke Liu, Wei Liu, José Francisco López-Gil, Jay B Lusk, Sandeep B Maharaj, Kashish Malhotra, Ahmad Azam Malik, Iram Malik, Lesibana Anthony Malinga, Alexander G Mathioudakis, Rita Mattiello, Andrea Maugeri, Tesfahun Mekene Meto, Hadush Negash Meles, Ritesh G Menezes, Sultan Ayoub Meo, Seid Tiku Mereta, Tuomo J Meretoja, Tomislav Mestrovic, Laurette Mhlanga, Ted R Miller, Andreea Mirica, Erkin M Mirrakhimov, Moonis Mirza, Awoke Misganaw, Prasanna Mithra, Jama Mohamed, Nouh Saad Mohamed, Abdollah Mohammadian-Hafshejani, Mustapha Mohammed, Shafiu Mohammed, Ali H Mokdad, Shaher Momani, Himel Mondal, Lidia Morawska, Rohith Motappa, Sumaira Mubarik, Kavita Munjal, Yanjinlkham Munkhsaikhan, Christopher J L Murray, Woojae Myung, Sanjeev Nair, Vinay Nangia, Muhammad Naveed, Nawsherwan, Rawlance Ndejjo, Dang Nguyen, Hien Quang Nguyen, Van Thanh Nguyen, Taxiarchis Konstantinos Nikolouzakis, Vikram Niranjan, Efaq Ali Noman, Syed Toukir Ahmed Noor, Abbas Norouzian Baghani, Jean Jacques Noubiap, Ogochukwu Janet Nzoputam, Bogdan Oancea, Ismail A Odetokun, Daniel Bogale Odo, Akinyemi O D Ofakunrin, Onome Bright Oghenetega, Osaretin Christabel Okonji, Andrew T Olagunju, Tosin Abiola Olasehinde, Isaac Iyinoluwa Olufadewa, Gideon Olamilekan Oluwatunase, Ahmed Omar Bali, Mohammad Mehdi Ommati, Abiodun Olusola Omotayo, Maureene Auma Ondayo, Adrian Otoiu, Mayowa O Owolabi, Mahesh Padukudru P A, Jagadish Rao Padubidri, Ioannis Pantazopoulos, Shahina Pardhan, Pragyan Paramita Parija, Romil R Parikh, Eun-Kee Park, Ashwaghosha Parthasarathi, Jay Patel, Siddhartha Pati, Shrikant Pawar, Prince Peprah, Gavin Pereira, Arokiasamy Perianayagam, Hoang Tran Pham, Ramesh Poluru, Akram Pourshams, Jalandhar Pradhan, Elton Junio Sady Prates, Dimas Ria Angga Pribadi, Jagadeesh Puvvula, Ata Rafiee, Pankaja Raghav, Fakher Rahim, Mohammad Hifz Ur Rahman, Mosiur Rahman, Muhammad Aziz Rahman, Amir Masoud Rahmani, Mohammad Rahmanian, Sathish Rajaa, Rayan Rajabi, Prashant Rajput, Mahmoud Mohammed Ramadan, Juwel Rana, Kritika Rana, Chhabi Lal Ranabhat, Drona Prakash Rasali, Santosh Kumar Rauniyar, Salman Rawaf, Elrashdy M Moustafa Mohamed Redwan, Nazila Rezaei, Jefferson Antonio Buendia Rodriguez, Susanne Röhr, Gholamreza Roshandel, Himanshu Sekhar Rout, Priyanka Roy, Michele Russo, Cameron John Sabet, Basema Ahmad Saddik, Umar Saeed, Narjes Saheb Sharif-Askari, Amirhossein Sahebkar, Pragyan Monalisa Sahoo, Afeez Abolarinwa Salami, Dauda Salihu, Abdallah M Samy, Milena M Santric-Milicevic, Tanmay Sarkar, Maheswar Satpathy, Ganesh Kumar Saya, Md Abu Sayeed, Austin E Schumacher, Mihretu Tagesse Sergindo, Yashendra Sethi, Allen Seylani, Samiah Shahid, Sunder Sham, Muhammad Aaqib Shamim, Anas Shamsi, Aziz Sheikh, Pavanchand H Shetty, Aminu Shittu, Ivy Shiue, Emmanuel Edwar Siddig, Paramdeep Singh, Surjit Singh, Md Shahjahan Siraj, Jeffrey D Stanaway, Leo Stockfelt, Kurt Straif, Chandan Kumar Swain, Berwin Singh Swami Vetha, Seyyed Mohammad Tabatabaei, Mircea Tampa, Haosu Tang, Manoj Tanwar, Elvis Enowbeyang Tarkang, Yibekal Manaye Tefera, Mohamad-Hani Temsah, Reem Mohamad Hani Temsah, Ramna Thakur, Friedrich Thienemann, Nigusie Selomon Tibebu, Krishna Tiwari, Marcos Roberto Tovani-Palone, Jaya Prasad Tripathy, Aristidis Tsatsakis, Munkhtuya Tumurkhuu, Aniefiok John Udoakang, Sana Ullah, Sanaz Vahdati, Siavash Vaziri, Madhur Verma, Simone Vidale, Simona Villani, Karn Vohra, Theo Vos, Gizachew Tadesse Wassie, Haftom Legese Weldetinsaa, Adhena Ayaliew Werkneh, Nuwan Darshana Wickramasinghe, Marcin W Wojewodzic, Tewodros Eshete Wonde, Felicia Wu, Zenghong Wu, Hong Xiao, Suowen Xu, Mukesh Kumar Yadav, Saba Yahoo Syed, Sanni Yaya, Arzu Yiğit, Vahit Yiğit, Dehui Yin, Dong Keon Yon, Naohiro Yonemoto, Chuanhua Yu, Leila Zaki, Mohammed G M Zeariya, Youjie Zeng, Chunxia Zhai, Haijun Zhang, Zhiqiang Zhang, Bin Zhu, Sa'ed H Zyoud, Samer H Zyoud, Michael Brauer, Katrin Burkart

## Abstract

**Background:**

Despite a substantial reduction in the use of solid fuels for cooking worldwide, exposure to household air pollution (HAP) remains a leading global risk factor, contributing considerably to the burden of disease. We present a comprehensive analysis of spatial patterns and temporal trends in exposure and attributable disease from 1990 to 2021, featuring substantial methodological updates compared with previous iterations of the Global Burden of Diseases, Injuries, and Risk Factors Study, including improved exposure estimations accounting for specific fuel types.

**Methods:**

We estimated HAP exposure and trends and attributable burden for cataract, chronic obstructive pulmonary disease, ischaemic heart disease, lower respiratory infections, tracheal cancer, bronchus cancer, lung cancer, stroke, type 2 diabetes, and causes mediated via adverse reproductive outcomes for 204 countries and territories from 1990 to 2021. We first estimated the mean fuel type-specific concentrations (in μg/m^3^) of fine particulate matter (PM_2·5_) pollution to which individuals using solid fuels for cooking were exposed, categorised by fuel type, location, year, age, and sex. Using a systematic review of the epidemiological literature and a newly developed meta-regression tool (meta-regression: Bayesian, regularised, trimmed), we derived disease-specific, non-parametric exposure–response curves to estimate relative risk as a function of PM_2·5_ concentration. We combined our exposure estimates and relative risks to estimate population attributable fractions and attributable burden for each cause by sex, age, location, and year.

**Findings:**

In 2021, 2·67 billion (95% uncertainty interval [UI] 2·63–2·71) people, 33·8% (95% UI 33·2–34·3) of the global population, were exposed to HAP from all sources at a mean concentration of 84·2 μg/m^3^. Although these figures show a notable reduction in the percentage of the global population exposed in 1990 (56·7%, 56·4–57·1), in absolute terms, there has been only a decline of 0·35 billion (10%) from the 3·02 billion people exposed to HAP in 1990. In 2021, 111 million (95% UI 75·1–164) global disability-adjusted life-years (DALYs) were attributable to HAP, accounting for 3·9% (95% UI 2·6–5·7) of all DALYs. The rate of global, HAP-attributable DALYs in 2021 was 1500·3 (95% UI 1028·4–2195·6) age-standardised DALYs per 100 000 population, a decline of 63·8% since 1990, when HAP-attributable DALYs comprised 4147·7 (3101·4–5104·6) age-standardised DALYs per 100 000 population. HAP-attributable burden remained highest in sub-Saharan Africa and south Asia, with 4044·1 (3103·4–5219·7) and 3213·5 (2165·4–4409·4) age-standardised DALYs per 100 000 population, respectively. The rate of HAP-attributable DALYs was higher for males (1530·5, 1023·4–2263·6) than for females (1318·5, 866·1–1977·2). Approximately one-third of the HAP-attributable burden (518·1, 410·1–641·7) was mediated via short gestation and low birthweight. Decomposition of trends and drivers behind changes in the HAP-attributable burden highlighted that declines in exposures were counteracted by population growth in most regions of the world, especially sub-Saharan Africa.

**Interpretation:**

Although the burden attributable to HAP has decreased considerably, HAP remains a substantial risk factor, especially in sub-Saharan Africa and south Asia. Our comprehensive estimates of HAP exposure and attributable burden offer a robust and reliable resource for health policy makers and practitioners to precisely target and tailor health interventions. Given the persistent and substantial impact of HAP in many regions and countries, it is imperative to accelerate efforts to transition under-resourced communities to cleaner household energy sources. Such initiatives are crucial for mitigating health risks and promoting sustainable development, ultimately improving the quality of life and health outcomes for millions of people.

**Funding:**

Bill & Melinda Gates Foundation.

## Introduction

Household air pollution (HAP) from solid cooking fuels is a known source of health-relevant exposure for about 3 billion people worldwide.^1^, ^2^, ^3^ People living in households using primarily solid fuels (coal or charcoal, wood, crop residues, and dung) for cooking are exposed to high levels of HAP from particulate matter with a diameter of less than 2·5 μm (PM_2·5_), a well-established hazard to human health.^4^ Burns are common in households using solid fuels, and fuel collection typically falls to women and girls, consuming tens of hours per week.^5^ UN Sustainable Development Goal (SDG) 3·9 aims to reduce morbidity and mortality from environmental pollution, and SDG 7·1 calls for universal access to cleaner fuels by 2030, but many countries still do not have the resources to achieve these goals.^6^

**Research in context**

**Evidence before this study**

Previous research on the global burden and mortality from household air pollution (HAP) used integrated exposure–response curves that included few HAP studies and relied on passive and active smoking data or included pooled relative risks that assumed individuals to be either exposed or unexposed to HAP. Such estimations also assumed exposure to equal concentrations of particulate matter with a diameter of less than 2·5 μm (PM_2·5_), regardless of location and fuel type used.

**Added value of this study**

This study extends previous efforts to estimate the burden attributable to HAP, with year-specific and location-specific exposures modelled by fuel type, focusing on coal, crop residues, dung, and wood. This innovative approach enabled a more precise estimation of exposure to PM_2·5_ and the associated burden of disease than previous studies, allowing us to provide comprehensive and timely estimates for 204 locations from 1990 to 2021. Increased availability of epidemiological studies and a newly developed meta-regression tool (meta-regression: Bayesian, regularised, trimmed) allowed us to expand the scope of health outcomes and discontinue the use of active and second-hand smoking data in our exposure–response estimations. Notably, we present the first global analysis to incorporate causes mediated via adverse reproductive outcomes—ie, short gestation and low birthweight. The indirect effect of HAP on reproductive health extends across the lifespan due to negative impacts on outcomes including diarrhoeal disease, respiratory disease, and other infectious and non-infectious diseases, substantially adding to the burden of disease. We also included cataract (estimated as a HAP-attributable cause for females since the Global Burden of Diseases, Injuries, and Risk Factors Study [GBD] 2010 and for males since GBD 2019) and diabetes (first included as a HAP-attributable cause in GBD 2017) in our estimations.

**Implications of all the available evidence**

Despite substantial efforts to reduce exposure and decreases in the proportion of individuals cooking with solid fuels, exposure to and burden from HAP remain high. Our findings highlight higher exposure, morbidity, and mortality than previously estimated and show that although reductions in exposure have led to a net decrease in HAP burden over time, population growth has counteracted much of the effect of reduced exposure. We found that about a third of HAP-attributable burden is mediated through adverse reproductive outcomes, leading to tens of millions of years of life lost annually. Renewed efforts and international investments are required to transition exposed communities to cleaner fuels and reduce the consequent lifelong burden of household air pollution.

Previous studies, including the Global Burden of Diseases, Injuries, and Risk Factors Study, have estimated the HAP-attributable burden of disease, but limitations relating to methods and data remain. Past exposure estimates either did not account for spatiotemporal variability^2^, ^3^ or did not adjust household exposure to the individual level.^7^ Previous estimates of burden relied on a binary exposure indicator^2^, ^4^, ^8^ and did not include type 2 diabetes or causes mediated via adverse reproductive outcomes.^8^ Here, we provide an improved methodological framework developed over the past decade that more accurately characterises exposure and risk estimates. Although previous studies assumed that different solid fuel types produce the same amount of PM_2·5_ pollution,^2^, ^8^ a known simplification, in this study we modelled fuel type-specific exposure for four distinct categories—ie, crop residues, dung, wood, and coal and charcoal—resulting in more precise PM_2·5_ exposure estimates.^9^ Moreover, we improved our exposure–response estimates by incorporating newly published studies. Thus, the meta-regression in this study does not include active and passive smoking studies, which previously served as substitutes for high exposures.^10^ We also added type 2 diabetes and causes mediated via low birthweight and short gestation to our global analysis. This study is the first to detail the GBD methodology for estimating HAP-attributable burden since the assessment by Smith and colleagues^3^ of the approach used for GBD 2010, and we present numerous methodological and input data updates.

On the basis of these methodological improvements and updated data regarding exposure and relative risk, we estimated the burden of disease attributable to HAP for 204 countries and territories from 1990 to 2021. In addition to analysing spatial patterns, we conducted decomposition analysis to better understand the drivers behind changes in the attributable burden and to evaluate the role of demographic changes versus changes in exposure to HAP over this 31-year period.^11^, ^12^ Our study adds to a growing body of work that shows the need for renewed action to reduce sustained burden from HAP. This manuscript was produced with input from the GBD Collaborator Network and in accordance with the GBD Protocol.

## Methods

### Overview

GBD 2021 complies with the GATHER statement (appendix 1 p 12).^11^ Estimating the disease burden attributable to HAP required the following steps: global estimation of the number and proportion of people exposed to HAP by year, location, and fuel type; conversion of exposure proportions to fuel-type-specific PM_2·5_ concentrations by year, location, age, and sex; generation of exposure–response curves describing the relationships between PM_2·5_ concentration and relative risks (RRs) of incidence for specific diseases; and application of a theoretical minimum risk exposure level (TMREL)—ie, the level of risk exposure that minimises disease risk at the population level. These steps allowed for quantification of HAP-specific population attributable fractions that were then used to estimate HAP-attributable burden using GBD estimates for each year, age, sex, and location.

### Proportion of population exposed to solid fuel types

In the first step, we used survey data to estimate the proportion of the population primarily using solid fuels in each location and year. For the purposes of this study, solid fuel was specified as coal and charcoal, wood, crop residues, and dung (appendix 1 pp 4–10). Estimations were based on data from household surveys (eg, Demographic and Health Surveys and Multiple Indicator Cluster Surveys), population censuses, WHO's Household Energy Database,^13^ and other sources, such as country-specific surveys. For the code used in this analysis, see appendix 1 (p 4); for sources and estimates, see appendix 2 (p 6). Count data are presented to three significant figures and rates (percentages) to one decimal place.

We used the GBD 2021 spatiotemporal Gaussian process regression tool, a three-stage non-linear model that allowed us to produce a full set of estimates of the proportion of the population using each solid fuel type for all years and locations (appendix 1 pp 4–9). The first stage of the modelling process is a linear regression informed by both exposure data and covariates, the second stage smooths data variation across locations and time by analysing the residuals, and the third stage uses Gaussian process regression to produce the final model fit. After modelling each fuel type and the overall solid fuel exposure categories, we adjusted the sum of the fuel types to fit the solid fuel category for each location and year.

### Mapping of exposure to PM_2·5_

In the second step, we estimated the mean fuel type-specific concentration (in μg/m^3^) of PM_2·5_ pollution to which individuals using solid fuels for cooking were exposed, categorised by location, year, age, and sex (appendix 1 pp 10–14; for sources and estimates, see appendix 2 pp 4-5). This model enabled us to convert the proportion of the population using a given solid fuel type to the excess concentration of PM_2·5_ to which people were exposed (above the ambient concentration) by age and sex using data from real-world observations of HAP concentrations.^14^, ^15^, ^16^ Converting exposure proportions to fuel-type specific PM_2·5_ concentrations allowed us to use these data as direct input for PM_2·5_ risk curves.

### Modelling of RR

In the third step, we modelled HAP-attributable RR for the following outcomes: lower respiratory infection; stroke; ischaemic heart disease; chronic obstructive pulmonary disease (COPD); cancers of the trachea, bronchus, and lung; type 2 diabetes; cataract; low birthweight; and short gestation (appendix 1 pp 14–44; for sources, see appendix 2 p 6). Type 2 diabetes, low birthweight, and short gestation have been added as HAP-attributable causes in GBD analyses since GBD 2010.^17^, ^18^ As the adverse reproductive outcomes low birthweight and short gestation are already risk factors, we used a mediation analysis to reattribute the portion of the burden (ie, otitis, meningitis, encephalitis, sudden infant death syndrome, upper respiratory infections, diarrhoeal diseases, neonatal disorders, and lower respiratory infections) attributable to low birthweight and short gestation to PM_2·5_ pollution instead (appendix 1 pp 40–44).^18^

For each outcome except for cataract, we calculated a risk curve on the basis of epidemiological data characterising exposure to ambient PM_2·5_ air pollution and HAP. These curves enabled us to use the output of our mapping model to calculate the location-specific relative risk for a given cause. We used the recently developed burden of proof risk function framework incorporating a meta-regression tool—meta-regression: Bayesian, regularised, trimmed (MR-BRT)^19^, ^20^—to generate flexible, non-linear RR splines (exposure–response curves), combine available RR studies while minimising the effects of outliers, characterise and correct systematic biases, account for between-study heterogeneity, adjust for confounders, and extrapolate beyond the exposure levels observed in most of the literature to the very high levels of pollution found in particular locations (appendix 1 pp 14–44). For cataract, we used the burden of proof risk function approach to calculate RR for exposed versus unexposed individuals (appendix 1 p 39).

The highest PM_2·5_ concentration attributed to HAP reported in the epidemiological literature to develop risk curves is approximately 550 μg/m^3^. However, because much higher PM_2·5_ concentrations have been documented in observational studies,^14^, ^15^, ^16^ we extrapolated our risk curves to a concentration of 1000 μg/m^3^.

### Proportional population attributable fraction calculation and burden estimation

Population attributable fractions (PAFs) were estimated together for outcomes related to both ambient pollution and HAP to account for combined exposure to both types of air pollution in a single location. The TMREL for ambient PM_2·5_ was defined as uniform distribution between 2·4 μg/m^3^ and 5·9 μg/m^3^ PM_2·5_ (appendix 1 p 10) and, therefore, assumed no incremental HAP exposure.^21^ Because cataract is a consequence of HAP only, not ambient particulate matter, unlike other outcomes, the TMREL for cataract was defined as individuals not using solid cooking fuel. The PAF quantifies the fraction of burden that could be attributed to a risk factor if the risk factor was reduced to the TMREL. To calculate PAFs, we first calculated the exposure to particulate matter as the sum of the ambient concentration of PM_2·5_ and household or indoor concentration of PM_2·5_. We used the population-weighted mean ambient PM_2·5_ concentration for a specific location and year and calculated indoor PM_2·5_ concentrations as a function of the percentage of population exposed and the concentrations of PM_2·5_ to which they were exposed as modelled by the household PM_2·5_ mapping function for each location and year. Because indoor PM_2·5_ concentrations are consistently higher than those measured by personal monitors, we developed a ratio to scale the indoor exposure to female exposure. We then used female-to-male and female-to-child ratios that we developed to estimate exposure for males and children, respectively (appendix 1 pp 10–14). RR values for the corresponding overall exposure were derived from the exposure–response curve (appendix 1 pp 14–44) and used to calculate PAFs (appendix 1 pp 45–46). We did not assume that all HAP exposure occurred at high concentrations of PM_2.5_, which would have resulted in negligible changes in RR per unit change of PM_2·5_ concentration for much of the domain of exposure; rather, we estimated the PAF for HAP on the basis of the proportion of the overall (HAP plus ambient air pollution) exposure due to HAP. This approach also avoided the potential for double counting of the ambient contribution to HAP that is estimated from the household PM_2·5_ mapping function. We estimated the attributable burden for each cause as the product of the total burden for that cause and corresponding PAF for each GBD location, year, age group, and sex. To account for uncertainties in our PAF modelling, we produced 500 simulations of all estimates and intermediate steps (appendix 2 p 7). The PAF reported is the mean of these simulations, and the uncertainty interval was calculated as the 2·5th and 97·5th percentile of the simulations. Finally, we conducted decomposition analysis^11^ to understand the causes of the observed changes in burden.

### Role of the funding source

The funder of the study had no role in study design, data collection, data analysis, data interpretation, or writing of the report. The corresponding author had full access to all the data in the study and had final responsibility for the decision to submit for publication.

## Results

### Exposure to solid fuels

**Figure 1:**
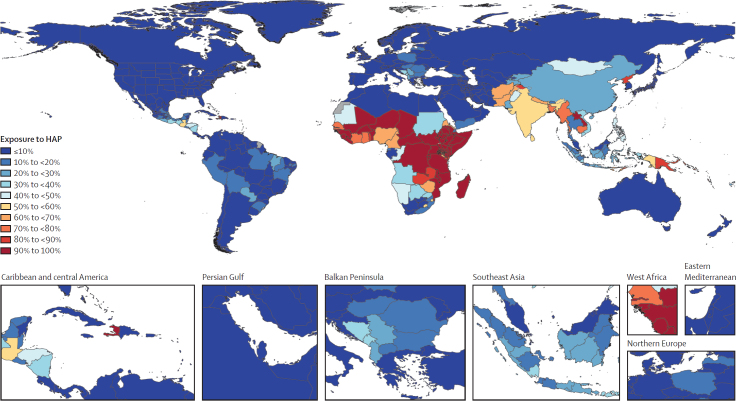
Percentage of global population exposed to HAP from solid cooking fuels, 2021 HAP=household air pollution.

In 2021, 2·67 billion (95% uncertainty interval [UI] 2·63–2·71) people were exposed to HAP from solid cooking fuels, 33·8% (95% UI 33·2–34·3) of the global population (figure 1). Although this percentage is smaller than the 56·7% (56·4–57·1) of the global population exposed to solid fuels in 1990, the total number exposed has declined by only 0·35 billion (11·6%) since then (from 3·02 billion [95% UI 3·01–3·05]). Percentages of populations exposed to solid cooking fuels in 2021 remained highest in sub-Saharan Africa (78·8%, 95% UI 77·6–80·1), south Asia (53·2%, 51·9–54·5), and southeast Asia, east Asia, and Oceania (29·4%, 28·1–30·7; figure 1). In the Americas, Haiti was an outlier, with 91·3% (89·0–93·3) of individuals exposed to HAP in 2021 (figure 1).

**Figure 2:**
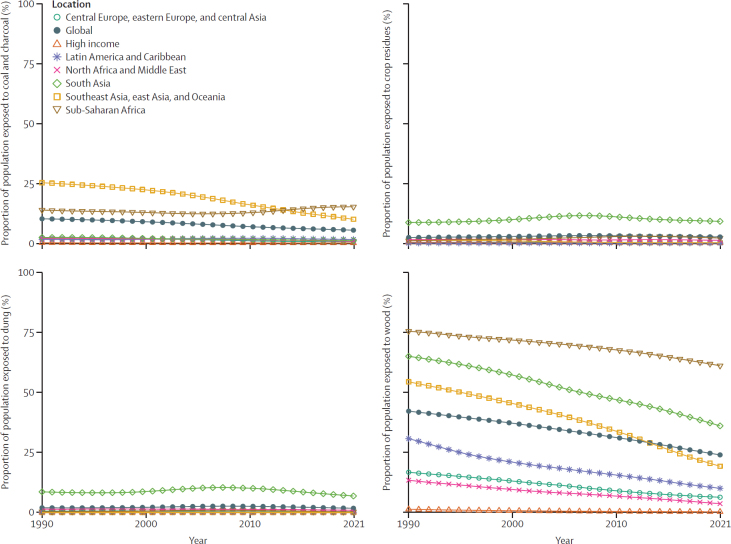
Percentages of population exposed to solid fuel types, globally and by super-region, 1990–2021

Fuel-specific modelling showed that wood was the most prevalent solid fuel worldwide, with 24·0% (23·5–24·5) of the global population exposed in 2021 (table). Coal was the second most prevalent fuel globally, with 5·4% (5·2–5·6) of the population exposed in 2021. Temporal trends showing the percentage of population exposed to each fuel type by super-region^22^ and year are shown in figure 2. Although exposure to wood remained widespread globally, exposure to coal was lower, being mainly limited to sub-Saharan Africa and southeast Asia, east Asia, and Oceania (geographical distributions are in appendix 1 p 47).

**Table:** Percentage of population exposed to HAP in 1990, 2010, and 2021 and number, percentage, and rate of DALYs attributable to HAP in 2021, globally and by super-region

	**Percentage of population exposed (95% UI)**											**DALYs (95% UI), 2021**		
	Solid cooking fuels, 1990	Solid cooking fuels, 2010	Solid cooking fuels, 2021	Coal and charcoal, 1990	Coal and charcoal, 2021	Crop residues, 1990	Crop residues, 2021	Dung, 1990	Dung, 2021	Wood, 1990	Wood, 2021	Number (in millions), all ages	DALYs (%), all ages	DALYs, rate (per 100 000), age standardised
Global	56·7% (56·4–57·1)	44·3% (44·1–44·6)	33·8% (33·3–34·3)	10·2% (9·9–10·5)	5·4% (5·2–5·6)	2·4% (2·0–2·7)	2·6% (2·3–2·9)	1·9% (1·7–2·2)	1·7% (1·5–2)	42·3% (41·7–42·8)	24·0% (23·5–24·5)	111·0 (75·1–164·0)	3·9% (2·6–5·7)	1500·3 (1028·4–2195·6)
Central Europe, eastern Europe, and central Asia	19·4% (17·9–21)	11·0% (10·2–11·8)	7·5% (6·8–8·3)	1·8% (1·4–2·2)	0·628% (0·508–0·796)	0·341% (0·233–0·471)	0·213% (0·142–0·304)	0·533% (0·404–0·683)	0·324% (0·262–0·398)	16·8% (15·4–18·3)	6·3% (5·7–7·0)	0·835 (0·277–2·77)	0·441% (0·149–1·472)	173·8 (68·4–509·8)
High-income countries	1·8% (1·6–1·9)	0·595% (0·533–0·662)	0·397% (0·340–0·460)	0·141% (0·108–0·183)	0·042% (0·034–0·053)	0·308% (0·285–0·332)	0·088% (0·059–0·127)	0·003% (0·002–0·005)	0·003% (0·002–0·006)	1·3% (1·2–1·4)	0·264% (0·222–0·313)	0·0194 (0·000377–0·139)	0·005% (0–0·041)	1·0 (0–7·8)
Latin America and Caribbean	32·5% (30·9–34·0)	18·2% (17·1–19·6)	11·7% (10·7–12·9)	1·6% (1·4–1·8)	1·6% (1·4–1·8)	0·012% (0·007–0·020)	0·015% (0·010–0·021)	0·121% (0·084–0·169)	0·059% (0·044–0·081)	30·8% (29·2–32·3)	10·1% (9·1–11·2)	1·79 (0·977–3·52)	0·853% (0·467–1·7)	316·7 (176·4–607·4)
North Africa and Middle East	18·4% (17·1–19·9)	11·0% (10·6–11·3)	6·6% (6·0–7·1)	2·0% (1·6–2·5)	0·793% (0·607–1·0)	1·5% (1·1–2·0)	1·2% (0·9–1·5)	1·4% (1·0–1·9)	0·910% (0·712–1·1)	13·4% (12·3–14·8)	3·7% (3·2–4·1)	2·5 (1·8–3·3)	1·3% (0·914–1·7)	471·9 (337·7–637·8)
South Asia	84·7% (84·0–85·4)	70·6% (70·1–71·0)	53·2% (51·9–54·5)	2·4% (2·1–2·6)	1·1% (0·947–1·2)	8·6% (7·1–10·2)	9·1% (7·8–10·3)	8·6% (7·5–9·8)	6·9% (5·7–8·1)	65·2% (63·4–67·0)	36·2% (34·5–37·8)	47·2% (31·9–64·9)	6·8% (4·6–9·3)	3213·5 (2165·4–4409·5)
Southeast Asia, east Asia, and Oceania	80·8% (80·0–81·5)	51·5% (50·7–52·4)	29·4% (28·1–30·7)	25·2% (24·4–26·1)	10·0% (9·2–10·7)	1·0% (0·814–1·2)	0·147% (0·111–0·198)	0·002% (0·002–0·002)	0·001% (0·001–0·002)	54·5% (53·5–55·6)	19·3% (18·2–20·4)	20·0 (7·93–45·6)	3·0% (1·2–7·0)	836·5 (350·4–1841·5)
Sub-Saharan Africa	90·8% (90·1–91·4)	84·3% (83·9–84·7)	78·8% (77·6–80·1)	13·8% (12·9–14·9)	15·2% (14·5–15·9)	1·0% (0·706–1·4)	2·1% (1·8–2·5)	0·326% (0·268–0·392)	0·287% (0·227–0·359)	75·7% (74·5–76·8)	61·3% (59·9–2·6)	39·2 (29·0–51·0)	6·7% (5·0–8·6)	4044·1 (3103·5–5219·5)

Some percentages and 95% UI values given to three decimal places for greater accuracy. Only solid cooking fuel is reported for 2010. DALYs=disability-adjusted life-years. HAP=household air pollution. UI=uncertainty interval.

Using crop residues as cooking fuel resulted in exposure to the highest concentrations of PM_2·5_, with an exponentiated β value of 23·3 compared with the reference (ie, clean fuel), corresponding to exposure to concentrations of PM_2·5_ 23·3 times higher than those produced by clean fuel, followed by dung (β value 10·5), wood (β value 7·3), and coal (β value 5·3; appendix 1 p 12). The mean concentration of PM_2·5_ from HAP globally was 213·6 μg/m^3^ (median 198·0 μg/m^3^) in 1990 and 84·2 μg/m^3^ (median 61·5 μg/m^3^) in 2021. The maximum concentration of PM_2·5_ from any solid cooking fuel to which individuals were exposed between 1990 and 2021 was 1660 μg/m^3^.

### RRs from HAP exposure

**Figure 3:**
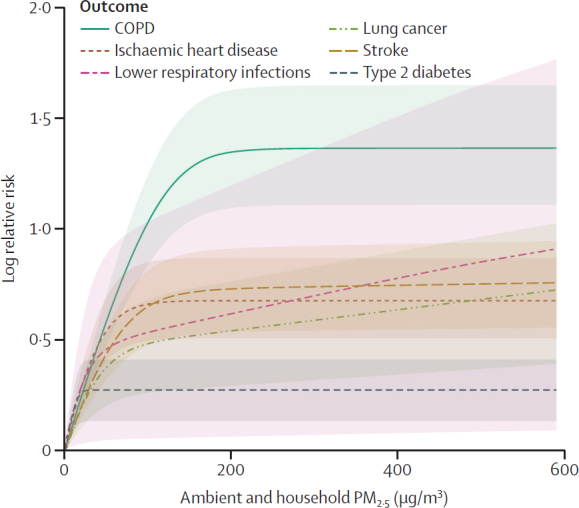
Exposure–response relationships for PM_2·5_ concentration and outcomes except for cataract Risk curves were calculated on the basis of epidemiological data characterising exposure to ambient PM_2·5_ air pollution, and household air pollution. Shading indicates 95% uncertainty interval; x-axis truncated at 600 μg/m^3^ to provide more detail at lower concentrations. COPD=chronic obstructive pulmonary disease.

Figure 3 shows splines characterising the exposure–response relationships between PM_2·5_ exposure and COPD, ischaemic heart disease, lower respiratory infections, cancers of the trachea, bronchus, and lung, stroke, and type 2 diabetes. Across the full domain of exposure, COPD showed the highest RR from HAP exposure and type 2 diabetes the lowest. For cataract, with the burden of proof risk function approach and solid fuel use treated as a dichotomous risk factor (appendix 1 p 39), RR was 2·52 (95% CI 1·36–4·50). Curves for low birthweight and short gestation are in appendix 1 (pp 40–41).

### Burden

**Figure 4:**
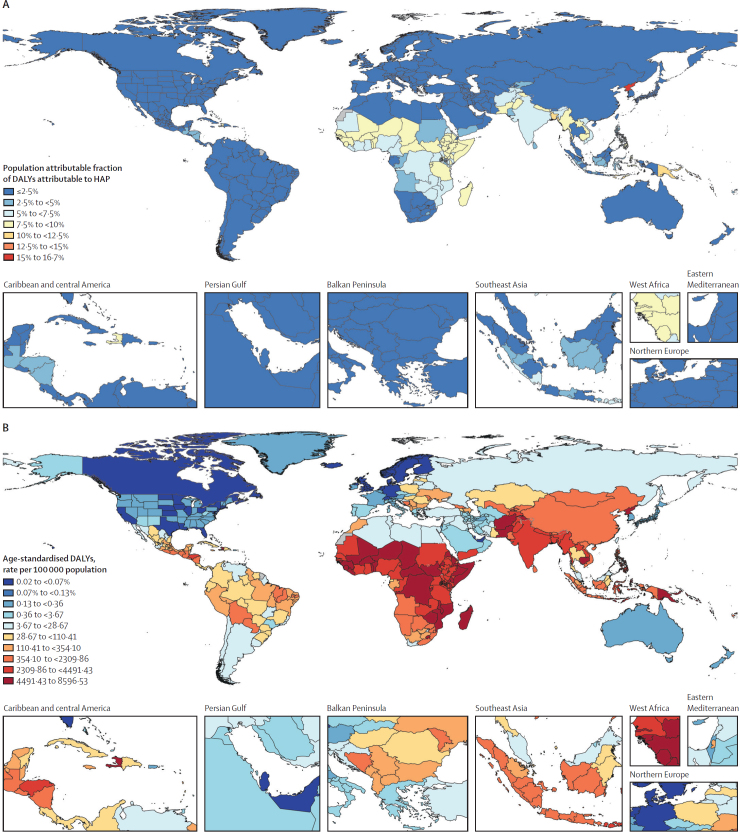
Population attributable fraction of DALYs attributable to HAP (A) and age-standardised rate per 100 000 population of DALYs attributable to HAP (B), by location, 2021 DALY=disability-adjusted life-years. HAP=household air pollution.

In 2021, 111 million (95% UI 75·1–164) disability-adjusted life-years (DALYs), 3·9% (95% UI 2·6–5·7) of the global burden from all risks, and 3·11 million (95% UI 1·90–5·19) deaths were attributable to HAP globally. HAP-attributable burden remained highest in sub-Saharan Africa and south Asia, with 4044·1 (3103·4–5219·7) and 3213·5 (2165·4–4409·4) age-standardised DALYs per 100 000 population, respectively (table, figure 4). Comparing sexes, females were exposed to higher PM_2·5_ concentrations than males (appendix 1 p 12),^16^ but, due to the higher burden of cardiovascular disease in males, HAP-attributable burden was slightly higher for males (appendix 1 p 48). All-cause PAFs for HAP have declined considerably since 1990 (appendix 1 pp 49–51), but HAP remains an important risk factor, with almost 30% of cataract burden and almost 20% of COPD burden due to HAP in 2021. Moreover, more than 0·5 million deaths in children younger than 5 years could be attributed to HAP, highlighting that almost 11% of under-5 mortality is due to HAP.

Although the percentage of global HAP-attributable DALYs has declined continuously over the past three decades, our study found that one in 26 DALYs worldwide can still be attributed to HAP. Among Level 4 risks, HAP declined from being the second most important risk for deaths in 1990 to being the eighth most important in 2021, and from being the third most important risk for DALYs in 1990 to being the eighth most important in 2021.^11^ The Socio-demographic Index^23^ (SDI; a measure that captures income per capita, education, and fertility) is predictive of the proportion of a population experiencing burden due to HAP, but there is variation within regions and SDI levels (appendix 1 p 52). Notably, many locations in southeast Asia, east Asia, and Oceania shared a similar level of burden to locations in sub-Saharan Africa, despite having higher SDI values. Additionally, countries such as Somalia and Niger had low PAFs for HAP despite their low SDI values. In contrast, PAFs for HAP were high in nations such as the Solomon Islands and Vanuatu, even though their SDIs fall within the medium range.

**Figure 5:**
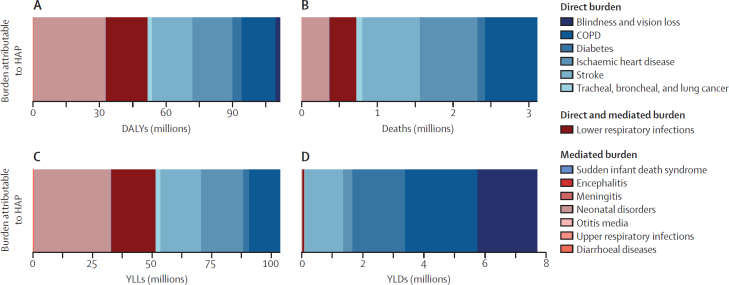
Composition of global, HAP-attributable Level 3 DALYs (A), deaths (B), YLLs (C), and YLDs (D) by disease, 2021 Non-communicable diseases are in blue; communicable, maternal, neonatal, and nutritional diseases are in red. COPD=chronic obstructive pulmonary disease. DALYs=disability-adjusted life-years. HAP=household air pollution. YLD=years lived with disability. YLL=years of life lost.

In 2021, 518·1 (95% UI 410·1–641·7) age-standardised DALYs per 100 000, about one-third of the DALYs attributable to HAP, were mediated through low birthweight and short gestation (figure 5). These mediated causes included otitis, meningitis, encephalitis, sudden infant death syndrome, upper respiratory infections, diarrhoeal diseases, neonatal disorders, and some of the lower respiratory burden. Of the Level 3 causes, ischaemic heart disease (18·2 million [95% UI 10·3–31·4]), stroke (18·2 million [10·7–30·9]), and COPD (15·6 million [9·6–25·3]) accounted for most of the direct HAP-attributable DALYs, whereas neonatal disorders were the leading cause of mediated HAP-attributable DALYs (32·1 million [25·4–39·7]). Years of life lost (YLLs) due to premature mortality and years lived with disability (YLDs), which are summed to get DALYs, showed very different patterns. Whereas YLDs consisted almost entirely of direct burden, particularly COPD (2·37 million [1·38–3·98]), cataract (1·96 million [0·612–4·03]), and type 2 diabetes (1·71 million [0·711–3·44]), the distribution of YLLs was almost evenly split between direct and mediated causes, with neonatal disorders making up the largest proportion of burden (32·0 million [25·4–39·7]). Deaths attributable to HAP were heavily dominated by direct causes, particularly ischaemic heart disease (0·763 million [0·413–1·37]), stroke (0·758 [0·433–1·31]), and COPD (0·694 million [0·412–1·18]), although neonatal disorders also contributed a notable proportion of mortality (0·356 million [0·282–0·441]).

**Figure 6:**
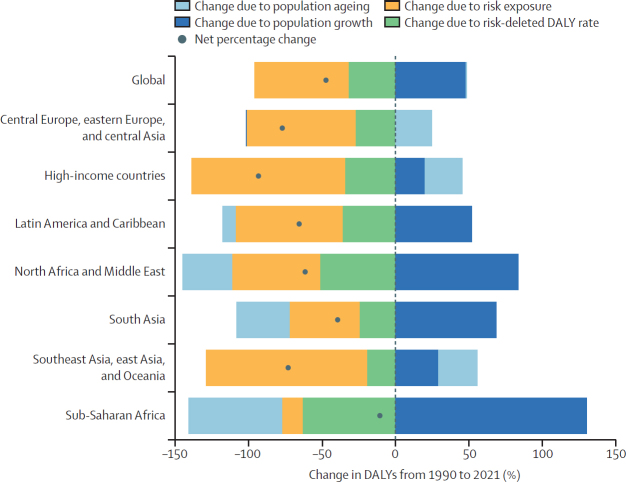
Decomposition of all-cause HAP-attributable DALYs into percent change driven by population growth, population ageing, exposure, or risk-deleted DALY rate, globally and by super-region, 1990–2021 DALYs=disability-adjusted life-years. HAP=household air pollution.

We conducted decomposition analysis to examine the changes in burden attributable to HAP from 1990 to 2021 (figure 6). This method decomposes changes to attributable burden into percentages due to population growth, population ageing, change in exposure to the risk factor, and risk-deleted burden (ie, all other changes in burden not explained by the first three categories). The result shows an overall decline in burden globally that reflects the decline in exposure for all super-regions. However, this decline has been counteracted by population growth in almost every super-region, especially sub-Saharan Africa, whereas population ageing has caused a reduction of HAP-attributable DALYs in some super-regions.

## Discussion

Our study presents timely and comprehensive estimates of exposure to PM_2·5_ pollution from HAP and of the HAP-attributable disease burden in 2021. The findings show that, first, exposure to HAP from solid fuels remained very high in sub-Saharan Africa and south Asia and, although the percentage of the global population exposed to HAP declined between 1990 and 2021, the total number of individuals exposed to HAP in 2021 remained similar to the number estimated for 1990. Second, a substantial share of the burden of disease—almost a third of DALYs—attributable to HAP was mediated through adverse reproductive outcomes, with most of this burden comprising YLLs caused by neonatal disorders. Third, even accounting for the mediated burden, overall burden from HAP is higher than previously estimated, and HAP remained a leading risk factor globally for morbidity and mortality in 2021, with 111 million (95% UI 75·1–164) DALYs and 3·1 million (1·9–5·2) deaths attributable to HAP in 2021.

Estimates of HAP exposure from solid fuels in this study were similar to previous estimates. In 2014, Bonjour and colleagues,^2^ detailing the exposure estimation methods used for GBD 2010, reported that 2·8 billion people were exposed to HAP (ie, 41% of the global population). Our analysis for GBD 2021 estimated that 3·08 billion (96% UI 3·06–3·10) people were exposed to HAP from solid fuels in 2010 (ie, 44·3% of the global population). The difference is probably the result of differing sources and modelling approaches. In GBD 2010, a single exposure indicator was used for all individuals exposed to HAP, regardless of year and location, whereas we used a spatially and temporally variable model that accounted for the type of fuel used. Additionally, Bonjour and colleagues^2^ used 586 country-year datapoints from 155 countries, whereas we used 1173 country-year sources from 161 countries (appendix 1 pp 5–7, appendix 2 p 1). A more recent study by Stoner and colleagues^7^ used a multivariate hierarchical approach to estimate the proportion of individuals exposed to polluting fuels from 1990 to 2030; these estimates comprise those reported and used by WHO^1^ for their estimates of disease burden.^24^ Stoner and colleagues’ model used six fuel types: kerosene, wood, crop waste, dung (squeezed to biomass), charcoal, and coal.^7^ As in our study, their model constrained the proportions of fuel types present so that the total never exceeded 100%. Stoner and colleagues used data already tabulated in the WHO Household Energy Database, whereas we extracted the microdata ourselves when possible and used tabulated estimates from the WHO Household Energy Database only when microdata were not available. This approach allowed us to ensure consistency in mapping survey responses to fuel categories and to extract household size, which we used to adjust results reported at the household level to a population estimate. Stoner and colleagues’ estimate of the number of people exposed to polluting fuels in 2010 (3·0 billion [95% UI 2·7–3·3]) was very similar to our own, but their estimate for 2020 (2·8 billion [2·3–3·3] people exposed, 36% [95% UI 30–43] of the global population) was higher than our estimate for the same year (2·71 billion [95% UI 2·68–2·75] people exposed, 34·7% [95% UI 34·3–35·2] of the global population). This variation is probably due to modelling differences and the fact that we adjusted for household size. Nonetheless, our estimates for 2020 were within the uncertainty reported by Stoner and colleagues, and our estimates would probably have increased if we had included kerosene in our model.

Our study found a consistently higher HAP-attributable burden than previously estimated**.** In the finalised results for GBD 2010, we reported 108 million (95% UI 84·9–133) DALYs attributable to HAP in 2010,^25^ whereas, here, we estimated 161 million (115–211) DALYs for the same year. Our estimate of deaths attributable to HAP in 2010 (4·23 million [2·94–5·77]) in this study is also higher than the number estimated in GBD 2010 (3·48 million [2·64–4·39]).^25^ These differences can probably be explained by our updated methodology, which included differing estimation methods for PM_2·5_ concentrations and risk. Since 2010, we have added cataract and type 2 diabetes,^17^ and, most notably, we have attributed a portion of the burden from causes mediated via adverse reproductive outcomes to HAP,^18^ which has led to a substantial increase in our overall estimated burden. In addition, many of the epidemiological studies used in GBD 2010, especially for non-communicable diseases, were conducted in low-pollution settings and, thus, underestimated risk when informing burden for individuals exposed to high concentrations of pollution. Finally, we estimated both proportion of population exposed to HAP and PM_2·5_ concentrations from solid fuels on the basis of the specific type of fuel used. Our analysis showed that the burning of crop residue produces higher quantities of particulate matter than does burning of dung, wood, or coal (listed in descending order of the quantities of emissions produced).

A study of HAP-attributable burden by Lee and colleagues^8^ used a random effects model to produce pooled estimates of RR for cardiorespiratory, maternal, and paediatric outcomes. Lee and colleagues estimated that HAP contributed to 1·8 million (95% CI 1·1–2·7) deaths and 60·9 million (34·6–93·3) DALYs in 2017, whereas, here, we estimated 3·25 million (95% UI 2·01–5·14) deaths and 123 million (82·1–176) DALYs attributable to HAP in the same year. Our study estimated the actual concentrations of PM_2·5_ to which individuals were exposed and assigned excess risk, whereas Lee and colleagues used a binary exposure model (ie, exposed *vs* unexposed individuals) to estimate RR. The binary exposure model included kerosene and NO_2_ from cooking fuels, whereas our risk definition included PM_2·5_ pollution from solid fuels only. As our study reflects geographical, temporal, and fuel-type variations, we believe that our estimates reflect exposure more accurately. Lee and colleagues used the exposure estimates reported by Bonjour and colleagues;^2^ thus, most of the additional burden in our analysis was probably driven by our higher estimates of exposure compared with the GBD 2010 study. Other differences were that, unlike Lee and colleagues, we included type 2 diabetes and causes mediated via adverse reproductive outcomes (which, together, constitute almost half of the estimated DALYs) and we did not model pulmonary tuberculosis or asthma. The inclusion of asthma symptoms and exacerbations is inconsistent with current risk factor analysis for air pollution in GBD, which focuses on incident disease, given the challenges in estimating short-term (ie, daily) variation in global exposure. Future iterations of the GBD study might include tuberculosis if forthcoming burden of proof analyses meet GBD inclusion criteria.^19^ Finally, we derived our RR estimates from longitudinal and case–control studies and excluded cross-sectional data (appendix 1 p 39), whereas Lee and colleagues used cross-sectional data in their estimation.

By modelling fuel-specific exposure to HAP and then mapping location-specific and year-specific exposure to real-world measurements of PM_2·5_ by age and sex, our exposure estimation overcomes some of the limitations of previous analyses. Although our previous estimates relied on an integrated exposure–response curve,^10^ our latest estimates used RR curves produced with the burden of proof risk function approach,^19^ allowing us to adjust the RR input data for confounding covariates and interstudy heterogeneity. The availability of additional epidemiological studies specifically linking ambient pollution and HAP to morbidity and mortality also allowed us to discontinue the inclusion of active and passive smoking data in our RR estimates. The inclusion of HAP-specific studies, particularly those for cardiovascular disease, stroke, and type 2 diabetes, is a major improvement over our 2010 estimates. The decomposition analysis revealed that population growth has partly counteracted the effect of reduced exposure to HAP in almost every super-region and at the global level.

As is the case with other estimates of global risk burden, our estimates have several limitations. First, our risk definition—exposure to PM_2·5_ pollution from solid fuels—did not allow us to capture other sources of HAP that have established epidemiological effects, such as kerosene,^26^, ^27^ or co-pollutants, such as carbon monoxide.^14^, ^28^ WHO estimated that in 2021, 63·1 million (95% UI 37·8–120·4) people cooked primarily with kerosene.^29^ Pollution produced by heating and lighting fuels in the home was absent from our analysis, which probably resulted in overestimation of the specific effect of pollution from cooking fuels. We also encountered several challenges concerning the input data for our exposure models. As we extracted only data representing the primary fuel used in a home, and data on secondary fuel sources are sparse, we were unable to model usage of multiple fuel types (known as stove stacking), which remains highly prevalent among communities using solid fuels.^30^, ^31^ Exposure data were also very sparse for high-income countries, eastern Europe, and Oceania; thus, estimates for these locations relied heavily on data imputation through spatiotemporal Gaussian process regression inference and might have underestimated solid fuel usage for communities who remain without access to cleaner fuels in these locations. Our estimates did not include the effects of improved stoves, although the efficacy of such interventions seems low.^32^, ^33^, ^34^, ^35^ Similarly, we were unable to capture the effects of various grades of fuel within a category; instead, we assumed equitoxicity within each fuel type. Furthermore, the uncertainty of our RR model could not account for measurement bias, selection bias, or model mis-specification bias. Another important limitation is the fact that we did not adjust for confounding bias because our PAF estimation method assumed no unmeasured confounding. The wide UIs for our RR curves, particularly at high PM_2.5_ concentrations, indicate the need for more high-quality studies. Dementia and tuberculosis were not included in our estimations, but these outcomes might be evaluated for inclusion in future GBD cycles.^36^, ^37^ Further discussion of the limitations of this study can be found in appendix 1 (pp 7, 10, 15). In summary, these omissions probably led to further underestimation of exposure, and, thus, burden in this study.

Our study confirms that, although progress has been made in reducing exposure to and burden attributable to HAP, the rapid transition of under-resourced communities to cleaner fuel sources is urgently needed, given the magnitude of current attributable disease burden. Reductions in exposure have been geographically variable, with the smallest declines in sub-Saharan Africa and south Asia. Previous studies have focused on household-level interventions, such as improved stoves, but the limited success of these programmes indicates the need for investments in community-level infrastructure. For example, the recent Household Air Pollution Intervention Network (HAPIN) trial^38^—the largest randomised controlled trial to study the switching of cooking fuels from biomass to liquefied petroleum gas—indicated reductions in exposure to PM_2·5_, but the intervention did not lead to reduced incidence of childhood pneumonia or to increased birthweight.^39^, ^40^ Importantly, in the intervention group, the median PM_2·5_ concentration was 24·2 μg/m^3^,^39^ a concentration for which our analysis still indicated substantially increased risk of low birthweight (appendix 1 pp 40–44), a possible limitation acknowledged by the authors of the HAPIN trial.^38^ Like the HAPIN trial, the Ghana Randomized Air Pollution and Health Study (GRAPHS) found that switching to liqueified petroleum gas produced no improvement in birthweight or the incidence of severe pneumonia.^35^ The median PM_2·5_ concentration to which the intervention group in GRAPHS was exposed was 45 μg/m^3^, probably due to pollution from neighbours’ cooking. A systematic review by Puzzolo and colleagues^41^ of respiratory outcomes, low birthweight, and short gestation found benefits to cooking with gas rather than solid fuels but also found that the use of gas instead of electric stoves increased the risk of pneumonia and COPD. Unlike our study, the meta-analysis by Puzzolo and colleagues included studies on heating fuel usage and cross-sectional studies; they note that cross-sectional studies provide a lower level of evidence but point to the paucity of randomised controlled trials available for inclusion. Due to the timing of the search, Puzzolo and colleagues did not include the HAPIN findings in their systematic review, but they acknowledge the study on birthweight^40^ and reiterate a limitation reported by the HAPIN authors—ie, the fuel switch was not initiated until the second trimester of pregnancy, possibly limiting the efficacy of the intervention.^39^, ^40^ These contrasting findings highlight the complexity of the healthy household energy challenge and make clear that further, high-quality studies are needed, particularly studies that focus on community-level intervention.

The high level of paediatric burden estimated in this study is a major cause for concern. Apart from the approximately 66 million DALYs of HAP-attributable burden mediated via adverse reproductive outcomes in 2021, paediatric exposure to PM_2·5_ has lifelong implications, including developmental disorders, IQ loss, and increased risk of chronic illness in adulthood.^42^ Paediatric HAP exposure also impairs educational attainment and lifetime economic potential, compounding the disadvantages already faced by children from low-income backgrounds.^43^ Although several measurement studies have indicated that females are exposed to higher concentrations of particulate matter from HAP than are males (appendix 1 pp 11–13), these differences are less pronounced than often believed. For our study, we calculated adjustment ratios on the basis of measurements in several studies, including those in the PURE-AIR study, which measured exposure to household and personal air pollution within 120 communities in eight countries.^16^ The ratios we estimated were 0·64 (95% CI 0·52–0·79) for males and 0·85 (0·67–1·09) for children. The difference observed in the PURE-AIR study was even less pronounced, with mean PM_2·5_ concentrations of 67 μg/m^3^ (95% CI 62–72) for females and 62 μg/m^3^ (58–67) for males. The overall HAP-attributable burden was higher for males than for females; this finding is the consequence of an overall higher disease burden in males than in females, especially for cardiometabolic disease. Policy makers should note that although cooking is often done by women,^5^ leading to increased exposure to HAP, all members of exposed households are harmed by HAP. HAP also makes a large contribution to ambient air pollution,^44^, ^45^ and the elimination of HAP could help affected countries to meet their goals for ambient air quality.^46^, ^47^ For example, in India, 32% of the overall contribution of residential combustion to mortality was mediated via its effect on exposure to ambient PM_2·5_.^48^ In addition, the inefficient combustion of solid cooking fuels releases black carbon and CO_2_,^16^ climate forcing agents that contribute to anthropogenic climate change.^49^ Floess and colleagues^50^ have shown that transitioning users of solid fuels to cleaner energy sources also has large co-benefits for reduction of greenhouse gas emissions. Thus, the positive effects of equitable and culturally sensitive programmes in transitioning communities from solid cooking fuels to cleaner fuels are numerous. Renewed international investment to provide under-resourced communities, particularly those in sub-Saharan Africa and south Asia, with cleaner fuels must be renewed and accelerated.

In conclusion, HAP remains a leading risk factor in many low-income and middle-income countries, exerting adverse effects on a variety of health outcomes, including cardiorespiratory and metabolic diseases, as well as on reproductive health outcomes. Our study underscores the serious health consequences linked to adverse reproductive health outcomes; these can be both fatal and long lasting and have not received adequate attention from researchers and policy makers. We hope our work further illuminates the fact that the HAP-derived burden remains a major problem for many low-income and middle-income countries. Despite steady improvements made over recent years, we argue that HAP mitigation needs to remain high up the global policy agenda and requires a multifaceted approach. Prioritising research and development for cleaner technologies, subsidising affordable clean energy appliances, and enforcing stringent emissions standards are crucial policy measures. Equally important are public awareness campaigns that educate people about the associated health risks of HAP, alongside enhanced monitoring systems that supply the data needed for evidence-based decision making. By incentivising private sector investment and ensuring cross-sector policy integration, we can establish comprehensive and effective strategies to substantially reduce HAP and its detrimental effects.^1^, ^51^

### GBD 2021 HAP Collaborators

### Contributors

### Data sharing

The data used in these analyses can be downloaded from the Global Health Data Exchange GBD 2021 website at http://ghdx.healthdata.org/gbd-2021/sources.

## Declaration of interests
