# Supplementary material for: Global, regional, and national burden of household air pollution, 1990–2021: a systematic analysis for the Global Burden of Disease Study 2021
Source: Lancet. 2025 Apr 5;405(10485):1167–81. doi: 10.1016/S0140-6736(24)02840-X (PMC11971481; doi:10.1016/S0140-6736(24)02840-X)
Supplement: Supplementary appendix 1 [file mmc1.pdf]

THE LANCET

Supplementary appendix 1

This appendix formed part of the original submission and has been peer reviewed. We post it as supplied by the authors.

Supplement to: GBD 2021 HAP Collaborators. Global, regional, and national burden of household air pollution, 1990–2021: a systematic analysis for the Global Burden of Disease Study 2021. *Lancet* 2025; published online March 18. https://doi.org/10.1016/S0140-6736(24)02840-X.

Appendix 1: Methods appendix to “Global, regional, and national burden of household air pollution, 1990-2021: a systematic examination for Global Burden of Disease 2021”

This appendix provides additional methodological details for “Global, regional, and national burden of household air pollution, 1990-2021: a systematic examination for Global Burden of Disease 2021.”

Though our work builds out from previously published GBD papers, the manuscript itself is a fully novel work. Much of this appendix has been reproduced or adapted from the GBD 2021 Risk Factors Capstone published in The Lancet earlier this year and the GBD 2019 Risk Factors Capstone published in 2020.[1,2]

1 Brauer M, Roth GA, Aravkin AY, *et al.* Global burden and strength of evidence for 88 risk factors in 204 countries and 811 subnational locations, 1990–2021: a systematic analysis for the Global Burden of Disease Study 2021. *The Lancet* 2024; **403**: 2162–203.

2 Murray CJL, Aravkin AY, Zheng P, *et al.* Global burden of 87 risk factors in 204 countries and territories, 1990–2019: a systematic analysis for the Global Burden of Disease Study 2019. *The Lancet* 2020; **396**: 1223–49.

Table of Contents

Section 1: Overview **4**

Section 2: Exposure Proportion Model **4**

Section 2.1: Overview *4*

Section 2.2: Input data *5*

Section 2.3: Limitations *7*

Section 2.4: Household size crosswalk *7*

Section 2.5: Spatiotemporal Gaussian process regression model *9*

Section 3: Theoretical minimum risk exposure level **10**

Section 4: $PM_{2.5}$ Mapping Model **10**

Section 4.1: Input Data *10*

Section 4.2: Limitations *10*

Section 4.3: Model *11*

Section 5: Relative Risk **14**

Section 5.1: Overview *14*

Section 5.2: Limitations *15*

Section 5.3: Input data *16*

Section 5.4: Risk-outcome modelling *29*

Section 5.5: Risk-outcome scoring *31*

Section 5.5.1: Overview 31

Section 5.5.2: Main approach 31

Section 5.5.3: Risk-outcome curves 32

Section 5.6: Cataract *39*

Section 5.7: Low birthweight and short gestation mediation analysis *40*

Section 6: Proportional population attributable fraction calculations **45**

Supplementary Figures S16 to S22 ...... **47**

References **53**

Section 1: Overview

Figure S1 provides an overview over the GBD 2021 household air pollution modelling pipeline, outlining different sources of input data, processes and results. For a detailed explanation of the full GBD risk estimation pipeline, please see appendix 1 section 2 of the GBD 2021 Risk Factors Capstone.[1]

All code for the procedures described below is available in the IHME UW Github Repo (https://github.com/ihmeuw/ihme-modeling/tree/main/gbd_2021).

Ambient air pollution code is available in the air_pm directory (https://github.com/ihmeuw/ihme-modeling/tree/main/gbd_2021/risk_factors_code/air_pm) and household air pollution code is available in the air _hap directory (https://github.com/ihmeuw/ihme-modeling/tree/main/gbd_2021/risk_factors_code/air_hap).

The joint ambient and household air pollution relative risk estimation code is available in the air directory (https://github.com/ihmeuw/ihme-modeling/tree/main/gbd_2021/risk_factors_code/air_hap).

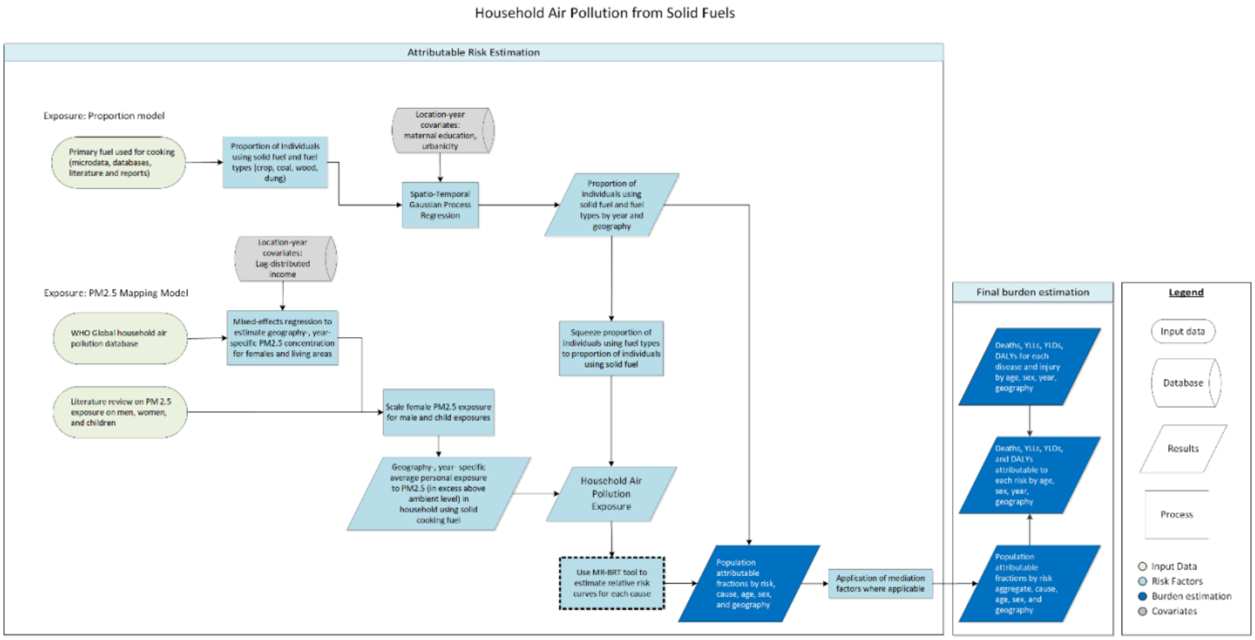

Figure S1: Overview of household air pollution burden estimation, 2021.

Section 2: Exposure Proportion Model

Section 2.1: Overview

As in the Global Burden of Disease (GBD) Study 2019, for GBD 2021, the proportion of individuals exposed to household air pollution was modelled using a three-stage modelling strategy implementing linear regression, spatiotemporal regression, and Gaussian process regression (GPR):

In Stage 1, the only linear component of the ST-GPR process, covariates are incorporated into the model, and a complete time series is generated. This allows the model to capture general trends and provide initial estimates for missing data. In Stage 2, ST-GPR performs spatiotemporal smoothing by calculating residuals—the differences between the original data and the linear predictions. These residuals are then smoothed across time, age, and space before being added back to the linear predictions. This stage introduces non-linearity into the model for the first time. Finally, in Stage 3, a Gaussian process regression (GPR) is applied, allowing the model to better align with the data. The degree to which the final regression line follows the input data is controlled by the parameters' amplitude and variance. The full spatiotemporal Gaussian process regression (ST-GPR) process is detailed in section 2.2.3 of appendix 1 of the GBD 2021 Risk Factors Capstone.[1]

For GBD 2021, we updated the HAP proportion model to disaggregate estimates of solid fuel use to estimate the proportion of individuals using primarily each of the following component fuel type categories: 1) coal or charcoal, 2) agricultural residue (crop), 3) dung, and 4) wood. With this strategy, we can more finely characterise individual exposure to $PM_{2.5}$ due to solid fuel use by applying fuel-specific mapping values to fuel-specific proportion estimates. This change addresses an important limitation in our model, in that it previously assumed equal $PM_{2.5}$ exposure for all solid fuel categories.

Fuel type-specific estimates were generated by first using ST-GPR to generate location- and year-specific estimates for coal, crop, dung, and wood. ST-GPR was also used to create estimates for the parent solid fuel category, as in GBD 2019. The first step of the ST-GPR modelling process is a mixed-effect linear regression of logit-transformed proportion of individuals using solid cooking fuels. For each of the linear models, maternal education and the proportion of population living in urban areas were used as covariates. These models also included nested random effects by GBD region and GBD super-region.

Section 2.2: Input data

Microdata sources included multi-country surveys such as Demographic and Health Surveys, Living Standards Measurement Surveys, Multiple Indicator Cluster Surveys, Performance Monitoring and Accountability 2020 surveys, and World Health Surveys as well as country-specific surveys and censuses such as the Brazil Household Sample Survey and the South Africa Population and Housing Census. To fill gaps in data coverage, after cross-referencing to ensure no duplication, we extracted tabulations from the 2017 WHO Household Energy Database[3], which contains nationally representative estimates of cooking fuel usage from over 900 sources from 161 countries, and country-specific censuses/surveys.

We systematically reviewed and re-extracted all exposure proportion model input data to ensure data quality and update mapping of fuel strings, in accordance with Shupler et al. 2018[4]. Each of the solid fuel types, coal/charcoal, crop, dung, and wood, were extracted individually and then summed calculate the parent category of solid fuel.

We matched strings describing fuel-type used to our four categories using a standardized codebook developed in conjunction with the Institute for Health Metrics and Evaluation's Local Burden of Disease team. Mixed fuel strings (e.g. "wood and other plant fuels" were mapped to the dirtiest fuel-type

mentioned in the string; we ordered the fuels from cleanest to dirtiest as: coal, wood, crop, dung). Responses such as "other" or "unknown" were dropped from the estimation. Each fuel type proportion was tabulated separately, and then all fuel type categories were summed to calculate the parent category of solid fuel. Because we model the proportion of individuals using solid fuels for cooking, we adjusted studies that only provided household observations to an estimate of individuals using a crosswalk procedure. Household weights from microdata extracted at the household level were multiplied by the household size to obtain the person weight for tabulation at the individual level. The extractions were then tabulated by indicator and national and subnational locations.

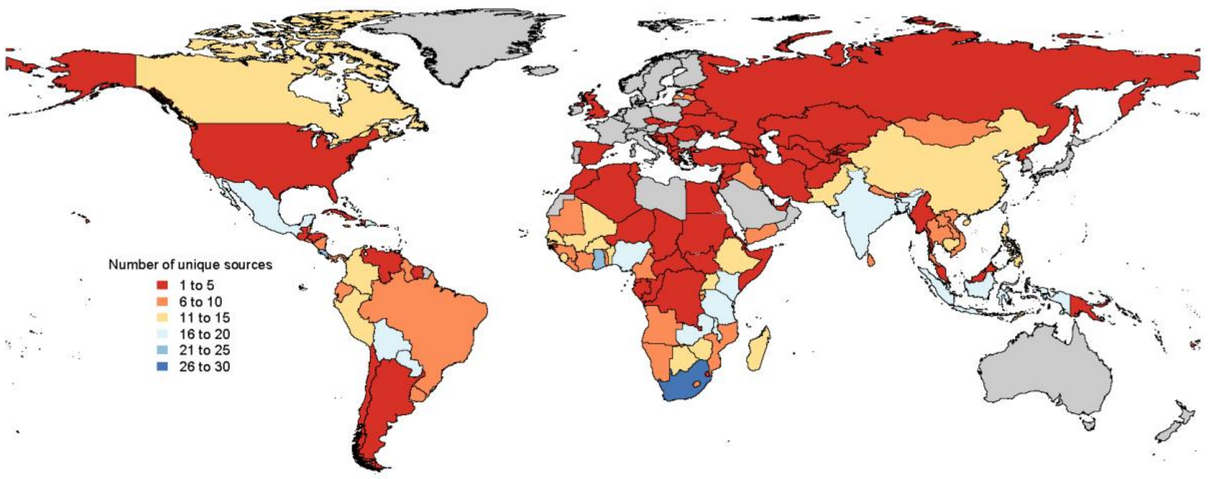

Figure S2. Geographical distribution of unique sources for the solid fuel exposure proportion model by country, 2021.

We used 1114 sources from 1980 to 2019 to inform our estimates and added 242 sources for GBD 2021 (figure S2). A full list of input sources is available in appendix 2 page 1. We excluded sources that did not include sample weights, strata, or primary sampling units (for microdata), failed to distinguish primary fuel types, estimated fuel used for purposes than cooking (eg, lighting or heating), did not report sample size, had over 15% missingness (fuel-type left blank or entered as "other" or "unknown") in either household or individual responses, reported fuel usage in physical units, or were secondary sources referencing primary analyses.

Table S1: Data inputs for exposure for Household Air Pollution.

Input data	Exposure
Site-years (total)	1173
Number of countries with data	161
Number of GBD regions[5] with data (out of 21 regions)	20
Number of GBD super-regions[5] with data (out of 7 super-regions)	7

Section 2.3: Limitations

As noted in the manuscript, our exposure estimation does not capture the use of secondary fuels (often referred to as "stove stacking"), polluting but liquid fuels (such as kerosene), or fuels used for heating and lighting. Input sources in the High-Income super-region, such as western Europe and Oceania, were very limited; thus, these areas are modelled using ST-GPR (see section 2.5). Our estimations also do not account for variations in fuel quality, shape, the combustion temperature, or the environmental or ventilation conditions present, all sources of variability in $PM_{2.5}$ concentrations. As such, we assume equitoxicity within each fuel type.

Section 2.4: Household size crosswalk

Many estimates in the WHO Energy Database and other reports quantify the proportion of households using solid fuel for cooking; however, we are interested in the proportion of individuals using solid fuel for cooking for exposure and burden assessment.

When solid fuel usage was extracted at the household level from sources that did not provide household size, we converted these estimates to the individual level to obtain the proportion of individuals who cook with solid fuel. To inform this crosswalk, we extracted and then tabulated cooking fuel at both the household and individual level when possible. We then subset our data to studies that provided household and individual fuel usage and did not report a mean of 0 or 1. The meta-regression—Bayesian, regularised, trimmed (MR-BRT) tool was used for this crosswalk model and we included 8,074 source-year-specific pairs. [6,7] We did not have sufficient data to perform this crosswalk on individual fuel types and therefore only applied the transformation to the solid fuel category.

Table S2: MR-BRT crosswalk adjustment factors for Household Air Pollution exposure

Data input	Reference or alternative case definition	Gamma	Beta coefficient, logit (95% UI)*	Adjustment factor**
Proportion of individuals	Ref	0.095	---	---
Proportion of households	Alt		-0.094 (-0.097, -0.090)	1.099 (1.094 - 1.102)

**MR-BRT crosswalk adjustments can be interpreted as the factor the alternative case definition is adjusted by to reflect what it would have been had it been measured using the reference case definition. If the log/logit beta coefficient is negative, then the alternative is adjusted up to the reference. If the log/logit beta coefficient is positive, then the alternative is adjusted down to the reference.*

***The adjustment factor column is the exponentiated negative beta coefficient. For log beta coefficients, this is the relative rate between the two case definitions. For logit beta coefficients, this is the relative odds between the two case definitions.*

We applied this coefficient to household-only solid fuel reports with the following formula:

$prop_{individ}$ = the proportion of individuals using solid fuel for cooking, and

$prop_{hh}$ = the proportion of households using solid fuel for cooking.

$$log\frac{prop_{individ}}{1-prop_{individ}} = log\frac{prop_{hh}}{1-prop_{hh}} - \beta$$

or

$$prop_{individ} = \frac{prop_{hh} * e^{-\beta}}{1-prop_{hh}+prop_{hh} * e^{-\beta}}$$

We thus inflated studies only reporting household solid fuel usage to account for bias in size between households that use solid cooking fuels and those that do not. As we hypothesized, larger households are more likely to use solid fuels for cooking. Figure S3 shows the 8,074 data points included in the crosswalk model. 10% of studies, showed as red points in figure S3, were trimmed as outliers during model fitting.

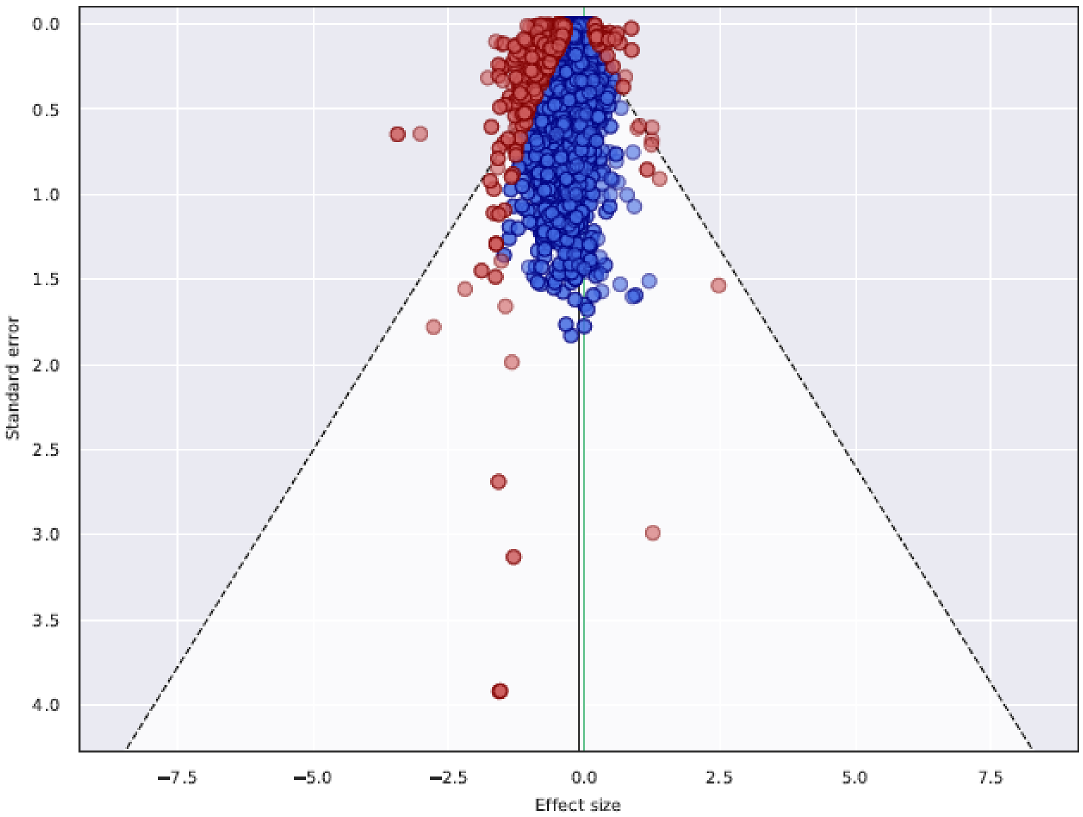

Figure S3: Funnel plot displaying data points included in crosswalk model, adjusting for household size.

Red points indicate the 10% of studies trimmed as outliers during model fitting.

$$logit(prop_{hh}) - logit(prop_{indiv})$$

Section 2.5: Spatiotemporal Gaussian process regression model

The ST-GPR modeling framework consists of three stages. First, we ran five mixed-effects linear regression models on the logit-transformed proportions of individuals using coal, crop residues, dung, wood, and any solid fuel, respectively. These models contained nested random effects by GBD region and GBD super-region and utilized maternal education per capita and proportion of population living in urban areas as predictive covariates (citation appendix 1 pp 65).[8] Results from this linear regression stage were used as priors for the following spatiotemporal and Gaussian-process regression (GPR) stages, described in detail in section 2.2.3 of the 2021 GBD Risk Factors Capstone.[1] Cross-validation (10-fold, with 20% of the data held out each time) was performed to tune the hyperparameters controlling smoothness across age, locations, and time. The estimated mean proportion for each country is found by taking 500 random simulations of the sample distribution of the GPR results and then taking the mean of these simulations. The 95% Uncertainty Interval is found by calculating the 2.5th and 97.5th percentiles of the sample distribution.

$$logit(proportion) \sim maternal\ education + urbanicity + (1|region) + (1|super - region)$$

The four solid fuel type subcategories were then adjusted (squeezed) to the overall proportion of individuals using solid fuel for cooking using the following formula, where $prop_{coal}$, $prop_{crop}$, $prop_{dung}$, $prop_{wood}$, and $prop_{solid}$ indicate the proportion of individuals using coal/charcoal, crop, dung, wood, or any type of solid fuel, respectively. Let

$$prop_{total} = prop_{coal} + prop_{wood} + prop_{crop} + prop_{dung}$$

and

$$S = prop_{total} / prop_{solid}$$

For each fuel category, with coal shown below as an example, the adjusted (squeezed) proportion is calculated as:

$$prop'_{coal} = \frac{prop_{coal}}{S}$$

This approach is an update from our GBD 2010 methodology (further refined by Smith and colleagues) where we modeled only the proportion of individuals using solid fuel.[9,10] In preliminary model iterations, we mapped mixed fuel strings (eg, “wood and agricultural residues") to the category associated with highest $PM_{2.5}$ exposure to avoid underestimating HAP exposure. However, fuel-specific ST-GPR models were unstable with this approach. We therefore excluded mixed-fuel string studies from final estimates for fuel-specific proportions, though we retained these studies when modelling the proportion of overall solid fuel use. We hope to rectify this issue in future rounds of the GBD.

Exposure proportions by fuel type and country and territory or super-region are supplied in appendix 2 pp 2 and 3, respectively.

Section 3: Theoretical minimum risk exposure level

For all HAP outcomes except cataract, burden is related to both ambient and household air pollution. These population attributable fractions (PAFs) are estimated jointly, and the theoretical minimum-risk exposure level (TMREL) is defined as a uniform distribution between 2.4 and 5.9 μg/m 3 $PM_{2.5}$ (for more details, see appendix 1 pp 82 from the GBD 2019 Risk Factors Capstone).[2] This aligns with the WHO's recommendation of 5 μg/m^3.[11] For cataract, the TMREL is defined as individuals not using solid cooking fuel.

Section 4: $PM_{2.5}$ Mapping Model

Section 4.1: Input Data

To derive relative risks from particulate matter risk curves for individuals using solid fuels for cooking, we first estimated the $PM_{2.5}$ exposure level resulting from usage of each fuel type. Input data for the HAP mapping model included indoor and personal measurement data from the WHO Global Household Air Pollution Measurements database, which contains 196 studies with measurements from 43 countries of various pollution metrics in households using solid fuel for cooking.[12,13] For GBD 2021, we also added data from the PURE-AIR study published in 2020, which includes additional measurements from 120 rural locations in Bangladesh, Chile, China, Colombia, India, Pakistan, Tanzania, and Zimbabwe.[14] The final dataset included 390 estimates from 76 studies in 47 unique locations (see appendix 2 pp 4). They comprise 3, 23, 20, 16, and 8 studies from High-income, Latin American and Caribbean, South Asia, Southeast Asia, East Asia, and Oceania, and sub-Saharan Africa, respectively. We included 281, 81, 9, and 19 measurements for indoor exposure and personal monitors for females, children (under 5), and males, respectively. 314 estimates were in households using solid fuels, 61 in households using clean fuels (gas or electricity) only, and 15 in households using a mixture of solid and clean fuels. Of measurements from households using solid fuels, we included 40, 20, 13, 155, and 86 measurements for coal, crop, dung, wood, and mixed fuels, respectively.

Section 4.2: Limitations

Because our model does not account for the presence of heating, lighting, or fuel for other uses besides cooking, it is possible that some of the $PM_{2.5}$ measured in the studies discussed above is from sources other than cooking fuels. This would lead us to overestimate the impact of the pollution from the cooking fuel(s) present.

An analysis of the residuals by fuel type did reveal that they deviate from normality at the high end of the exposure range. This is likely due to the difficulty of measuring concentrations at high exposure levels. Because our exposure-response functions change very little at high exposure levels (nearly flat), these anomalies should have little effect on our estimations of burden.

Section 4.3: Model

The following models were used to predict log-transformed estimates of excess $PM_{2.5}$ for each individual fuel type (coal, crop, dung, wood) and for the parent solid category. Predictions for the parent solid category were used only to prepare relative risk input data for analysis, not for predicting individual exposure to $PM_{2.5}$ from solid fuel use.

The model for fuel-type specific $PM_{2.5}$ mapping values utilized a log-linear, mixed-effects model with a logarithmic link function. The predictive covariates were solid fuel usage, measure group (male, female, indoor, child), 24-hour measurement period, and Lag-Distributed Income (LDI). For previous GBD cycles, we also included the Socio-demographic Index (SDI) as a variable to predict a unique value of HAP for each location and year based on development. For GBD 2021, we switched to LDI as evaluations of model fit using root mean square error indicated that LDI is a more suitable predictor of excess $PM_{2.5}$. Studies were weighted based on the square root of the sample size, and we included a random effect on study.

Prior to modelling, we subtracted the ambient $PM_{2.5}$ value in study location and year predicted in the GBD 2021 $PM_{2.5}$ exposure model to obtain the excess particulate matter for individuals using solid fuel. Details of the GBD 2021 ambient air pollution estimation methodology can be found in the GBD 2021 Risk Factors Capstone (see citation appendix 1 pp 85).[1]

Fuel types:

$$log(excess\ PM) \sim crop + \ coal + dung + wood + measure\ group + 24\ hr\ measurement + LDI + (\ 1|study)$$

Solid:

$$\log(excess\ PM) \sim solid + measure\ group + 24\ hr\ measurement + LDI + (1|study)$$

Where:

- the model is a log-linear model (modeled with MR-BRT linear_cov_model)
- solid is coded as four indicator variables (coal, crop, dung, wood) with clean as the reference category
- measure group is coded as three indicator variables (male, female, child) with indoor as the reference category
- 24 hr measurement is a binary indicator for whether the study measured 24 hours of $PM_{2.5}$ exposure
- LDI = lag-distributed income per capita (in $)
- random effect on study

The final model coefficients are included below (Table S3).

Table S3. Model parameters for conversion of exposure data to $PM_{2.5}$ values

Variable	Beta, log-space (95% CI)
Intercept	5.34 (5.16, 5.52)
Fuel type	
• Clean (ref)	
• Crop	3.15 (3.06, 3.25)
• Coal	1.66 (1.57, 1.73)
• Dung	2.35 (2.22, 2.48)
• Wood	1.99 (1.94, 2.04)
Measure group	
• Indoor (ref)	
• Female	-0.37 (-0.42, -0.32)
• Male	-0.27 (-0.36, -0.18)
• Child	-1.09 (-1.19, -1.00)
24-hour measurement	-0.68 (-0.83, -0.54)
LDI	$-2.93*10^{-4}$ ($-4.94*10^{-4}$, $-8.37*10^{-5}$)

$PM_{2.5}$ concentrations measured with "indoor", "kitchen", or "cooking area" monitors are consistently higher than concentrations measures with personal monitors worn by a study participant.[4] However, excluding all indoor measurements severely restricts the pool of studies available; instead, we include these studies and treat indoor measurements as a reference category against which personal exposure (female, male, and child) is scaled using the coefficients from our model. This allows us to combine the strength of a large dataset with the rigor of personal monitoring data.

To derive predicted personal $PM_{2.5}$ exposure values due to solid fuel usage, instead of using direct model outputs for males and children, we implemented a two-stage approach. First, fuel-specific $PM_{2.5}$ exposure values for females were derived from the above log-linear model. This step is where indoor measurements are "adjusted" to the personal (female) level by treating the indoor measurements as the reference category. Limited personal monitoring studies available for men and children (see section 4.1), so we scaled predictions for females using female-male and female-child exposure ratios to lend the strength of our model for females to our estimates for males and children. These ratios, updated with information from the 2020 PURE-AIR study[14] were 0.85 (95% CI 0.67 – 1.09) for female to child and 0.64 (0.52 – 0.79) for female to male. This marks a slight adjustment from the previous figures of 0.85 (with a range of 0.56–1.31) for female to child and 0.64 (ranging from 0.45–0.91) for female to male reported in the GBD 2019 Risk Factors Capstone (see citation appendix 1 page 122).[2] These updated findings serve as the basis for calibrating the $PM_{2.5}$ mapping model's predictions tailored to specific fuel types for these demographic groups, thereby facilitating the computation of relative risks derived from the $PM_{2.5}$ risk curves.

Table S4: HAP mapping personal monitoring input observations for female-male and female-child exposure ratios

Study	Location	Year	Pollutant	Female N	Female PM	Group	N	PM	Outdoor
Balakrishnan et al, 2004	Andhra Pradesh, Rural	2004	PM4	591	352	male	503	187	94
Gao X et al, 2009	Tibet	2009	PM2.5	52	127	male	85	111	78
Dasgupta et al, 2006	Bangladesh	2006	PM10	944	209	male	944	166	50
Devkumar et al, 2014	Nepal	2014	PM2.5	405	169	male	429	167	167
Balakrishnan et al, 2004	Andhra Pradesh, Rural	2004	PM4	591	352	child	56	262	94
Dionisio et al, 2008.	Republic of the Gambia	2008	PM2.5	13	275	child	13	219	147
Dasgupta et al, 2006	Bangladesh	2006	PM10	944	209	child	944	199	50
Gurley et al, 2013	Bangladesh	2013	PM2.5			child	37	308	
Shupler et al, 2020	Sub-Saharan Africa	2018	PM2.5	37	153	male	20	120	26.05
Shupler et al, 2020	India	2018	PM2.5	11	150	male	5	178	42.3
Shupler et al, 2020	India	2018	PM2.5	63	89	male	48	82	42.3
Shupler et al, 2020	South Asia	2018	PM2.5	5	148	male	3	147	64
Shupler et al, 2020	South Asia	2018	PM2.5	27	148	male	17	90	64
Shupler et al, 2020	South Asia	2018	PM2.5	5	147	male	2	73	64
Shupler et al, 2020	South Asia	2018	PM2.5	15	183	male	6	135	64
Shupler et al, 2020	Latin America and Caribbean	2018	PM2.5	24	39	male	12	40	27.2
Shupler et al, 2020	China	2018	PM2.5	36	71	male	35	61	58.9

Shupler et al, 2020	China	2018	PM2.5	23	94	male	21	93	58.9
Shupler et al, 2020	China	2018	PM2.5	55	45	male	47	44	58.9
Shupler et al, 2020	China	2018	PM2.5	4	64	male	3	37	58.9

Modeled $PM_{2.5}$ concentrations by fuel type for each super-region are available in appendix 2 pp 5. HAP PAFs are calculated jointly with those for ambient particulate matter pollution. Please section 6 below.

Section 5: Relative Risk

Section 5.1: Overview

In GBD 2017, we estimated the particulate-matter-attributable burden of disease based on the relation of long-term exposure to $PM_{2.5}$ with ischaemic heart disease, stroke (ischaemic and haemorrhagic), COPD, lung cancer, acute lower respiratory infection, and type 2 diabetes.[15] In GBD 2019, we added adverse birth outcomes including low birthweight and short gestation as contributors to $PM_{2.5}$-attributable burden.[2] Because these are risk factors (not outcomes) in the GBD study, we performed a mediation analysis, in which a proportion of the burden attributable to low birthweight and short gestation is attributed to $PM_{2.5}$ pollution. For GBD 2021, as in previous cycles, we used risk curves to calculate burden for ages 25+ for ischaemic heart disease, stroke (ischaemic and haemorrhagic), COPD, lung cancer, and type 2 diabetes and for all ages for acute lower respiratory infection. Burden calculation for mediated outcomes is described below (section 5.7). For all outcomes, we assume long-term, cumulative exposure. For adverse reproductive outcomes, we use studies that captured exposure during the whole pregnancy.[16]

For GBD 2021, we also completed new systematic reviews on ambient and household air pollution for LRI and cataract; our previous systematic review was conducted for GBD 2017.[15] Though previous work on $PM_{2.5}$ exposure and cardiovascular risk has relied on an age-specific relationship, our analysis of age-specific input data for this cause showed no relationship.

All exposure-response relationships were generated using the MR-BRT (Meta-Regression—Bayesian, Regularized, Trimmed) tool (section 5.4), which enables the establishment of a non-linear exposure-response relationship across the full spectrum of global exposures, incorporating shape constraints crucial for areas lacking data. MR-BRT is an advanced meta-regression tool designed to address many of the limitations of traditional approaches. One of its key strengths lies in its ability to model non-linear relationships between exposure (e.g., particulate matter) and outcomes, offering a more accurate and nuanced understanding of these complex interactions. Unlike conventional models that rely on linear or log-linear assumptions, MR-BRT leverages flexible splines of varying degrees to capture the shape of exposure-response relationships. The model further enhances precision by allowing the incorporation of varying relative risks (RR) across different exposure ranges, accommodating shifts in RR growth rates

over these ranges. The shape of the exposure-response curves can be finely tuned through the strategic placement and number of knots, as well as additional shape constraints, such as a monotonicity prior. As a result, all our exposure-response curves effectively depict non-linear relationships, as shown below.

Moreover, the MR-BRT tool permits adjustment for study-specific covariates to address between-study heterogeneity. It also facilitates automated outlier removal and integrates priors for non-linear relationships. We present a synthesized evidence score, derived from the exposure-response curve, which gauges the strength of evidence for the relationship between exposure and each health outcome (section 5.5). Particulate matter-outcome pairs are rated with three to four stars; three stars are assigned to lower respiratory infections, lung cancer, ischemic heart disease, stroke, and type-2 diabetes, while chronic obstructive pulmonary disease receives a four-star rating. This scoring system considers unexplained between-study heterogeneity, characterized by uncertainty intervals, as well as the influences of publication bias and the overall number of contributing studies.[6]

For all outcomes, except cataract, cause-specific risk curves were estimated jointly for $PM_{2.5}$ originating from both ambient and household air pollution. These curves enable us to calculate the location-specific relative risk for a given cause from the output of our mapping model. Further details, including approaches in previous iterations of the GBD, is available elsewhere[2]. Input data comprised studies on outdoor air pollution and household air pollution. Table S5 gives the relative risk source count for ambient particulate matter pollution and household air pollution input data. A complete list of input sources used for our relative risk estimation is available below in table S6 and appendix 2 pp 6.

We assume cumulative, long-term HAP exposure for all non-mediated causes. For low birthweight and short gestation, we use the $PM_{2.5}$ from the year the baby was born. However, when we calculate the population attributable fraction, we use a consistent $PM_{2.5}$ concentration and year for both mediated and non-mediated causes which is consistent with methodology across GBD risk factor estimation.

Section 5.2: Limitations

Although we did not use active smoking or secondhand smoking data to estimate $PM_{2.5}$ risk curves in GBD 2021, we still use an integrated exposure–response approach because we integrate relative risk estimates across ambient and HAP sources. The use of both source types to construct a risk curve with $PM_{2.5}$ as the exposure indicator assumes equitoxicity of particles regardless of source, despite evidence suggesting differences in health impacts by specific PM source (eg, motor vehicles, coal-fired power plant), size, and/or chemical composition. However, in the absence of sufficient estimates of source- or composition-specific exposure–response relationships and consistent and robust evidence of differential toxicity by source, integrating across all OAP and HAP studies is the approach most consistent with the current evidence, as reviewed by USA EPA and WHO.[17,18] As highlighted in the main text, our model does not capture the bias introduced by measurement, selection, or model misspecification. Additionally, though we adjust for many confounders (see section 5.4 below), we did not adjust for the bias unmeasured confounding introduces, a notable limitation.

Section 5.3: Input data

Table S5: Data inputs for relative risks for ambient and household particulate matter pollution

Input data	Relative risk
Site-years (total)	196
Number of countries with data	53
Number of GBD regions with data (out of 21 regions)	18
Number of GBD super-regions with data (out of 7 super- regions)	7

For GBD 2021 we implemented three key updates for our relative risk input data. In GBD 2017, we used relative estimates for active smoking and secondhand smoke (converting cigarettes per day to $PM_{2.5}$ exposure) to estimate relative risk predictions for $PM_{2.5}$ exposure at the highest end of the exposure–response curve. These data were included because most air pollution epidemiological studies have been performed in high-income countries which have lower levels of ambient $PM_{2.5}$ pollution. This posed a barrier to extrapolating relative risk estimates from the steep relationship at the beginning of the exposure range to locations with high exposures but no relative risk estimates, such as India and China. In GBD 2019, we incorporated estimates at high $PM_{2.5}$ levels by adding recently published ambient $PM_{2.5}$ studies conducted in China and other higher-exposure settings and additional HAP studies.[19-22] Additionally, the switch to MR-BRT splines in GBD 2019 (instead of the integrated exposure–response function employed in GBD 2017) presented a more flexible approach that allowed the curve to fit ambient and household data and removed the need for active smoking data to anchor the curve at higher exposures. The inclusion of active smoking and secondhand smoking data in previous GBD cycles required conversion from cigarettes per day to $PM_{2.5}$ exposure and introduced other differences, including differences in dose rates and those between voluntary (active smoking) and involuntary (ambient $PM_{2.5}$, household air pollution, secondhand smoke) exposures. Due to these factors, in GBD 2019, we removed active smoking data from the relative risk model's input data.[2] In GBD 2021, we also removed secondhand smoking data (cohort and case-control studies), completing the transition to only using $PM_{2.5}$ and HAP relative risk input data. This removes important sources of uncertainty in our earlier estimates[23,24] Figure S4 displays $PM_{2.5}$ risk curves from GBD 2019 and from GBD 2021, with and without secondhand smoking RR input data.

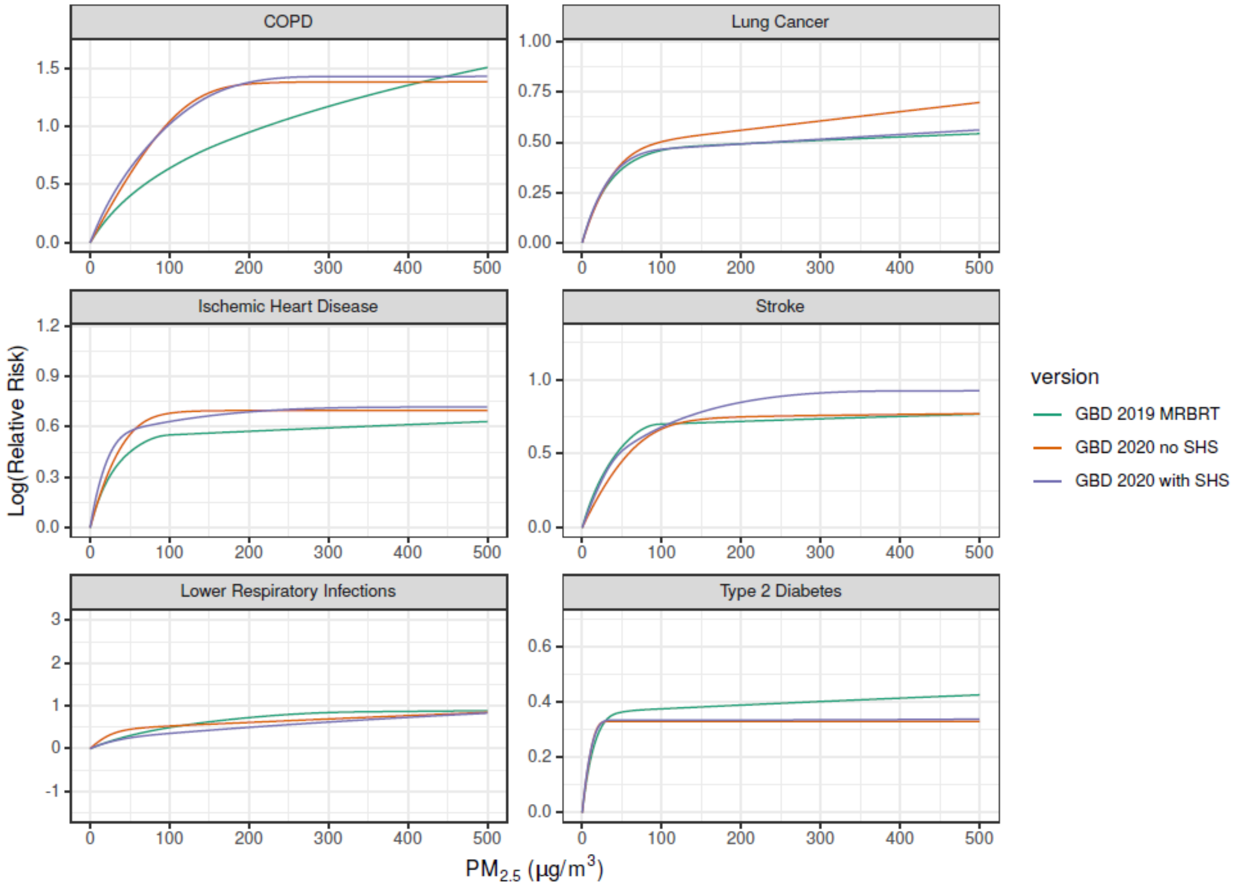

Figure S4. Relative risk curves for COPD, trachea, bronchus, and lung cancers, ischemic heart disease, stroke, lower respiratory infections, and Type 2 diabetes show for three model versions, GBD 2019 MR-BRT, GBD 2021 no secondhand smoke, GBD 2021 with secondhand smoke (note that these figures do not show uncertainty intervals for clarity of comparison—figures S5 through S10 show the uncertainty for each outcome).

For GBD 2021, as in previous GBD cycles, we created one set of cause-specific risk curves for both household air pollution and ambient particulate matter pollution as two different sources of $PM_{2.5}$. For the six non-mediated outcomes, we used results from cohort and case-control studies of ambient $PM_{2.5}$ pollution and cohort studies, case-control studies, and randomised-controlled trials of household use of solid fuel for cooking.

We conducted a literature review for studies of $PM_{2.5}$ (ambient and household air pollution) and risk of lower respiratory infection using the search string below. We searched the PubMed database for studies published between January 1, 2017, and July 22, 2020 (date of search). 32 initial results were obtained from the database, 31 of which were excluded during title-abstract and full-text screening. The remaining study was later excluded due to insufficient information reported on the study-specific exposure distribution.

Search string: ((("Air Pollution"[Mesh] OR "Particulate Matter"[Mesh] OR "air pollution"[Title/Abstract] OR "urban air pollution"[Title/Abstract] OR "ambient air pollution"[Title/Abstract] OR "airborne particulate matter"[Title/Abstract]) OR (“Air Pollution, Indoor”[Mesh] OR “Household air”[Title/Abstract] OR “Indoor air pollution”[Title/Abstract] OR “Indoor fine particulate matter”[Title/Abstract] OR “Indoor particulate matter”[Title/Abstract] OR “Indoor air quality”[Title/Abstract])) AND ("lower respiratory infection"[Title/Abstract] OR "LRI"[Title/Abstract]))

For GBD 2019, we implemented age-specific risk curves for cardiovascular diseases (ischaemic heart disease and stroke) due to evidence suggesting relative risk decreases with age for these outcomes.[24] These risk curves were created for five-year age groups from 25–29 to 95+. For GBD 2021, we dropped the use of age-specific risk curves for cardiovascular disease outcomes. Linear regressions on cardiovascular disease input data predicting log(RR) by mean cohort age, with and without random effects on study ID, were fit to ischaemic heart disease and stroke input data separately. None of these regressions showed evidence for a significant association between the two variables. Additionally, we used the MR-BRT automated covariate selection tool (detailed below) to test mean cohort age for significance as a bias covariate and found no significant results. For GBD 2021, we therefore generated a single risk curve for each of the cardiovascular outcomes and applied it across all age groups.

For all $PM_{2.5}$ outcomes, the standard error of observations from studies with multiple observations for a single cohort that reported an unstratified sample size were multiplied by the square root of n, where n is the total number of observations for a given cohort. This adjustment was made to prevent a single cohort or study from unduly weighting the final risk curve.

As in previous GBD cycles, we considered the published relative risk over a range of exposure data when fitting the risk curves. For OAP studies, the relative risk informs the curve from the 5th to the 95th percentile of observed exposure. When this is not available in the published study, we estimate the distribution from the provided information (mean and standard deviation, mean and IQR, etc.). We scale the RR to this range. For HAP studies, we allow each study to inform the curve from the Exp_{OAP} to the Exp_{OAP} + Exp_{HAP} , where Exp_{OAP} is the GBD 2019 estimate of the ambient exposure level in the study location and year, and Exp_{HAP} is the GBD 2021 estimate of the excess exposure for those who use solid fuel for cooking in the study location and year.

Table S6. GBD 2021 relative risk input sources.

Source	Reference
1	Abusalah A, Gavana M, Haidich AB, Smyrnakis E, Papadakis N, Papanikolaou A, Benos A. Low birth weight and prenatal exposure to indoor pollution from tobacco smoke and wood fuel smoke: a matched case-control study in Gaza Strip. Matern Child Health J. 2012; 16(8): 1718-27.
2	Akhtar T, Ullah Z, Khan MH, Nazli R. Chronic bronchitis in women using solid biomass fuel in rural Peshawar, Pakistan. Chest. 2007; 132(5): 1472–5.
3	Al-Sonboli N, Hart CA, Al-Aghbari N, Al-Ansi A, Ashoor O, Cuevas LE. Human metapneumovirus and respiratory syncytial virus disease in children, Yemen. Emerg Infect Dis. 2006; 12(9): 1437–9.
4	Alam DS, Chowdhury MAH, Siddiquee AT, Ahmed S, Hossain MD, Pervin S, Streatfield K, Cravioto A, Niessen LW. Adult Cardiopulmonary Mortality and Indoor Air Pollution: A 10-Year Retrospective Cohort Study in a Low-Income Rural Setting. Glob Heart. 2012; 7(3): 215–21.

5	Alexander DA, Northcross A, Karrison T, Morhasson-Bello O, Wilson N, Atalabi OM, Dutta A, Adu D, Ibigbami T, Olamijulo J, Adepoju D, Ojengbede O, Olopade CO. Pregnancy outcomes and ethanol cook stove intervention: A randomized-controlled trial in Ibadan, Nigeria. Environ Int. 2018; 111: 152-163.
6	Atkinson RW, Carey IM, Kent AJ, van Staa TP, Anderson HR, Cook DG. Long-term exposure to outdoor air pollution and the incidence of chronic obstructive pulmonary disease in a national English cohort. Occup Environ Med. 2015; 72(1): 42–8.
7	Azizi BH, Zulkifli HI, Kasim MS. Protective and risk factors for acute respiratory infections in hospitalized urban Malaysian children: a case control study. Southeast Asian J Trop Med Public Health. 1995; 26(2): 280–5.
8	Balakrishnan K, Ghosh S, Thangavel G, Sambandam S, Mukhopadhyay K, Puttaswamy N, Sadasivam A, Ramaswamy P, Johnson P, Kuppuswamy R, Natesan D, Maheshwari U, Natarajan A, Rajendran G, Ramasami R, Madhav S, Manivannan S, Nargunanadan S, Natarajan S, Saidam S, Chakraborty M, Balakrishnan L, Thanasekaraan V. Exposures to fine particulate matter (PM2.5) and birthweight in a rural-urban, mother-child cohort in Tamil Nadu, India. Environ Res. 2018; 161: 524–31.
9	Basu R, Harris M, Sie L, Malig B, Broadwin R, Green R. Effects of fine particulate matter and its constituents on low birth weight among full-term infants in California. Environ Res. 2014; 128: 42–51.
10	Basu R, Pearson D, Ebisu K, Malig B. Association between PM2.5 and PM2.5 Constituents and Preterm Delivery in California, 2000-2006. Paediatr Perinat Epidemiol. 2017; 31(5): 424-434.
11	Beelen R, Hoek G, van den Brandt PA, Goldbohm RA, Fischer P, Schouten LJ, Jerrett M, Hughes E, Armstrong B, Brunekreef B. Long-Term Effects of Traffic-Related Air Pollution on Mortality in a Dutch Cohort (NLCS-AIR Study) [Unpublished data]. Environ Health Perspect. 2008; 116(2): 196–202.
12	Beelen R, Hoek G, van den Brandt PA, Goldbohm RA, Fischer P, Schouten LJ, Jerrett M, Hughes E, Armstrong B, Brunekreef B. Long-Term Effects of Traffic-Related Air Pollution on Mortality in a Dutch Cohort (NLCS-AIR Study). Environ Health Perspect. 2008; 116(2): 196–202.
13	Beelen R, Stafoggia M, Raaschou-Nielsen O, Andersen ZJ, Xun WW, Katsouyanni K, Dimakopoulou K, Brunekreef B, Weinmayr G, Hoffmann B, Wolf K, Samoli E, Houthuijs D, Nieuwenhuijsen M, Oudin A, Forsberg B, Olsson D, Salomaa V, Lanki T, Yli-Tuomi T, Oftedal B, Aamodt G, Nafstad P, De Faire U, Pedersen NL, Östenson CG, Fratiglioni L, Penell J, Korek M, Pyko A, Eriksen KT, Tjønneland A, Becker T, Eeftens M, Bots M, Meliefste K, Wang M, Bueno-de-Mesquita B, Sugiri D, Krämer U, Heinrich J, de Hoogh K, Key T, Peters A, Cyrys J, Concin H, Nagel G, Ineichen A, Schaffner E, Probst-Hensch N, Dratva J, Ducret-Stich R, Vilier A, Clavel-Chapelon F, Stempfelet M, Grioni S, Krogh V, Tsai MY, Marcon A, Ricceri F, Sacerdote C, Galassi C, Migliore E, Ranzi A, Cesaroni G, Badaloni C, Forastiere F, Tamayo I, Amiano P, Dorronsoro M, Katsoulis M, Trichopoulou A, Vineis P, Hoek G. Long-term exposure to air pollution and cardiovascular mortality: an analysis of 22 European cohorts. Epidemiology. 2014; 25(3): 368–378.
14	Bell ML, Belanger K, Ebisu K, Gent JF, Lee HJ, Koutrakis P, Leaderer BP. Prenatal Exposure to Fine Particulate Matter and Birth Weight: Variations by Particulate Constituents and Sources. Epidemiology. 2010; 21(6): 884–91.
15	Bell ML, Ebisu K, Belanger K. Ambient Air Pollution and Low Birth Weight in Connecticut and Massachusetts. Environ Health Perspect. 2007; 115(7): 1118–24.
16	Benmarhnia T, Huang J, Basu R, Wu J, Bruckner TA. Decomposition Analysis of Black- White Disparities in Birth Outcomes: The Relative Contribution of Air Pollution and Social Factors in California. Environ Health Perspect. 2017; 125(10): 107003.
17	Bowe B, Xie Y, Li T, Yan Y, Xian H, Al-Aly Z. The 2016 global and national burden of diabetes mellitus attributable to PM2·5 air pollution. Lancet Planet Health. 2018; 2(7): e301–12.
18	Boy E, Bruce N, Delgado H. Birth weight and exposure to kitchen wood smoke during pregnancy in rural Guatemala. Environ Health Perspect. 2002; 110(1): 109-14.
19	Brauer M, Lencar C, Tamburic L, Koehoorn M, Demers P, Karr C. A cohort study of traffic-related air pollution impacts on birth outcomes. Environ Health Perspect. 2008; 116(5): 680-6.
20	Broor S, Pandey RM, Ghosh M, Maitreyi RS, Lodha R, Singhal T, Kabra SK. Risk factors for severe acute lower respiratory tract infection in under-five children. Indian Pediatr. 2001; 1361-9.
21	Burnett RT. Cox Proportional Survival Model Hazard Ratios from Census Year to 2011 for Adults Aged 25 to 89 in CanCHEC Cohort.

22	Cai J, Zhao Y, Kan J, Chen R, Martin R, van Donkelaar A, Ao J, Zhang J, Kan H, Hua J. Prenatal Exposure to Specific PM2.5 Chemical Constituents and Preterm Birth in China: A Nationwide Cohort Study. Environ Sci Technol. 2020; 54(22): 14494-14501.
23	Cakmak S, Hebbern C, Pinault L, Lavigne E, Vanos J, Crouse DL, Tjepkema M. Associations between long-term PM 2.5and ozone exposure and mortality in the Canadian Census Health and Environment Cohort (CANCHEC), by spatial synoptic classification zone. Environ Int. 2018; 111: 200-211.
24	Carey IM, Atkinson RW, Kent AJ, van Staa T, Cook DG, Anderson HR. Mortality associations with long-term exposure to outdoor air pollution in a national English cohort. Am J Respir Crit Care Med. 2013; 187(11): 1226-33.
25	Cassidy-Bushrow AE, Burmeister C, Lamerato L, Lemke LD, Mathieu M, O'Leary BF, Sperone FG, Straughen JK, Reiners JJ Jr. Prenatal airshed pollutants and preterm birth in an observational birth cohort study in Detroit, Michigan, USA. Environ Res. 2020; 189: 109845.
26	Cesaroni G, Badaloni C, Gariazzo C, Stafoggia M, Sozzi R, Davoli M, Forastiere F. Long- term exposure to urban air pollution and mortality in a cohort of more than a million adults in Rome. Environ Health Perspect. 2013; 121(3): 324–31.
27	Chang HH, Reich BJ, Miranda ML. A spatial time-to-event approach for estimating associations between air pollution and preterm birth. J R Stat Soc Ser C Appl Stat. 2013; 62(2).
28	Chen H, Burnett RT, Kwong JC, Villeneuve PJ, Goldberg MS, Brook RD, van Donkelaar A, Jerrett M, Martin RV, Brook JR, Copes R. Risk of incident diabetes in relation to long-term exposure to fine particulate matter in Ontario, Canada. Environ Health Perspect. 2013; 121(7): 804–10.
29	Chen LH, Knutsen SF, Shavlik D, Beeson WL, Petersen F, Ghamsary M, Abbey D. The association between fatal coronary heart disease and ambient particulate air pollution: Are females at greater risk? Environ Health Perspect. 2005; 113(12): 1723- 9.
30	Chen G, Guo Y, Abramson MJ, Williams G, Li S. Exposure to low concentrations of air pollutants and adverse birth outcomes in Brisbane, Australia, 2003-2013. Sci Total Environ. 2018; 622-623: 721-726.
31	Chen J, Fang J, Zhang Y, Xu Z, Byun HM, Li PH, Deng F, Guo X, Guo L, Wu S. Associations of adverse pregnancy outcomes with high ambient air pollution exposure: Results from the Project ELEFANT. Sci Total Environ. 2021; 761: 143218.
32	Clark C, Sbihi H, Tamburic L, Brauer M, Frank LD, Davies HW. Association of Long- Term Exposure to Transportation Noise and Traffic-Related Air Pollution with the Incidence of Diabetes: A Prospective Cohort Study. Environ Health Perspect. 2017; 125(8): 087025.
33	Clemens T, Turner S, Dibben C. Maternal exposure to ambient air pollution and fetal growth in North-East Scotland: A population-based study using routine ultrasound scans. Environ Int. 2017; 107: 216–26.
34	Coker E, Ghosh J, Jerrett M, Gomez-Rubio V, Beckerman B, Cockburn M, Liverani S, Su J, Li A, Kile ML, Ritz B, Molitor J. Modeling spatial effects of PM(2.5) on term low birth weight in Los Angeles County. Environ Res. 2015; 142: 354-64.
35	Collings DA, Sithole SD, Martin KS. Indoor woodsmoke pollution causing lower respiratory disease in children. Trop Doct. 1990; 20(4): 151–5.
36	Coogan PF, White LF, Yu J, Burnett RT, Seto E, Brook RD, Palmer JR, Rosenberg L, Jerrett M. PM2.5 and Diabetes and Hypertension Incidence in the Black Women's Health Study. Epidemiology. 2016; 27(2): 202–10.
37	Cramer J, Jørgensen JT, Hoffmann B, et al. Long-Term Exposure to Air Pollution and Incidence of Myocardial Infarction: A Danish Nurse Cohort Study. Environ Health Perspect. 2020;128(5):57003. doi:10.1289/EHP5818
38	Dadvand P, Ostro B, Figueras F, Foraster M, Basagaña X, Valentín A, Martinez D, Beelen R, Cirach M, Hoek G, Jerrett M, Brunekreef B, Nieuwenhuijsen MJ. Residential proximity to major roads and term low birth weight: the roles of air pollution, heat, noise, and road-adjacent trees. Epidemiology. 2014; 25(4): 518-25.
39	Darrow LA, Klein M, Strickland MJ, Mulholland JA, Tolbert PE. Ambient Air Pollution and Birth Weight in Full-Term Infants in Atlanta, 1994–2004. Environ Health Perspect. 2011; 119(5): 731–7.
40	Dennis RJ, Maldonado D, Norman S, Baena E, Martinez G. Woodsmoke exposure and risk for obstructive airways disease among women. Chest. 1996; 109(1): 115–9.

41	Dherani M, Pope D, Mascarenhas M, Smith KR, Weber M, Bruce N. Indoor air pollution from unprocessed solid fuel use and pneumonia risk in children aged under five years: a systematic review and meta-analysis. Bull World Health Organ. 2008; 86(5): 390-398C and Kossove D. and Jeena PM, Ayannusi OE, Annamalai K, Naidoo P, Coovadia HM, Guldner P. Risk factors for admission and the role of respiratory syncytial virus-specific cytotoxic T-lymphocyte responses in children with acute bronchiolitis. S Afr Med J. 2003; 93(4): 291–4.
42	Ebisu K, Bell ML. Airborne PM2.5 chemical components and low birth weight in the northeastern and mid-Atlantic regions of the United States. Environ Health Perspect. 2012; 120(12): 1746-52.
43	Ebisu K, Berman JD, Bell ML. Exposure to coarse particulate matter during gestation and birth weight in the U.S. Environ Int. 2016; 94: 519–24.
44	Ebisu K, Belanger K, Bell ML. The Association between Airborne PM2.5 Chemical Constituents and Birth Weight-Implication of Buffer Exposure Assignment. Environ Res Lett. 2014; 9(8).
45	Erickson AC, Ostry A, Chan LH, Arbour L. The reduction of birth weight by fine particulate matter and its modification by maternal and neighbourhood-level factors: a multilevel analysis in British Columbia, Canada. Environ Health. 2016; 15: 51.
46	Fleischer NL, Merialdi M, van Donkelaar A, Vadillo-Ortega F, Martin RV, Betran AP, Souza JP. Outdoor air pollution, preterm birth, and low birth weight: analysis of the world health organization global survey on maternal and perinatal health. Environ Health Perspect. 2014; 122(4): 425-30.
47	Fong KC, Kosheleva A, Kloog I, Koutrakis P, Laden F, Coull BA, Schwartz JD. Fine Particulate Air Pollution and Birthweight: Differences in Associations Along the Birthweight Distribution. Epidemiology. 2019; 30(5): 617-623.
48	Fonseca W, Kirkwood BR, Victora CG, Fuchs SR, Flores JA, Misago C. Risk factors for childhood pneumonia among the urban poor in Fortaleza, Brazil: a case-control study. Bull World Health Organ. 1996; 74(2): 199–208.
49	Galeone C, Pelucchi C, La Vecchia C, Negri E, Bosetti C, Hu J. Indoor air pollution from solid fuel use, chronic lung diseases and lung cancer in Harbin, Northeast China. Eur J Cancer Prev. 2008; 17(5): 473–8.
50	Gan WQ, FitzGerald JM, Carlsten C, Sadatsafavi M, Brauer M. Associations of ambient air pollution with chronic obstructive pulmonary disease hospitalization and mortality. Am J Respir Crit Care Med. 2013; 187(7): 721–7.
51	Gan WQ, Koehoorn M, Davies HW, Demers PA, Tamburic L, Brauer M. Long-Term Exposure to Traffic-Related Air Pollution and the Risk of Coronary Heart Disease Hospitalization and Mortality. Environ Health Perspect. 2011; 119(4): 501–7.
52	Garcia CA, Yap PS, Park HY, Weller BL. 2016. Association of long-term PM2.5 exposure with mortality using different air pollution exposure models: impacts in rural and urban California. International Journal of Environmental Health Research, 26(2), 145-15.
53	Geer LA, Weedon J, Bell ML. Ambient air pollution and term birth weight in Texas from 1998 to 2004. J Air Waste Manag Assoc. 2012; 62(11): 1285–95.
54	Gehring U, Tamburic L, Sbihi H, Davies HW, Brauer M. Impact of Noise and Air Pollution on Pregnancy Outcomes. Epidemiology. 2014; 25(3): 351–8.
55	Gehring U, Wijga AH, Fischer P, de Jongste JC, Kerkhof M, Koppelman GH, Smit HA, Brunekreef B. Traffic-related air pollution, preterm birth and term birth weight in the PIAMA birth cohort study. Environ Res. 2011; 111(1): 125–35.
56	Ger LP, Hsu WL, Chen KT, Chen CJ. Risk Factors of Lung Cancer by Histological Category in Taiwan. Anticancer Res. 1993; 13(5A): 1491–500.
57	Giorgis-Allemand L, Pedersen M, Bernard C, Aguilera I, Beelen RM, Chatzi L, Cirach M, Danileviciute A, Dedele A, van Eijsden M, Estarlich M, Fernández-Somoano A, Fernández MF, Forastiere F, Gehring U, Grazuleviciene R, Gruzieva O, Heude B, Hoek G, de Hoogh K, van den Hooven EH, Håberg SE, Iñiguez C, Jaddoe VW, Korek M, Lertxundi A, Lepeule J, Nafstad P, Nystad W, Patelarou E, Porta D, Postma D, Raaschou-Nielsen O, Rudnai P, Siroux V, Sunyer J, Stephanou E, Sørensen M, Eriksen
	KT, Tuffnell D, Varró MJ, Vrijkotte TG, Wijga A, Wright J, Nieuwenhuijsen MJ, Pershagen G, Brunekreef B, Kogevinas M, Slama R. The Influence of Meteorological Factors and Atmospheric Pollutants on the Risk of Preterm Birth. Am J Epidemiol. 2017; 185(4): 247-258.

58	Gray SC, Edwards SE, Schultz BD, Miranda ML. Assessing the impact of race, social factors and air pollution on birth outcomes: a population-based study. Environ Health. 2014; 13(1): 4.
59	Gray SC, Gelfand AE, Miranda ML. Hierarchical spatial modeling of uncertainty in air pollution and birth weight study. Stat Med. 2011; 30(17): 2187-98.
60	Guo T, Wang Y, Zhang H, Zhang Y, Zhao J, Wang Q, Shen H, Wang Y, Xie X, Wang L, Xu Z, Zhang Y, Yan D, He Y, Yang Y, Xu J, Peng Z, Ma X. The association between ambient PM2.5 exposure and the risk of preterm birth in China: A retrospective cohort study. Sci Total Environ. 2018; 633: 1453-1459.
61	Gupta D, Boffetta P, Gaborieau V, Jindal SK. Risk factors of lung cancer in Chandigarh, India. Indian J Med Res. 2001; 113: 142–50.
62	Ha S, Hu H, Roussos-Ross D, Haidong K, Roth J, Xu X. The effects of air pollution on adverse birth outcomes. Environ Res. 2014; 134: 198-204.
63	Ha S, Zhu Y, Liu D, Sherman S, Mendola P. Ambient temperature and air quality in relation to small for gestational age and term low birthweight. Environ Res. 2017; 15 5: 394-400.
64	Han Y, Ji Y, Kang S, Dong T, Zhou Z, Zhang Y, Chen M, Wu W, Tang Q, Chen T, Wang Y, Xia Y. Effects of particulate matter exposure during pregnancy on birth weight: A retrospective cohort study in Suzhou, China. Sci Total Environ. 2018; 615: 369-374.
65	Hansen AB, Ravnskjær L, Loft S, Andersen KK, Bräuner EV, Baastrup R, Yao C, Ketzel M, Becker T, Brandt J, Hertel O, Andersen ZJ. Long-term exposure to fine particulate matter and incidence of diabetes in the Danish Nurse Cohort. Environ Int. 2016; 91: 243–50.
66	Hao H, Chang HH, Holmes HA, Mulholland JA, Klein M, Darrow LA, Strickland MJ. Air Pollution and Preterm Birth in the U.S. State of Georgia (2002-2006): Associations with Concentrations of 11 Ambient Air Pollutants Estimated by Combining Community Multiscale Air Quality Model (CMAQ) Simulations with Stationary Monitor Measurements. Environ Health Perspect. 2016; 124(6): 875-80.
67	Hao Y, Strosnider H, Balluz L, Qualters JR. Geographic Variation in the Association between Ambient Fine Particulate Matter (PM2.5) and Term Low Birth Weight in the United States. Environ Health Perspect. 2016; 124(2): 250-5.
68	Harris G, Thompson WD, Fitzgerald E, Wartenberg D. The association of PM(2.5) with full term low birth weight at different spatial scales. Environ Res. 2014; 134: 427-34.
69	Hart J, Garshick E, Dockery D, Smith T, Ryan L, Laden F. Long-Term Ambient Multipollutant Exposures and Mortality. Am J Respir Crit Care Med. 2011; 183: 75–8.
70	Hart JE, Puett RC, Rexrode KM, Albert CM, Laden F. Effect Modification of Long-Term Air Pollution Exposures and the Risk of Incident Cardiovascular Disease in US Women. J Am Heart Assoc. 2015; 4(12).
71	Heft-Neal S, Burney J, Bendavid E, Burke M. Robust relationship between air quality and infant mortality in Africa. Nature. 2018; 559(7713): 2548.
72	Hertz-Picciotto I, Baker RJ, Yap P-S, Dostál M, Joad JP, Lipsett M, Greenfield T, Herr CEW, Benes I, Shumway RH, Pinkerton KE, Srám R. Early childhood lower respiratory illness and air pollution. Environ Health Perspect. 2007; 115(10): 1510-8.
73	Honda T, Pun VC, Manjourides J, et al. Associations between long-term exposure to air pollution, glycosylated hemoglobin and diabetes. Int J Hyg Environ Health. 2017, 220 (7): 1124-1132.
74	Huang C, Zhang X, Qiao Z, Guan L, Peng S, Liu J, Xie R, Zheng L. A case-control study of dietary factors in patients with lung cancer. Biomed Environ Sci. 1992; 5(3): 257–65.
75	Huang H, Woodruff TJ, Baer RJ, Bangia K, August LM, Jellife-Palowski LL, Padula AM, Sirota M. Investigation of association between environmental and socioeconomic factors and preterm birth in California. Environ Int. 2018; 121(Pt 2): 1066-1078.
76	Huang K, Liang F, Yang X, Liu F, Li J, Xiao Q, Chen J, Liu X, Cao J, Shen C, Yu L, Lu F, Wu X, Zhao L, Wu X, Li Y, Hu D, Huang J, Liu Y, Lu X, Gu D. Long term exposure to ambient fine particulate matter and incidence of stroke: prospective cohort study from the China-PAR project. BMJ. 2019; 367: l6720.
77	Huynh M, Woodruff TJ, Parker JD, Schoendorf KC. Relationships between air pollution and preterm birth in California. Paediatr Perinat Epidemiol. 2006; 20(6): 454-61.
78	Hyder A, Lee HJ, Ebisu K, Koutrakis P, Belanger K, Bell ML. PM2.5 Exposure and Birth Outcomes: Use of Satellite- and Monitor-Based Data. Epidemiology. 2014; 25(1): 58– 67.

79	Hystad P, Demers PA, Johnson KC, Carpiano RM, Brauer M. Long-term residential exposure to air pollution and lung cancer risk. Epidemiology. 2013; 24(5): 762-72.
80	Hystad P, Duong M, Brauer M, Larkin A, Arku R, Kurmi OP, Fan WQ, Avezum A, Azam I, Chifamba J, Dans A, du Plessis JL, Gupta R, Kumar R, Lanas F, Liu Z, Lu Y, Lopez- Jaramillo P, Mony P, Mohan V, Mohan D, Nair S, Puoane T, Rahman O, Lap AT, Wang Y, Wei L, Yeates K, Rangarajan S, Teo K, Yusuf S, on behalf of Prospective Urban and Rural Epidemiological (PURE) Study investigators. Health Effects of Household Solid Fuel Use: Findings from 11 Countries within the Prospective Urban and Rural Epidemiology Study [Unpublished]. Environ Health Perspect. 2019; 127(5): 57003.
81	Hystad P, Duong M, Brauer M, Larkin A, Arku R, Kurmi OP, Fan WQ, Avezum A, Azam I, Chifamba J, Dans A, du Plessis JL, Gupta R, Kumar R, Lanas F, Liu Z, Lu Y, Lopez- Jaramillo P, Mony P, Mohan V, Mohan D, Nair S, Puoane T, Rahman O, Lap AT, Wang Y, Wei L, Yeates K, Rangarajan S, Teo K, Yusuf S, on behalf of Prospective Urban and Rural Epidemiological (PURE) Study investigators. Health Effects of Household Solid Fuel Use: Findings from 11 Countries within the Prospective Urban and Rural Epidemiology Study. Environ Health Perspect. 2019; 127(5): 57003.
82	Hystad P, Larkin A, Rangarajan S, PURE country investigators, Yusuf S, Brauer M. Outdoor fine particulate matter air pollution and cardiovascular disease: Results from 747 communities across 21 countries in the PURE Study [Unpublished].
83	Jedrychowski W, Perera F, Mrozek-Budzyn D, Mroz E, Flak E, Spengler JD, Edwards S, Jacek R, Kaim I, Skolicki Z. Gender differences in fetal growth of newborns exposed prenatally to airborne fine particulate matter. Environ Res. 2009; 109(4): 447-56.
84	Jerrett M, Burnett RT, Beckerman BS, et al. 2013. Spatial analysis of air pollution and mortality in California. American Journal of Respiratory and Critical Care Medicine, 188(5), 593-599.
85	Jin C, Rossignol AM. Effects of passive smoking on respiratory illness from birth to age eighteen months, in Shanghai, People's Republic of China. J Pediatr. 1993; 123(4): 553–8.
86	Johnson AW, Aderele WI. The association of household pollutants and socio- economic risk factors with the short-term outcome of acute lower respiratory infections in hospitalized pre-school Nigerian children. Ann Trop Paediatr. 1992; 12(4): 421–32.
87	Karr C, Lumley T, Schreuder A, Davis R, Larson T, Ritz B, Kaufman J. Effects of subchronic and chronic exposure to ambient air pollutants on infant bronchiolitis. Am J Epidemiol. 2007; 165(5): 553-60.
88	Karr CJ, Rudra CB, Miller KA, Gould TR, Larson T, Sathyanarayana S, Koenig JQ. Infant exposure to fine particulate matter and traffic and risk of hospitalization for RSV bronchiolitis in a region with lower ambient air pollution. Environ Res. 2009; 109(3): 321-7.
89	Katanoda K, Sobue T, Satoh H, Tajima K, Suzuki T, Nakatsuka H, Takezaki T, Nakayama T, Nitta H, Tanabe K, Tominaga S. An association between long-term exposure to ambient air pollution and mortality from lung cancer and respiratory diseases in Japan. J Epidemiol. 2011; 21(2): 132-43.
90	Kim C, Seow WJ, Shu X-O, Bassig BA, Rothman N, Chen BE, Xiang Y-B, Hosgood HD, Ji B-T, Hu W, Wen C, Chow W-H, Cai Q, Yang G, Gao Y-T, Zheng W, Lan Q. Cooking Coal Use and All-Cause and Cause-Specific Mortality in a Prospective Cohort Study of Women in Shanghai, China. Environ Health Perspect. 2016; 124(9): 1384–9.
91	Kingsley SL, Eliot MN, Glazer K, Awad YA, Schwartz JD, Savitz DA, Kelsey KT, Marsit CJ, Wellenius GA. Maternal ambient air pollution, preterm birth and markers of fetal growth in Rhode Island: results of a hospital-based linkage study. J Epidemiol Community Health. 2017; 71(12): 1131-1136.
92	Kirwa K, McConnell-Rios R, Manjourides J, Cordero J, Alshawabekeh A, Suh HH. Low birth weight and PM2.5 in Puerto Rico. Environ Epidemiol. 2019; 3(4).
93	Kleinerman RA, Wang Z, Wang L, Metayer C, Zhang S, Brenner AV, Zhang S, Xia Y, Shang B, Lubin JH. Lung cancer and indoor exposure to coal and biomass in rural China. J Occup Environ Med. 2002; 44(4): 338–44.
94	Kloog I, Melly SJ, Ridgway WL, Coull BA, Schwartz J. Using new satellite based exposure methods to study the association between pregnancy pm2.5 exposure, premature birth and birth weight in Massachusetts. Environ Health. 2012; 11(1).
95	Ko YC, Lee CH, Chen MJ, Huang CC, Chang WY, Lin HJ, Wang HZ, Chang PY. Risk factors for primary lung cancer among non-smoking women in Taiwan. Int J Epidemiol. 1997; 26(1): 24-31.

96	Kumar N. Uncertainty in the relationship between criteria pollutants and low birth weight in Chicago. Atmos Environ. 2012; 49: 171–9.
97	Kumar S, Awasthi S, Jain A, Srivastava RC. Blood zinc levels in children hospitalized with severe pneumonia: a case control study. Indian Pediatr. 2004; 41(5): 486–91.
98	Lamichhane DK, Lee SY, Ahn K, Kim KW, Shin YH, Suh DI, Hong SJ, Kim HC. Quantile regression analysis of the socioeconomic inequalities in air pollution and birth weight. Environ Int. 2020; 142: 105875.
99	Lan Q, He X, Shen M, Tian L, Liu LZ, Lai H, Chen W, Berndt SI, Hosgood HD, Lee K-M, Zheng T, Blair A, Chapman RS. Variation in lung cancer risk by smoky coal subtype in Xuanwei, China. Int J Cancer. 2008; 123(9): 2164–9.
100	Laurent O, Hu J, Li L, Cockburn M, Escobedo L, Kleeman MJ, Wu J. Sources and contents of air pollution affecting term low birth weight in Los Angeles County, California, 2001-2008. Environ Res. 2014; 134: 488-95.
101	Laurent O, Hu J, Li L, Kleeman MJ, Bartell SM, Cockburn M, Escobedo L, Wu J. A Statewide Nested Case-Control Study of Preterm Birth and Air Pollution by Source and Composition: California, 2001-2008. Environ Health Perspect. 2016; 124(9): 1479-86.
102	Laurent O, Hu J, Li L, Kleeman MJ, Bartell SM, Cockburn M, Escobedo L, Wu J. Low birth weight and air pollution in California: Which sources and components drive the risk? Environ Int. 2016; 92-93: 471-7.
103	Laurent O, Wu J, Li L, Chung J, Bartell S. Investigating the association between birth weight and complementary air pollution metrics: a cohort study. Environ Health. 2013; 12(1).
104	Lavigne E, Yasseen AS 3rd, Stieb DM, Hystad P, van Donkelaar A, Martin RV, Brook JR, Crouse DL, Burnett RT, Chen H, Weichenthal S, Johnson M, Villeneuve PJ, Walker M. Ambient air pollution and adverse birth outcomes: Differences by maternal comorbidities. Environ Res. 2016; 148: 457-466.
105	Lavigne É, Burnett RT, Stieb DM, Evans GJ, Godri Pollitt KJ, Chen H, van Rijswijk D, Weichenthal S. Fine Particulate Air Pollution and Adverse Birth Outcomes: Effect Modification by Regional Nonvolatile Oxidative Potential. Environ Health Perspect. 2018; 126(7): 077012.
106	Le CH, Ko YC, Cheng LS, Lin YC, Lin HJ, Huang MS, Huang JJ, Kao EL, Wang HZ. The heterogeneity in risk factors of lung cancer and the difference of histologic distribution between genders in Taiwan. Cancer Causes Control. 2001; 12(4): 289– 300.
107	Lepeule J, Laden F, Dockery D, Schwartz J. Chronic exposure to fine particles and mortality: an extended follow-up of the Harvard Six Cities study from 1974 to 2009 - Unpublished data. Environ Health Perspect. 2012; 120(7): 965-70.
108	Lepeule J, Laden F, Dockery D, Schwartz J. Chronic exposure to fine particles and mortality: an extended follow-up of the Harvard Six Cities study from 1974 to 2009. Environ Health Perspect. 2012; 120(7): 965-70.
109	Li Q, Wang YY, Guo Y, Zhou H, Wang X, Wang Q, Shen H, Zhang Y, Yan D, Zhang Y, Zhang H, Li S, Chen G, Lin L, Zhao J, He Y, Yang Y, Xu J, Wang Y, Peng Z, Wang HJ, Ma X . Effect of airborne particulate matter of 2.5m or less on preterm birth: A national birth cohort study in China. Environ Int. 2018; 121(Pt 2): 1128-1136.
110	Li Q, Wang YY, Guo Y, Zhou H, Wang X, Wang QM, Shen HP, Zhang YP, Yan DH, Li S, Chen G, Lin L, He Y, Yang Y, Peng ZQ, Wang HJ, Ma X. Folic Acid Supplementation and the Association between Maternal Airborne Particulate Matter Exposure and Preterm Delivery: A National Birth Cohort Study in China. Environ Health Perspect. 2020; 128(12): 127010.
111	Li Z, Yuan X, Fu J, Zhang L, Hong L, Hu L, Liu L. Association of ambient air pollutants an d birth weight in Ningbo, 2015-2017. Environ Pollut. 2019; 249: 629-637.
112	Lim CC, Hayes RB, Ahn J, Shao Y, Silverman DT, Jones RR, Garcia C, Thurston GD. Association between long-term exposure to ambient air pollution and diabetes mortality in the US. Environ Res. 2018; 165: 330-36
113	Lin L, Li Q, Yang J, Han N, Jin C, Xu X, Liu Z, Liu J, Luo S, Raat H, Wang H. The associations of particulate matters with fetal growth in utero and birth weight: A birth cohort study in Beijing, China. Sci Total Environ. 2020; 709: 136246.
114	Lipsett MJ, Ostro BD, Reynolds P, Goldberg D, Hertz A, Jerrett M, Smith DF, Garcia C, Chang ET, Bernstein L. Long-term exposure to air pollution and cardiorespiratory disease in the California teachers study cohort [Unpublished data]. Am J Respir Crit Care Med. 2011; 184(7): 828-35.

115	Lipsett MJ, Ostro BD, Reynolds P, Goldberg D, Hertz A, Jerrett M, Smith DF, Garcia C, Chang ET, Bernstein L. Long-term exposure to air pollution and cardiorespiratory disease in the California teachers study cohort. Am J Respir Crit Care Med. 2011; 184(7): 828-35.
116	Lissowska J, Bardin-Mikolajczak A, Fletcher T, Zaridze D, Szeszenia-Dabrowska N, Rudnai P, Fabianova E, Cassidy A, Mates D, Holcatova I, Vitova V, Janout V, Mannetje A, Brennan P, Boffetta P. Lung cancer and indoor pollution from heating and cooking with solid fuels: the IARC international multicentre case-control study in Eastern/Central Europe and the United Kingdom. Am J Epidemiol. 2005; 162(4): 326– 33.
117	Luo 24, Wu B, Yi YN, Huang ZW, Lin RT. Indoor burning coal air pollution and lung cancer--a case-control study in Fuzhou, China. Lung Cancer. 1996; 14 Suppl 1: S113- 119.
118	MacIntyre EA, Gehring U, Mölter A, Fuertes E, Klümper C, Krämer U, Quass U, Hoffmann B, Gascon M, Brunekreef B, Koppelman GH, Beelen R, Hoek G, Birk M, de Jongste JC, Smit HA, Cyrys J, Gruzieva O, Korek M, Bergström A, Agius RM, de Vocht F, Simpson A, Porta D, Forastiere F, Badaloni C, Cesaroni G, Esplugues A, Fernández- Somoano A, Lerxundi A, Sunyer J, Cirach M, Nieuwenhuijsen MJ, Pershagen G, Heinrich J. Air Pollution and Respiratory Infections during Early Childhood: An Analysis of 10 European Birth Cohorts within the ESCAPE Project. Environ Health Perspect. 2014; 122(1): 107–13.
119	Mahalanabis D, Gupta S, Paul D, Gupta A, Lahiri M, Khaled MA. Risk factors for pneumonia in infants and young children and the role of solid fuel for cooking: a case-control study. Epidemiol Infect. 2002; 129(1): 65–71.
120	Melody S, Wills K, Knibbs LD, Ford J, Venn A, Johnston F. Adverse birth outcomes in Victoria, Australia in association with maternal exposure to low levels of ambient air pollution. Environ Res. 2020; 188: 109784.
121	Miller KA, Siscovick DS, Sheppard L, Shepherd K, Sullivan JH, Anderson GL, Kaufman JD. Long-term exposure to air pollution and incidence of cardiovascular events in women. N Engl J Med. 2007; 356(5): 447-58.
122	Morello-Frosch R, Jesdale BM, Sadd JL, Pastor M. Ambient air pollution exposure and full-term birth weight in California. Environ Health. 2010; 9(1).
123	Naess Ø, Nafstad P, Aamodt G, Claussen B, Rosland P. Relation between concentration of air pollution and cause-specific mortality: four-year exposures to nitrogen dioxide and particulate matter pollutants in 470 neighborhoods in Oslo, Norway. Am J Epidemiol. 2007; 165(4): 435-43.
124	Ng C, Malig B, Hasheminassab S, Sioutas C, Basu R, Ebisu K. Source apportionment of fine particulate matter and risk of term low birth weight in California: Exploring modification by region and maternal characteristics. Sci Total Environ. 2017; 605- 606: 647-654.
125	Ostro B, Hu J, Goldberg D, et al. 2015. Associations of mortality with long-term exposures to fine and ultrafine particles, species and sources: results from the California Teachers Study Cohort. Environmental Health Perspectives, 123(6), 549- 556.
126	Ottone M, Broccoli S, Parmagnani F, Giannini S, Scotto F, Bonvicini L, Luberto F, Bacco D, Trentini A, Poluzzi V, Angelini P, Colacci A, Giorgi Rossi P, Ranzi A. Source-related components of fine particulate matter and risk of adverse birth outcomes in Northern Italy. Environ Res. 2020; 186: 109564.
127	Park SK, Adar SD, O'Neill MS, Auchincloss AH, Szpiro A, Bertoni AG, Navas-Acien A, Kaufman JD, Diez-Roux AV. Long-term exposure to air pollution and type 2 diabetes mellitus in a multiethnic cohort. Am J Epidemiol. 2015; 181(5): 327–36.
128	Parker JD, Woodruff TJ, Basu R, Schoendorf KC. Air Pollution and Birth Weight Among Term Infants in California. Pediatrics. 2005; 115(1): 121–8.
129	Parker JD, Woodruff TJ. Influences of study design and location on the relationship between particulate matter air pollution and birthweight. Paediatr Perinat Epidemiol. 2008; 22(3): 214–27.
130	Parker JD, Kravets N, Vaidyanathan A. 2018. Particulate matter air pollution exposure and heart disease mortality risks by race and ethnicity in the United States: 1997 to 2009 National Health Interview Survey with mortality follow-up through 2011. Circulation, 137(16), 1688-1697.
131	Pedersen M, Giorgis-Allemand L, Bernard C, Aguilera I, Andersen AM, Ballester F, Beelen RM, Chatzi L, Cirach M, Danileviciute A, Dedele A, Eijsden Mv, Estarlich M, Fernández-Somoano A, Fernández MF, Forastiere F, Gehring U, Grazuleviciene R, Gruzieva O, Heude B, Hoek G, de Hoogh K, van den Hooven EH, Håberg SE, Jaddoe VW, Klümper C, Korek M, Krämer U, Lerchundi A, Lepeule J, Nafstad P, Nystad W, Patelarou E, Porta D, Postma D, Raaschou-Nielsen O, Rudnai P, Sunyer J, Stephanou E, Sørensen M, Thiering E, Tuffnell D, Varró MJ, Vrijkotte TG, Wijga A, Wilhelm M, Wright J, Nieuwenhuijsen MJ, Pershagen G,

	Brunekreef B, Kogevinas M, Slama R. Ambient air pollution and low birthweight: a European cohort study (ESCAPE). Lancet Respir Med. 2013; 1(9): 695-704.
132	Pereira G, Belanger K, Ebisu K, Bell ML. Fine particulate matter and risk of preterm birth in Connecticut in 2000-2006: a longitudinal study. Am J Epidemiol. 2014; 179(1): 67-74.
133	Pereira G, Bell ML, Belanger K, de Klerk N. Fine particulate matter and risk of preterm birth and pre-labor rupture of membranes in Perth, Western Australia 1997-2007: a longitudinal study. Environ Int. 2014; 73: 143-9.
134	Pinault L, Tjepkema M, Crouse DL, Weichenthal S, van Donkelaar A, Martin RV, Brauer M, Chen H, Burnett RT. Risk estimates of mortality attributed to low concentrations of ambient fine particulate matter in the Canadian community health survey cohort [Unpublished]. Environ Health. 2016; 15: 18.
135	Pinault L, Tjepkema M, Crouse DL, Weichenthal S, van Donkelaar A, Martin RV, Brauer M, Chen H, Burnett RT. Risk estimates of mortality attributed to low concentrations of ambient fine particulate matter in the Canadian community health survey cohort. Environ Health. 2016; 15(1): 18.
136	Pinault L, Brauer M, Crouse DL, et al. 2018. Diabetes status and susceptibility to the effects of PM2.5 exposure on cardiovascular mortality in a National Canadian Cohort. Epidemiology, 29(6), 784-794.
137	Pope CA, Burnett R, Thun M, Calle E, Krewski D, Ito K, Thurston G. Lung Cancer, Cardiopulmonary Mortality, and Long-term Exposure to Fine Particulate Air Pollution. JAMA. 2002; 287(9): 1132–41.
138	Pope CA, Lefler JS, Ezzati M, et al. 2019. Mortality Risk and Fine Particulate Air Pollution in a Large, Representative Cohort of US Adults. Environmental Health Perspectives, 127(7), 077007.
139	Puett RC, Hart JE, Suh H, Mittleman M, Laden F. Particulate matter exposures, mortality, and cardiovascular disease in the health professionals follow-up study. Environ Health Perspect. 2011; 119(8): 1130-5.
140	Puett RC, Hart JE, Yanosky JD, Paciorek C, Schwartz J, Suh H, Speizer FE, Laden F. Chronic fine and coarse particulate exposure, mortality, and coronary heart disease in the Nurses' Health Study. Environ Health Perspect. 2009; 117(11): 1697-701.
141	Qian Z, Liang S, Yang S, Trevathan E, Huang Z, Yang R, Wang J, Hu K, Zhang Y, Vaughn M, Shen L, Liu W, Li P, Ward P, Yang L, Zhang W, Chen W, Dong G, Zheng T, Xu S, Zhang B. Ambient air pollution and preterm birth: A prospective birth cohort study in Wuhan, China. Int J Hyg Environ Health. 2016; 219(2): 195-203.
142	Qiu H, Schooling CM, Sun S, Tsang H, Yang Y, Lee RS, Wong CM, Tian L. Long-term exposure to fine particulate matter air pollution and type 2 diabetes mellitus in elderly: A cohort study in Hong Kong. Environ Int. 2018; 113: 350-56.
143	Qiu H, Sun S, Tsang H, Wong CM, Lee RS, Schooling CM, Tian L. Fine particulate matte r exposure and incidence of stroke: A cohort study in Hong Kong. Neurology. 2017; 88(18): 1709-1717.
144	Raaschou-Nielsen O, Andersen ZJ, Beelen R, Samoli E, Stafoggia M, Weinmayr G, Hoffmann B, Fischer P, Nieuwenhuijsen MJ, Brunekreef B, Xun WW, Katsouyanni K, Dimakopoulou K, Sommar J, Forsberg B, Modig L, Oudin A, Oftedal B, Schwarze PE, Nafstad P, De Faire U, Pedersen NL, Ostenson C-G, Fratiglioni L, Penell J, Korek M, Pershagen G, Eriksen KT, Sørensen M, Tjønneland A, Ellermann T, Eeftens M, Peeters PH, Meliefste K, Wang M, Bueno-de-Mesquita B, Key TJ, de Hoogh K, Concin H, Nagel G, Vilier A, Grioni S, Krogh V, Tsai M-Y, Ricceri F, Sacerdote C, Galassi C, Migliore E, Ranzi A, Cesaroni G, Badaloni C, Forastiere F, Tamayo I, Amiano P, Dorronsoro M, Trichopoulou A, Bamia C, Vineis P, Hoek G. Air pollution and lung cancer incidence in 17 European cohorts: prospective analyses from the European Study of Cohorts for Air Pollution Effects (ESCAPE). Lancet Oncol. 2013; 14(9): 813–22.
145	Renzi M, Cerza F, Gariazzo C, et al. Air pollution and occurrence of type 2 diabetes in a large cohort study. Environ Int. 2018, 112: 68-76.
146	Robin LF, Less PS, Winget M, Steinhoff M, Moulton LH, Santosham M, Correa A. Wood-burning stoves and lower respiratory illnesses in Navajo children. Pediatr Infect Dis J. 1996; 15(10): 859–65.
147	Sapkota A, Gajalakshmi V, Jetly DH, Roychowdhury S, Dikshit RP, Brennan P, Hashibe M, Boffetta P. Indoor air pollution from solid fuels and risk of hypopharyngeal/laryngeal and lung cancers: a multicentric case-control study from India. Int J Epidemiol. 2008; 37(2): 321–8.
148	Sasco AJ, Merrill RM, Dari I, Benhaïm-Luzon V, Carriot F, Cann CI, Bartal M. A case- control study of lung cancer in Casablanca, Morocco. Cancer Causes Control. 2002; 13(7): 609–16.
149	Savitha MR, Nandeeshwara SB, Pradeep Kumar MJ, ul-Haque F, Raju CK. Modifiable risk factors for acute lower respiratory tract infections. Indian J Pediatr. 2007; 74(5): 477–82.

150	Savitz DA, Bobb JF, Carr JL, Clougherty JE, Dominici F, Elston B, Ito K, Ross Z, Yee M, Matte TD. Ambient Fine Particulate Matter, Nitrogen Dioxide, and Term Birth Weight in New York, New York. Am J Epidemiol. 2014; 179(4): 457–66.
151	Schembari A, de Hoogh K, Pedersen M, Dadvand P, Martinez D, Hoek G, Petherick ES, Wright J, Nieuwenhuijsen MJ. Ambient Air Pollution and Newborn Size and Adiposity at Birth: Differences by Maternal Ethnicity (the Born in Bradford Study Cohort). Environ Health Perspect. 2015; 123(11): 1208-15.
152	Sezer H, Akkurt I, Guler N, Marakoglu K, Berk S. A case-control study on the effect of exposure to different substances on the development of COPD. Ann Epidemiol. 2006; 16(1): 59–62.
153	Shah N, Ramankutty V, Premila PG, Sathy N. Risk factors for severe pneumonia in children in south Kerala: a hospital-based case-control study. J Trop Pediatr. 1994; 40(4): 201–6.
154	Shang L, Huang L, Yang L, Leng L, Qi C, Xie G, Wang R, Guo L, Yang W, Chung MC. Impact of air pollution exposure during various periods of pregnancy on term birth weight: a large-sample, retrospective population-based cohort study. Environ Sci Pollut Res Int. 2021; 28(3): 3296-3306.
155	Shen M, Chapman RS, Vermeulen R, Tian L, Zheng T, Chen BE, Engels EA, He X, Blair A, Lan Q. Coal use, stove improvement, and adult pneumonia mortality in Xuanwei, China: a retrospective cohort study. Environ Health Perspect. 2009; 117(2): 261–6.
156	Sheridan P, Ilango S, Bruckner TA, Wang Q, Basu R, Benmarhnia T. Ambient Fine Particulate Matter and Preterm Birth in California: Identification of Critical Exposure Windows. Am J Epidemiol. 2019; 188(9): 1608-1615.
157	Siddiqui AR, Gold EB, Yang X, Lee K, Brown KH, Bhutta ZA. Prenatal exposure to wood fuel smoke and low birth weight. Environ Health Perspect. 2008; 116(4): 543-9.
158	Smith KR, McCracken JP, Weber MW, Hubbard A, Jenny A, Thompson LM, Balmes J, Diaz A, Arana B, Bruce N. Effect of reduction in household air pollution on childhood pneumonia in Guatemala (RESPIRE): a randomised controlled trial. Lancet. 2011; 378(9804): 1717-26.
159	Smith RB, Fecht D, Gulliver J, Beevers SD, Dajnak D, Blangiardo M, Ghosh RE, Hansell AL, Kelly FJ, Anderson HR, Toledano MB. Impact of London's road traffic air and noise pollution on birth weight: retrospective population based cohort study. BMJ. 2017; 359: j5299.
160	Stafoggia M, Cesaroni G, Peters A, Andersen ZJ, Badaloni C, Beelen R, Caracciolo B, Cyrys J, de Faire U, de Hoogh K, Eriksen KT, Fratiglioni L, Galassi C, Gigante B, Havulinna AS, Hennig F, Hilding A, Hoek G, Hoffmann B, Houthuijs D, Korek M, Lanki T, Leander K, Magnusson PK, Meisinger C, Migliore E, Overvad K, Ostenson C-G, Pedersen NL, Pekkanen J, Penell J, Pershagen G, Pundt N, Pyko A, Raaschou-Nielsen O, Ranzi A, Ricceri F, Sacerdote C, Swart WJR, Turunen AW, Vineis P, Weimar C, Weinmayr G, Wolf K, Brunekreef B, Forastiere F. Long-term exposure to ambient air pollution and incidence of cerebrovascular events: results from 11 European cohorts within the ESCAPE project. Environ Health Perspect. 2014; 122(9): 919–25.
161	Starling AP, Moore BF, Thomas DSK, Peel JL, Zhang W, Adgate JL, Magzamen S, Martenies SE, Allshouse WB, Dabelea D. Prenatal exposure to traffic and ambient air pollution and infant weight and adiposity: The Healthy Start study. Environ Res. 2020; 182: 109130.
162	Stieb DM, Chen L, Beckerman BS, Jerrett M, Crouse DL, Omariba DW, Peters PA, van Donkelaar A, Martin RV, Burnett RT, Gilbert NL, Tjepkema M, Liu S, Dugandzic RM. Associations of Pregnancy Outcomes and PM2.5 in a National Canadian Study. Environ Health Perspect. 2016; 124(2): 243-9.
163	Strickland MJ, Lin Y, Darrow LA, Warren JL, Mulholland JA, Chang HH. Associations Between Ambient Air Pollutant Concentrations and Birth Weight: A Quantile Regression Analysis. Epidemiology. 2019; 30(5): 624-632.
164	Sun Z, Yang L, Bai X, Du W, Shen G, Fei J, Wang Y, Chen A, Chen Y, Zhao M. Maternal ambient air pollution exposure with spatial-temporal variations and preterm birth risk assessment during 2013-2017 in Zhejiang Province, China. Environ Int. 2019; 133(Pt B): 105242.
165	Tapia VL, Vasquez BV, Vu B, Liu Y, Steenland K, Gonzales GF. Association between maternal exposure to particulate matter (PM2.5) and adverse pregnancy outcomes in Lima, Peru. J Expo Sci Environ Epidemiol. 2020; 30(4): 689-697.
166	Thompson LM, Bruce N, Eskenazi B, Diaz A, Pope D, Smith KR. Impact of reduced maternal exposures to wood smoke from an introduced chimney stove on newborn birth weight in rural Guatemala. Environ Health Perspect. 2011; 119(10): 1489-94.

167	Thurston GD, Ahn J, Cromar KR, Shao Y, Reynolds HR, Jerrett M, Lim CC, Shanley R, Park Y, Hayes RB. Ambient Particulate Matter Air Pollution Exposure and Mortality in the NIH-AARP Diet and Health Cohort [Unpublished]. Environ Health Perspect. 2016; 124(4): 484-90.
168	Tielsch JM, Katz J, Thulasiraj RD, Coles CL, Sheeladevi S, Yanik EL, Rahmathullah L. Exposure to indoor biomass fuel and tobacco smoke and risk of adverse reproductive outcomes, mortality, respiratory morbidity and growth among newborn infants in south India. Int J Epidemiol. 2009; 38(5): 1351-63.
169	To T, Zhu J, Villeneuve PJ, Simatovic J, Feldman L, Gao C, Williams D, Chen H, Weichenthal S, Wall C, Miller AB. Chronic disease prevalence in women and air pollution--A 30-year longitudinal cohort study. Environ Int. 2015; 80: 26–32.
170	Tseng E, Ho W-C, Lin M-H, Cheng T-J, Chen P-C, Lin H-H. Chronic exposure to particulate matter and risk of cardiovascular mortality: cohort study from Taiwan. BMC Public Health. 2015; 15: 936.
171	Turner MC, Jerrett M, Pope CA 3rd, Krewski D, Gapstur SM, Diver WR, Beckerman BS, Marshall JD, Su J, Crouse DL, Burnett RT. Long-term ozone exposure and mortality in a large prospective study. Am J Respir Crit Care Med. 2016; 193(10): 1134-42.
172	Turner MC, Krewski D, Pope CA, et al. 2011. Long-term ambient fine particulate matter air pollution and lung cancer in a large cohort of never-smokers. American Journal of Respiratory and Critical Care Medicine, 184(12), 1374-1381.
173	Victora CG, Fuchs SC, Flores JA, Fonseca W, Kirkwood B. Risk factors for pneumonia among children in a Brazilian metropolitan area. Pediatrics. 1994; 977-85.
174	Villeneuve PJ, Weichenthal SA, Crouse D, Miller AB, To T, Martin RV, van Donkelaar A, Wall C, Burnett RT. Long-term exposure to fine particulate matter air pollution and mortality among Canadian women. Epidemiology. 2015; 26(4): 536-45.
175	Wang Q, Benmarhnia T, Zhang H, Knibbs LD, Sheridan P, Li C, Bao J, Ren M, Wang S, H e Y, Zhang Y, Zhao Q, Huang C. Identifying windows of susceptibility for maternal exposure to ambient air pollution and preterm birth. Environ Int. 2018; 121(Pt 1): 317-324.
176	Wayse V, Yousafzai A, Mogale K, Filteau S. Association of subclinical vitamin D deficiency with severe acute lower respiratory infection in Indian children under 5 y. Eur J Clin Nutr. 2004; 58(4): 563–7.
177	Weichenthal S, Villeneuve PJ, Burnett RT, van Donkelaar A, Martin RV, Jones RR, DellaValle CT, Sandler DP, Ward MH, Hoppin JA. Long-term exposure to fine particulate matter: association with nonaccidental and cardiovascular mortality in the agricultural health study cohort. Environ Health Perspect. 2014; 122(6): 609-15.
178	Weinmayr G, Hennig F, Fuks K, Nonnemacher M, Jakobs H, Möhlenkamp S, Erbel R, Jöckel K-H, Hoffmann B, Moebus S, Heinz Nixdorf Recall Investigator Group. Long- term exposure to fine particulate matter and incidence of type 2 diabetes mellitus in a cohort study: effects of total and traffic-specific air pollution. Environ Health. 2015; 14: 53.
179	Wesley AG, Loening WE. Assessment and 2-year follow-up of some factors associated with severity of respiratory infections in early childhood. S Afr Med J. 1996; 86(4): 365–8.
180	Wilhelm M, Ghosh JK, Su J, Cockburn M, Jerrett M, Ritz B. Traffic-related air toxics and preterm birth: a population-based case-control study in Los Angeles County, California. Environ Health. 2011; 10: 89.
181	Wong CM, Lai HK, Tsang H, Thach TQ, Thomas GN, Lam KBH, Chan KP, Yang L, Lau AKH, Ayres JG, Lee SY, Man Chan W, Hedley AJ, Lam TH. Satellite-Based Estimates of Long-Term Exposure to Fine Particles and Association with Mortality in Elderly Hong Kong Residents. Environ Health Perspect. 2015; 123(11): 1167-72.
182	Wu AH, Henderson BE, Pike MC, Yu MC. Smoking and other risk factors for lung cancer in women. J Natl Cancer Inst. 1985; 74(4): 747-51.
183	Wu J, Wilhelm M, Chung J, Ritz B. Comparing exposure assessment methods for traffic-related air pollution in an adverse pregnancy outcome study. Environ Res. 2011; 111(5): 685-92.
184	Wu H, Jiang B, Geng X, Zhu P, Liu Z, Cui L, Yang L. Exposure to fine particulate matter during pregnancy and risk of term low birth weight in Jinan, China, 2014-2016. Int J Hyg Environ Health. 2018; 221(2): 183-190.
185	Wylie BJ, Coull BA, Hamer DH, Singh MP, Jack D, Yeboah-Antwi K, Sabin L, Singh N, MacLeod WB. Impact of biomass fuels on pregnancy outcomes in central East India. Environ Health. 2014; 13(1): 1.

186	Wylie BJ, Kishashu Y, Matechi E, Zhou Z, Coull B, Abioye AI, Dionisio KL, Mugusi F, Premji Z, Fawzi W, Hauser R, Ezzati M. Maternal exposure to carbon monoxide and fine particulate matter during pregnancy in an urban Tanzanian cohort. Indoor Air. 2017; 27(1): 136-146.
187	Xiao Q, Chen H, Strickland MJ, Kan H, Chang HH, Klein M, Yang C, Meng X, Liu Y. Associations between birth outcomes and maternal PM2.5 exposure in Shanghai: A comparison of three exposure assessment approaches. Environ Int. 2018; 117: 226- 236.
188	Ye L, Ji Y, Lv W, Zhu Y, Lu C, Xu B, Xia Y. Associations between maternal exposure to ai r pollution and birth outcomes: a retrospective cohort study in Taizhou, China. Enviro n Sci Pollut Res Int. 2018; 25(22): 21927-21936.
189	Yin P, Brauer M, Cohen A, Burnett RT, Liu J, Liu Y, Liang R, Wang W, Qi J, Wang L, Zhou M. Long-term Fine Particulate Matter Exposure and Nonaccidental and Cause- specific Mortality in a Large National Cohort of Chinese Men [Unpublished]. Environ Health Perspect. 2017; 125(11): 117002.
190	Yin P, Brauer M, Cohen A, Burnett RT, Liu J, Liu Y, Liang R, Wang W, Qi J, Wang L, Zhou M. Long-term Fine Particulate Matter Exposure and Nonaccidental and Cause- specific Mortality in a Large National Cohort of Chinese Men. Environ Health Perspect. 2017; 125(11): 117002.
191	Yu K, Qiu G, Chan K-H, Lam K-BH, Kurmi OP, Bennett DA, Yu C, Pan A, Lv J, Guo Y, Bian Z, Yang L, Chen Y, Hu FB, Chen Z, Li L, Wu T. Association of Solid Fuel Use With Risk of Cardiovascular and All-Cause Mortality in Rural China. JAMA. 2018; 319(13): 1351– 61.
192	Yuan L, Zhang Y, Wang W, Chen R, Liu Y, Liu C, Kan H, Gao Y, Tian Y, Shanghai Birth Cohort Study. Critical windows for maternal fine particulate matter exposure and adverse birth outcomes: The Shanghai birth cohort study. Chemosphere. 2020; 240: 124904.
193	Yucra S, Tapia V, Steenland K, Naeher LP, Gonzales GF. Association between biofuel exposure and adverse birth outcomes at high altitudes in Peru: a matched case- control study. Int J Occup Environ Health. 2011; 17(4): 307-13.

Section 5.4: Risk-outcome modelling

To estimate relative risk curves for each of the $PM_{2.5}$ outcomes, we used the MR-BRT meta-regression tool to fit splines on the input datasets of OAP and HAP studies. We used the following functional form, where X and X_{CF} represent the range of exposure characterised by the effect size:

$$log\left(\frac{MRBRT(X)}{MRBRT(X_{cf})}\right) \sim \log(Published\ Effect\ Size)$$

Several key updates were made to the model fitting methods. For each risk–outcome pair, model settings and priors were tested when fitting the MR-BRT splines. The final models used third-order splines with three interior knots and a constraint on the right-most segment forcing the fit to be linear rather than cubic. This regularized, linear extrapolation allowed us to extrapolate to higher $PM_{2.5}$ concentrations without the instability often present in higher-order splines. Splines were also constrained to be concave and monotonically increasing, the most biologically plausible shape for the $PM_{2.5}$ risk curve. We used an ensemble approach to generate final spline predictions, in which 50 different models were run with randomly placed knots, then weighted and combined based on a measure of fit that penalises excessive changes in the maximum derivative of the curve. Knots were free to be placed across the entire domain of the input exposure data. To prevent over-fitting, on the non-linear segments, we implemented a Gaussian prior on the third derivative of mean 0 and variance $1*10^{-4}$. On the linear segment, a stronger prior of mean 0 and variance $1*10^{-6}$ was used to ensure that the risk

curves do not continue to increase beyond the range of the data. 10% of all observations were trimmed during model fitting, in accordance with GBD protocol across Risk Factor teams.

To select significant covariates from those extracted to quantify between-study heterogeneity, we performed covariate selection (see table S7 below). The MR-BRT automated covariate selection tool implements a two-step process. First, a series of loosening Lasso penalty parameters are applied to a log-linear meta-regression on all input effect size observations. Then, covariates with a non-zero coefficient are tested for significance using a Gaussian prior (significance threshold = 0.05). A Gaussian prior was used on each covariate's beta during spline fitting with a mean 0 and variance of 0.1 multiplied by the standard deviation of the beta from the log-linear meta-regression. Type 2 diabetes was the only outcome for which a significant covariate was identified. Its selected covariate was cv_hap, a binary covariate indicating whether an observation was from a household air pollution study.

Table S7: Significant covariates used in the MR-BRT splines

**Covariate name**	**Covariate description**
cv_subpopulation	Study represents the general population; study represents a subgroup (eg, high-risk group)
cv_exposure_population	Study measures individual-level exposure (≤1 km radius); study measures population-level exposure
cv_exposure_self_report	Exposure is self-reported; exposure is measured externally
cv_exposure_study	Exposure is measured multiple times; exposure is measured only at baseline
cv_outcome_self_report	Outcome is self-reported; outcomes is based on death certificate or medical record
cv_outcome_unblinded	Study implements unblinded assessment; assessment of outcome is blind to exposure (and vice versa)
cv_reverse_causation	Study presents no risk of reverse causation; risk of reverse causation
cv_confounding_nonrandom	Non-randomised study; randomised study
cv_confounding_uncontrolled	Study is randomised/outcome controlled for age, sex, education, income, and all critical determinants of outcome; study is controlled for age, sex, and other critical determinants of outcome; study is controlled for only age and sex
cv_selection_bias	Study reports >95% follow-up; study reports 85–95% follow-up; study reports <85% follow-up
cv_hap	Studies household air pollution; studies ambient air pollution

To calculate burden estimates, we produced 1000 predictions of effect size across the exposure distribution. The mean relative risk is found by taking the mean of these predictions, and the 95% UI is found by extracting the 2.5th and 97.5th percentiles of the predictions. These predictions allowed us to characterize the uncertainty of the model by examining between-study heterogeneity. We implemented the Fisher scoring correction to the heterogeneity parameter, which corrects for data-sparse situations. In such cases, the between- study heterogeneity parameter estimate may be 0, simply from lack of data.

The Fisher scoring correction uses a quantile of gamma, which is sensitive to the number of studies, study design, and reported uncertainty.

Section 5.5: Risk-outcome scoring

Section 5.5.1: Overview

Risk-outcome scores provide an empirical measure of the strength of evidence for risk-outcome pairs across risk factors in the GBD and are therefore useful for standardised comparison. Risk-outcome scores evaluate the area between the lower bound of the 95% uncertainty interval and the x-axis for harmful risk factors, including $PM_{2.5}$ pollution.

Section 5.5.2: Main approach

Prior to generating a risk-outcome score, we conducted an additional post-analysis step to detect and flag publication bias in the input data (table S8). This approach is based on the classic Egger's regression strategy, which is applied to the residuals in our model. In the current implementation, we do not correct for publication bias but flag the risk–outcome pairs where the risk for publication bias is significant. Of the $PM_{2.5}$ outcomes, three were flagged for publication bias: birthweight, ischaemic heart disease, and type 2 diabetes.

Table S8: Residual analysis coefficients for publication bias detection for all outcomes (excluding adverse reproductive outcomes).

Outcome	Egger p-value	Egger mean	Egger SD	Publication bias
Birthweight	0.0208	–0.322	0.158	X
Gestational age	0.249	–0.130	0.192	
Ischaemic heart disease	0.0164	0.322	0.151	X
Stroke	0.0717	0.186	0.127	
LRI	0.178	0.102	0.110	
Trachea, bronchus, and lung cancers	0.191	0.108	0.123	
COPD	0.423	0.0359	0.186	
Type 2 diabetes	0.0419	0.408	0.236	X

To calculate the risk-outcome score, we generated an uncertainty interval from 1000 simulations of the adjusted summary effect size (retaining uncertainty information from between-study heterogeneity predictions and the Fisher information correction). We then evaluated the risk-outcome score between the 15th and 85th percentiles of the input data exposure distribution. Risk-outcome scores and star

ratings are below (table S9). Risk-outcome scores are not reported for birthweight and gestational age because these are mediated outcomes.

Table S9: Risk-outcome scores and star ratings for all outcomes (excluding adverse reproductive outcomes).

Outcome	Risk-outcome score	Star rating
Ischaemic heart disease	0.259	3
Stroke	0.167	3
LRI	0.126	2
Trachea, bronchus, and lung cancers	0.342	3
COPD	0.441	4
Type 2 diabetes	0.188	3

Section 5.5.3: Risk-outcome curves

The following figures display risk curves for each outcome (figures S5-S10). The dashed line depicts the GBD 2017 IER including active smoking data, the dotted line depicts the GBD 2019 MR-BRT curve without active smoking but with secondhand smoking data, and the solid line depicts the GBD 2021 MR-BRT curve without the inclusion of active smoking or secondhand smoking data. For GBD 2021, a single curve is used for cardiovascular diseases (ischaemic heart disease, stroke) for all ages, so only one plot is displayed for each of these outcomes. For the GBD 2017 and GBD 2021 curves, the curve for the age group 60–64 is plotted for the cardiovascular disease outcomes because these cycles used age-specific cardiovascular disease curves. For birthweight and gestational age, no curve is displayed for GBD 2017 because these outcomes were added to the GBD in the 2019 cycle. The grey shaded areas represent the 95% uncertainty interval. The red box represents the TMREL area of the curve. On each page, the first figure depicts the typical range of outdoor exposure, whereas the second plot includes higher levels typical of household air pollution exposure.

Each point or number represents one study effect size (study numbers given in table S6). These sources are also available in appendix 2 pp 6. Each is plotted at the 95_{th} percentile of the exposure distribution (OAP) or the expected level of exposure for individual using solid fuel (HAP). The relative risk is plotted relative to the predicted relative risk at the 5_{th} percentile of exposure distribution (OAP) or the expected (ambient only) level of exposure for individuals not using solid fuel (HAP). For example, a study predicting a relative risk of 1.5 for an exposure range of 10 to 20 would be plotted at (20, MR--BRT(10)*1.5). Arrows represent studies that would have been outside the range of the plot but have been shifted to be included in the figure.

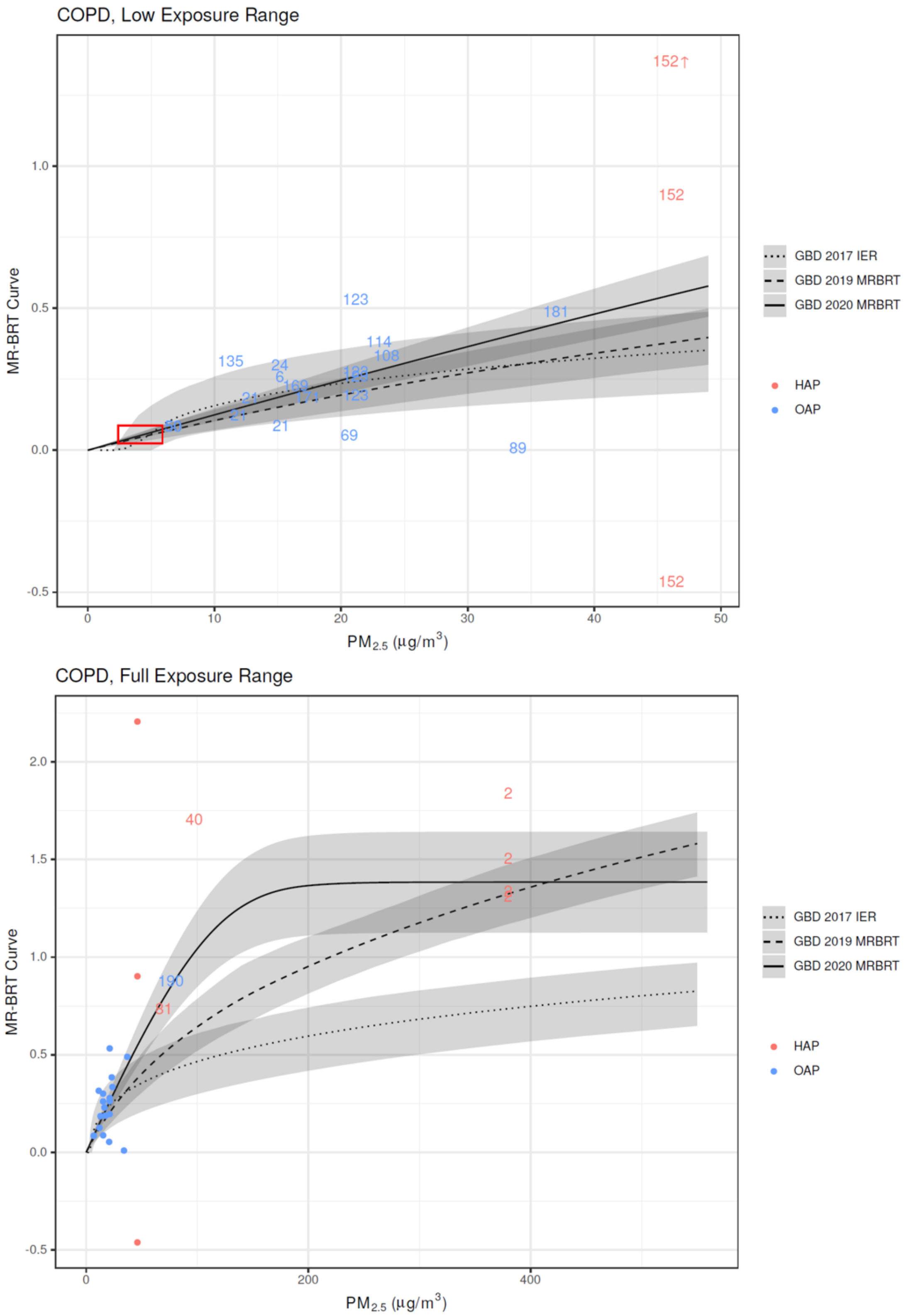

Figure S5: Relative risk curves for chronic obstructive pulmonary disease (COPD) for low exposure (top) and the full exposure range (bottom); the GBD 2017 IER curve, the GBD 2019 MR-BRT curve, and the GBD 2021 MR-BRT curve are shown.

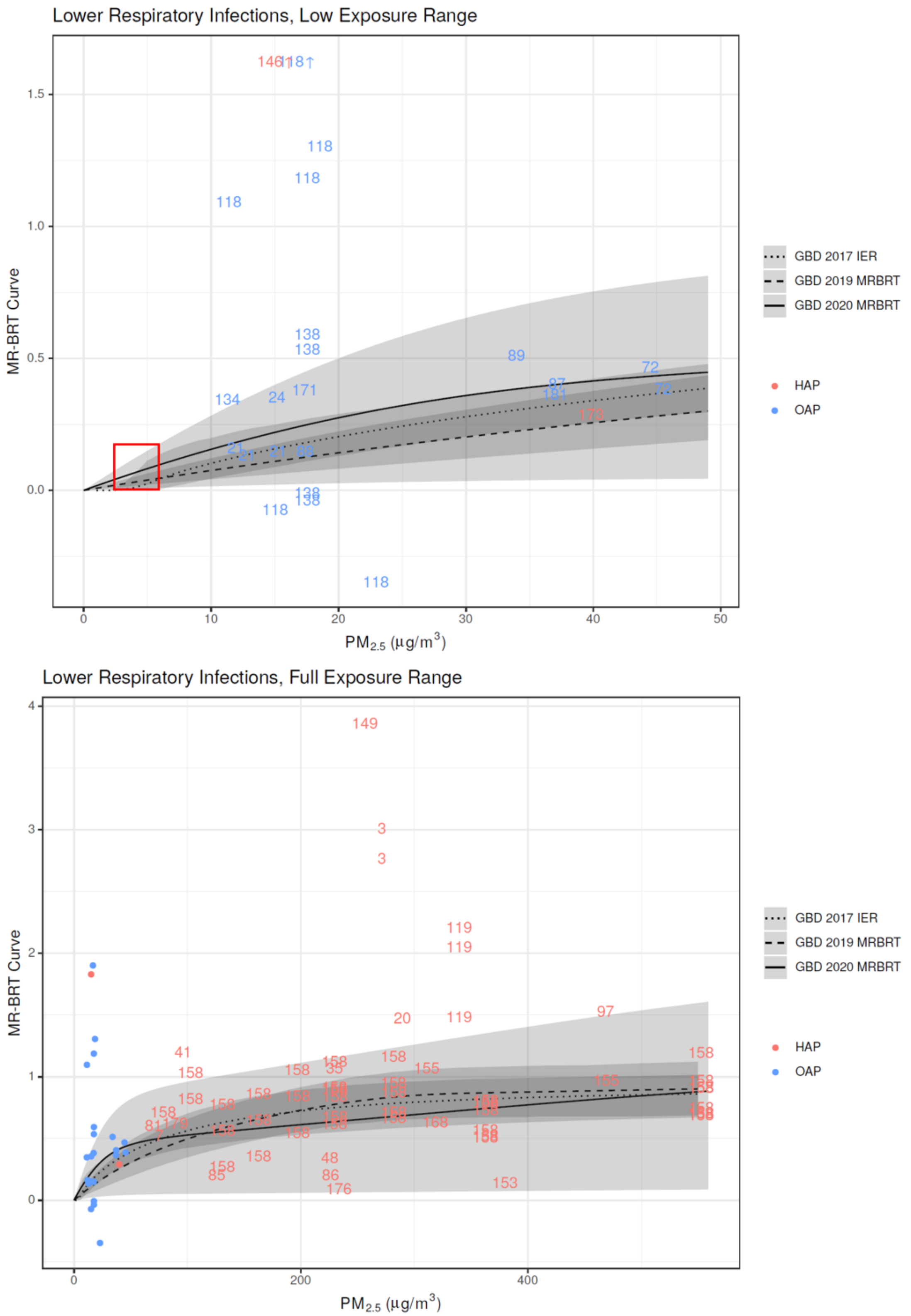

Figure S6: Relative risk curves for lower respiratory infections for low exposure (top) and the full exposure range (bottom); the GBD 2017 IER curve, the GBD 2019 MR-BRT curve, and the GBD 2021 MR-BRT curve are shown.

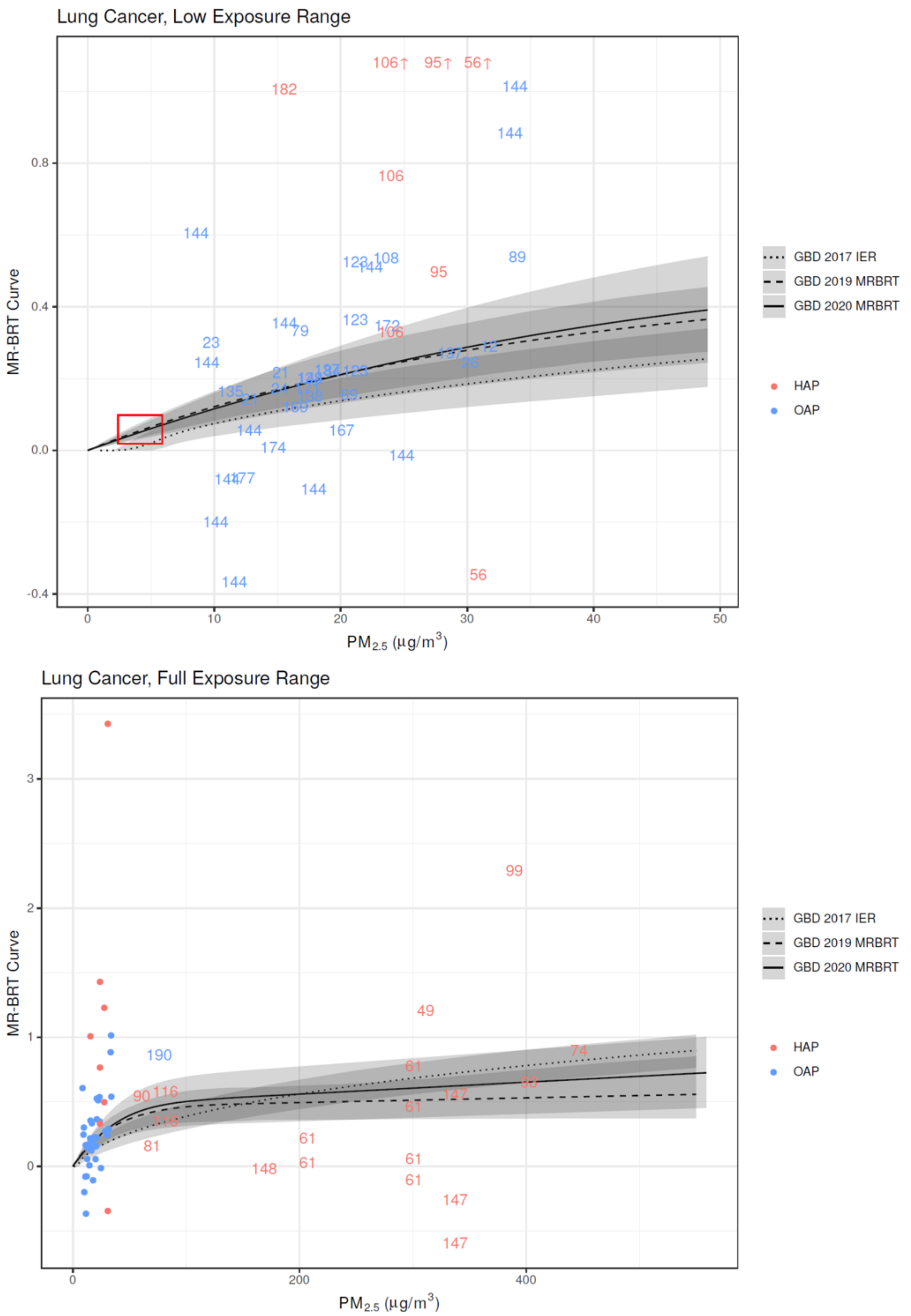

Figure S7: Relative risk curves for trachea, bronchus, and lung cancers for low exposure (top) and the full exposure range (bottom); the GBD 2017 IER curve, the GBD 2019 MR-BRT curve, and the GBD 2021 MR-BRT curve are shown.

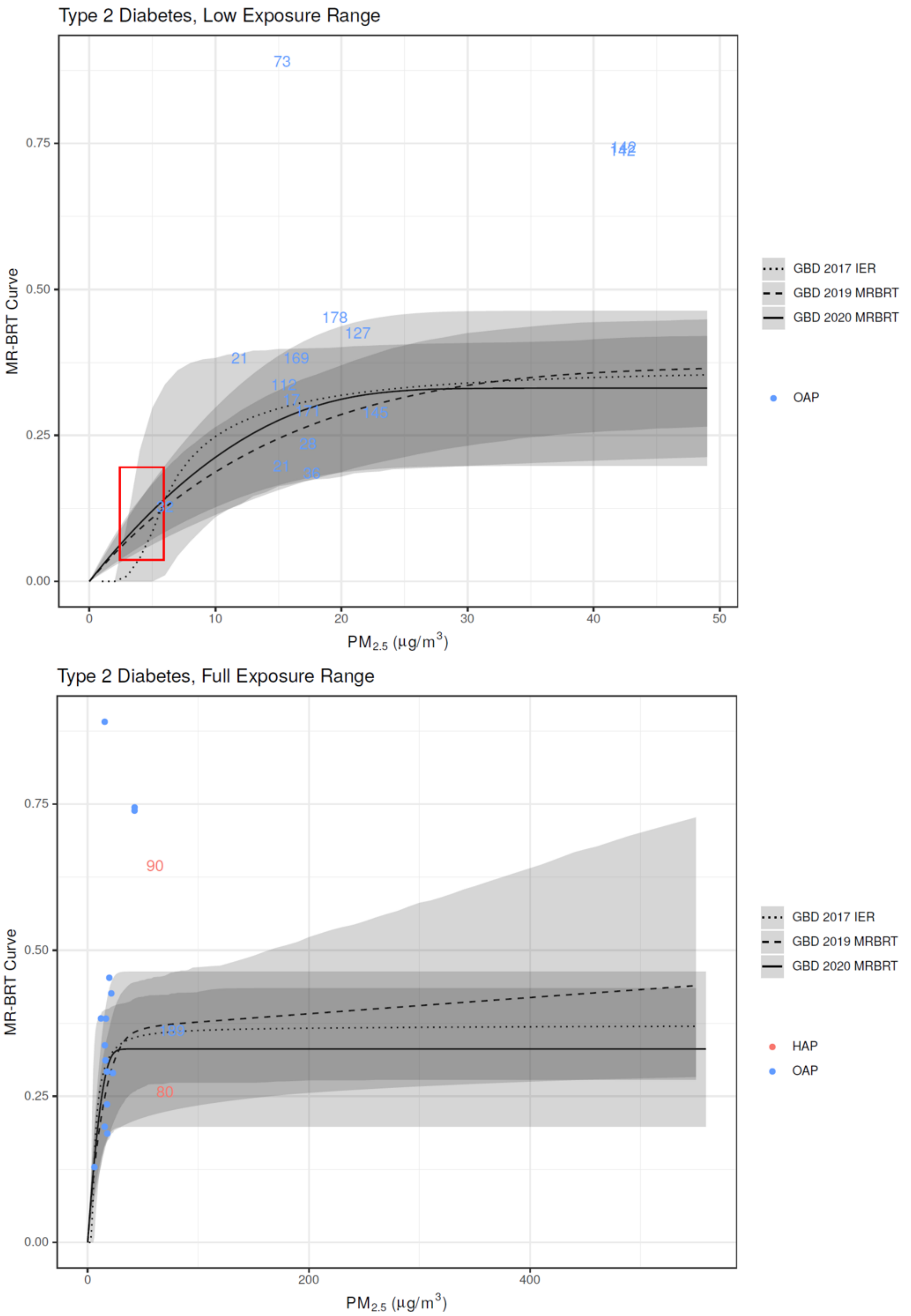

Figure S8: Relative risk curves for type 2 diabetes for low exposure (top) and the full exposure range (bottom); the GBD 2017 IER curve, the GBD 2019 MR-BRT curve, and the GBD 2021 MR-BRT curve are shown.

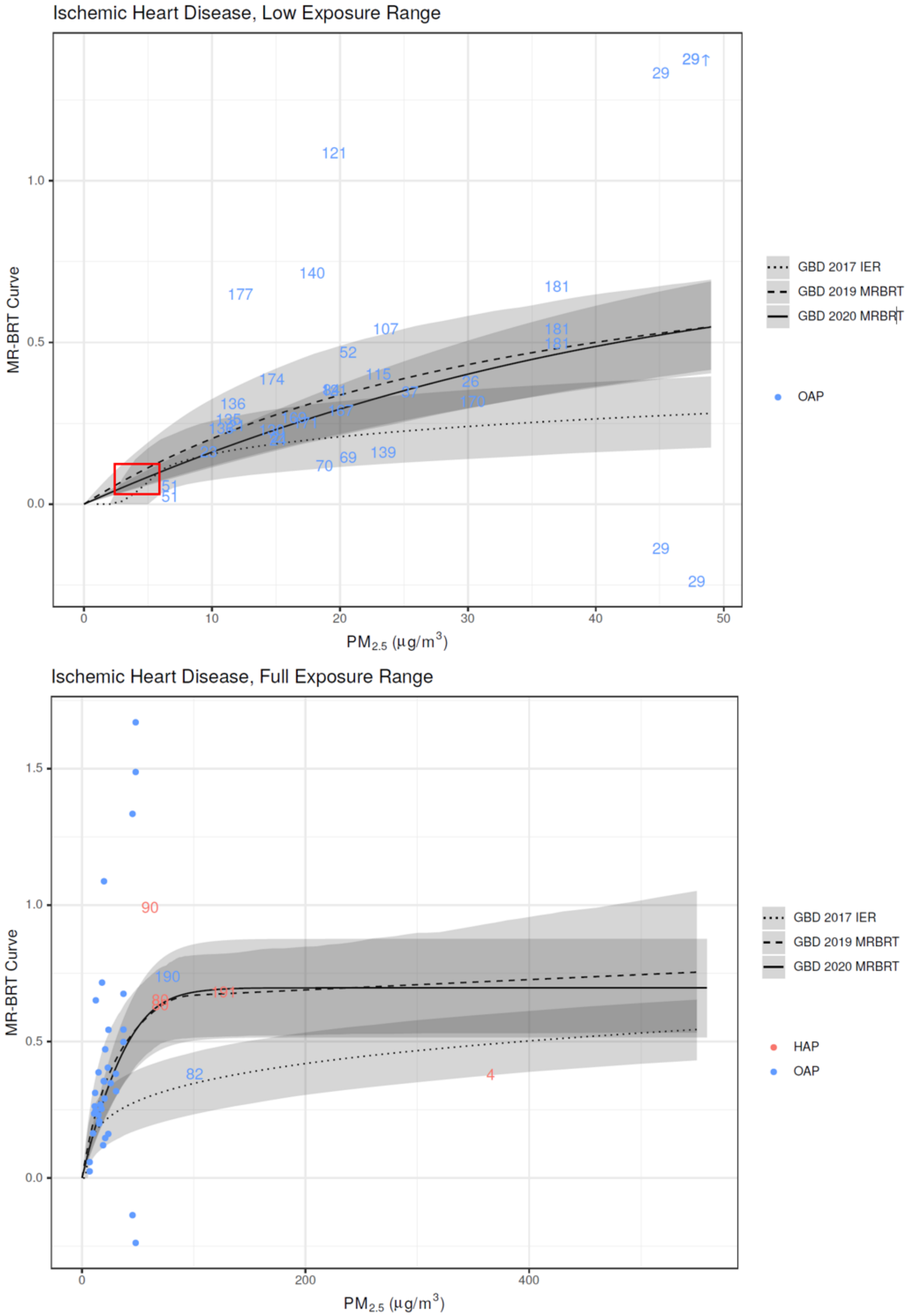

Figure S9: Relative risk curves for ischemic heart disease for low exposure (top) and the full exposure range (bottom); the GBD 2017 IER curve, the GBD 2019 MR-BRT curve, and the GBD 2021 MR-BRT curve are shown.

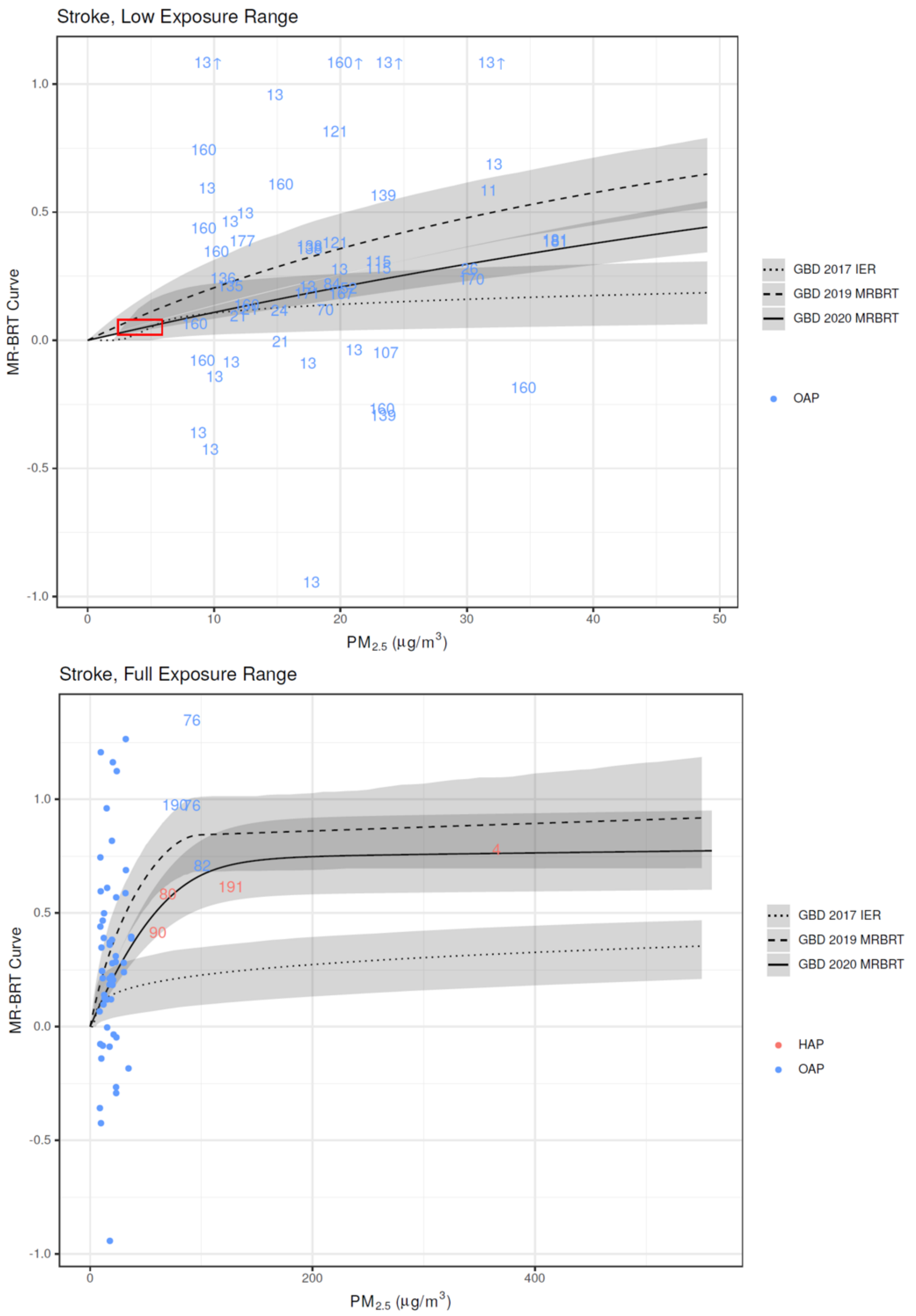

Figure S10: Relative risk curves for stroke for low exposure (top) and the full exposure range (bottom); the GBD 2017 IER curve, the GBD 2019 MR-BRT curve, and the GBD 2021 MR-BRT curve are shown.

Section 5.6: Cataract

To calculate location-specific relative risk for cataract, we utilized the meta-regression of cataract and household air pollution we performed for GBD 2019.[2] Previously, we had used an external meta-analysis which gave a summary effect of 2.47 (95% CI 1.63-3.73).[10] To create our meta-regression analysis, we extracted all sources utilized in the above meta-analysis apart from one cross-sectional study, as it is standard practice for GBD risk factor analyses to exclude cross-sectional analyses; they are considered to be a lower level of evidence.[9] We identified one additional study that analyzed cataract and various fuel types in a literature search,[25] but it did not contain a comparison group without solid fuel use and was excluded. The resulting dataset comprised six sources with eight estimates from India and Nepal.

Our MR-BRT meta-regression that yielded a summary effect size of 2.56 (1.68-3.59) (figure S11). The only covariate included was whether study participants were blind to the exposure-outcome pair of interest, and a Gaussian distribution with mean 0 and variance 0.1 was used as a prior. The prior for gamma was a Gaussian distribution with mean 0.04 and variance 0.1. The studies included, reported effect size for males, females, and/or both sexes. For GBD 2019, we conducted a sensitivity analysis by including a covariate for sex but found no significant difference between the sexes in effect size. Thus, we estimated cataract as an outcome of household air pollution for both males and females in GBD 2019 and GBD 2021.

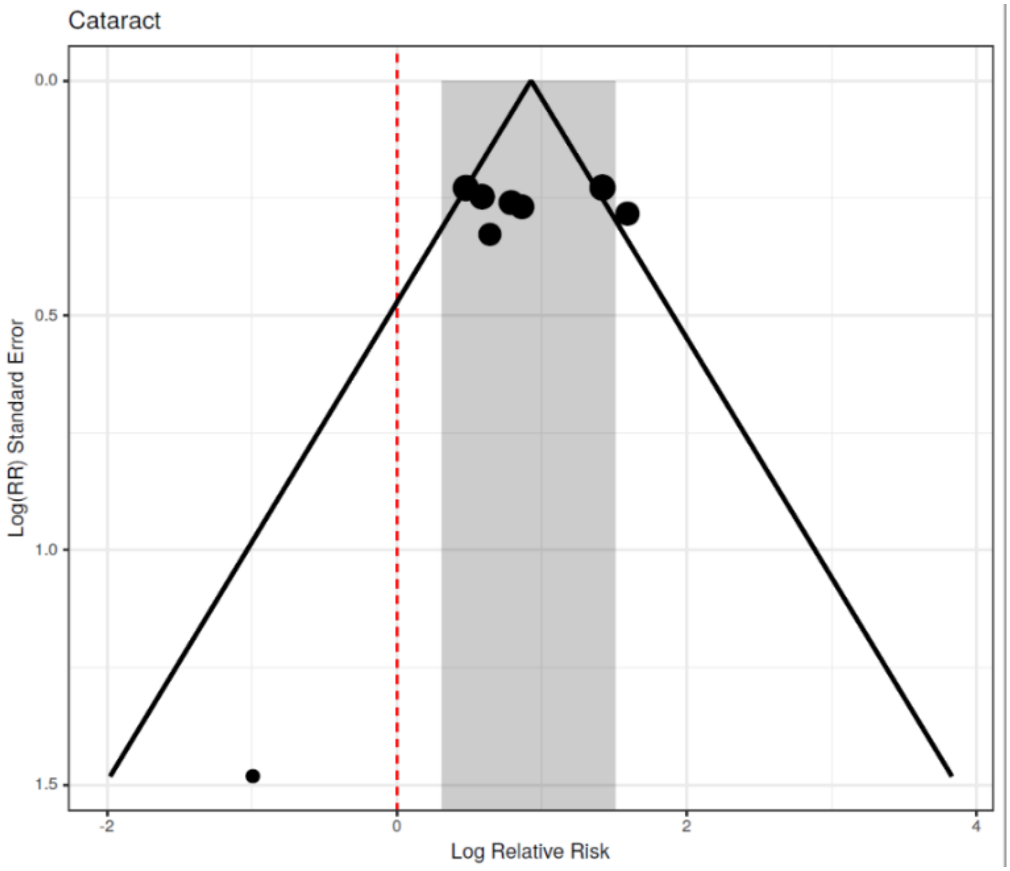

Figure S11. Funnel plot showing estimates of relative risk for cataract, 2021.

Section 5.7: Low birthweight and short gestation mediation analysis

In GBD 2021, as in GBD 2019, low birthweight and short gestation were included as $PM_{2.5}$ outcomes via a mediation analysis. Low birthweight and short gestation include mortality due to diarrhoeal diseases, lower respiratory infections, upper respiratory infections, otitis media, meningitis, encephalitis, neonatal preterm birth, neonatal encephalopathy due to birth asphyxia and trauma, neonatal sepsis and other neonatal infections, haemolytic disease and other neonatal jaundice, and other neonatal disorders. Morbidity estimates were also calculated for neonatal preterm birth. These outcomes are specific to the neonatal ages: 0–6 days and 7–27 days.

The following is a summary of methods used to conduct the mediation analysis. For GBD 2019, we conducted a systematic review of all cohort, case-control, or randomised-controlled trial studies of ambient $PM_{2.5}$ pollution or household air pollution and birthweight or gestational age outcomes.[2] Outcomes measured included continuous birthweight (bw), continuous gestational age (ga), low birthweight (LBW) (<2500 g), preterm birth (PTB) (<37 weeks), and very preterm birth (VPTB) (<32 weeks). This systematic review was then updated for a paper lead by our collaborator, Dr. Rakesh Gosh, and included any papers published until April 4, 2021.[16]

We acknowledge that several new papers, most notably results from the HAPIN trial, have been published.[26-29] We are screening these sources for inclusion in future rounds of the GBD.

Birthweight and gestational age are modelled using a continuous joint distribution for the GBD. To determine how these distributions are influenced by $PM_{2.5}$ pollution, we used available literature to model the continuous shift in birthweight (bw, grams) and gestational age (ga, weeks) at a given $PM_{2.5}$ exposure level.

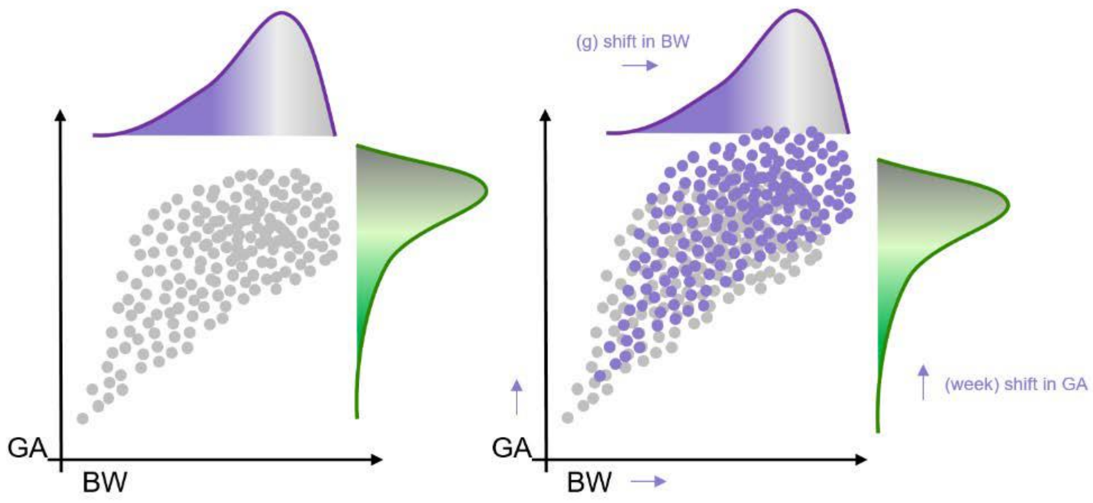

Figure S12: Joint distribution for birthweight and gestational age (left) with an example shift of birthweight (in grams) and gestational age (in weeks) (right).

When available, we used estimates of continuous shifts in bw or ga directly from each study. When shifts were not available, we converted the published OR/RR/HR for LBW, PTB, or VPTB using the following strategy:

1. Extract the OR/RR/HR from the study.
2. Select the GBD 2017 estimated bw-ga joint distribution for the study location and year.
3. Calculate the number of grams or weeks required to shift the distribution such that the proportion of births under the specified threshold (P) is reduced by the study effect size to a counterfactual level (P_{cf}).
4. Save the resulting shift and 95% CI as the continuous effect.

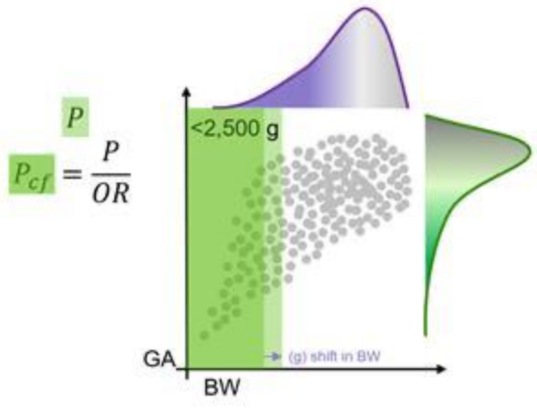

Figure S13: Example of a shift calculated from an OR.

When preparing HAP data to fit splines, we used the same strategy described above for other outcomes to map HAP input data to $PM_{2.5}$ exposure values. We then fit MR-BRT splines to the input studies, where the difference in the value of the model at the upper concentration (X) and the value of the model at the counterfactual concentration (X_{CF}) is equal to the published or calculated shift in bw or ga:

$$MRBRT(X) - MRBRT(X_{CF}) \sim Shift$$

We used the same model fitting process, settings, and covariate selection process as described above for the other outcomes. The only exception is that, because the change in birthweight and gestational age was expected to be negative, the splines were constrained to be monotonically decreasing.

Figures S14 and S15 (below) display MR-BRT curves for linear shift in grams (bw) and weeks (ga) .

We used the curves of estimated shifts across the exposure range to predict the shift in both birthweight and gestational age for total female particulate matter pollution exposure in each location and year. Because the epidemiological studies mutually controlled for birthweight and gestational age, we assumed these shifts are independent. We then shifted the observed distributions to reflect the expected bwga distribution in the absence of particulate matter pollution. These shifted distributions were used as the counterfactual in the PAF calculation equation to calculate the burden attributable to $PM_{2.5}$ pollution.

To calculate PAFs, the distribution is divided into 56 bw-ga categories, each with a unique RR.

Let p_i be the observed proportion of infants in category i and p_i' be the counterfactual proportion of infants in category i if there were no particulate matter pollution. Then,

$$PAF_{PM} = \frac{\sum_{i \epsilon bwga\ category} RR_i\, p_i - \sum_{i \epsilon bwga\ category} RR_i p_i{}'}{\sum_{i \epsilon bwga} RR_i p_i}$$

We proportionately split this PAF to ambient and HAP based on exposure as described below. One important assumption to note is that we assume the shift in bw and ga is linear across the bwga distribution.

For lower respiratory infections, $PM_{2.5}$ -attributable PAFs are directly estimated in addition to estimated through bwga mediation. We expect that some of the directly estimated PAFs are mediated through bw and ga. Additionally, the directly estimated PAF is based on a summary of relative risks for all children under 5 years, so there is a possibility that the mediated PAF, which is more finely resolved, could be greater. To avoid double counting, for the two neonatal age groups (0–6 days and 0–27 days), we take the maximum of the two PAF estimates. If the directly estimated PAF is greater than the bwga-mediated PAF, we take the direct estimate, and if the mediated PAF is greater, we take the mediated estimate.

PTB incidence and mortality are both outcomes measured in the GBD. 100% of the burden for this cause is attributable to short gestation. To calculate the percentage attributable to particulate matter pollution, we estimated the percentage of newborns born at less than 37 weeks (pptb) and the percentage of newbornsthat would have been born at less than 37 weeks in the counterfactual scenario of no particulate matter pollution (p_{ptb} ′).

$$PAF_{ptb,pm} = 1 - \frac{p_{ptb}{}'}{p_{ptb}}$$

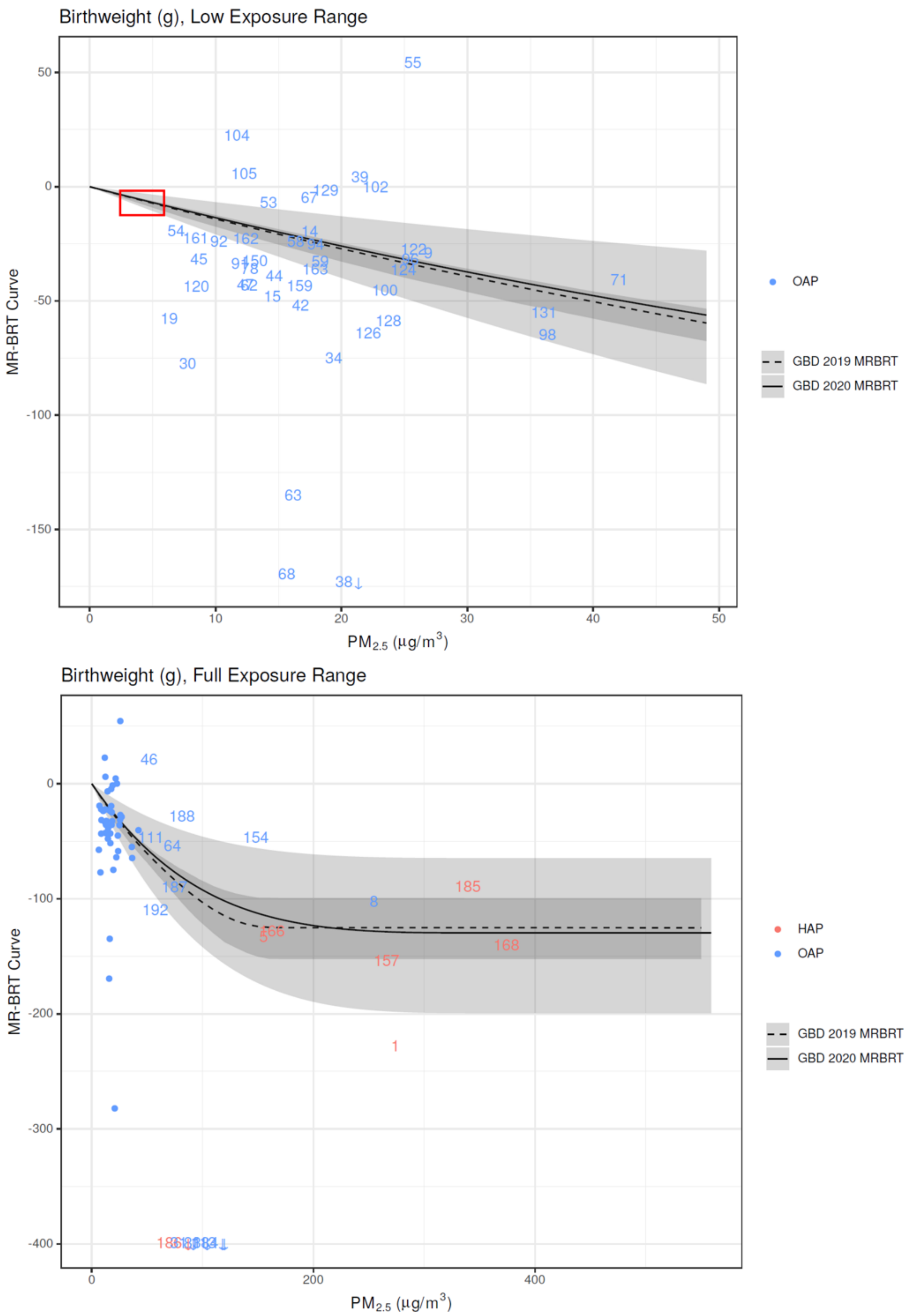

Figure S14: Relative risk curves for low birthweight for low exposure (top) and the full exposure range (bottom); the GBD 2017 IER curve, the GBD 2019 MR-BRT **curve**, and the GBD 2021 MR-BRT curve are shown.

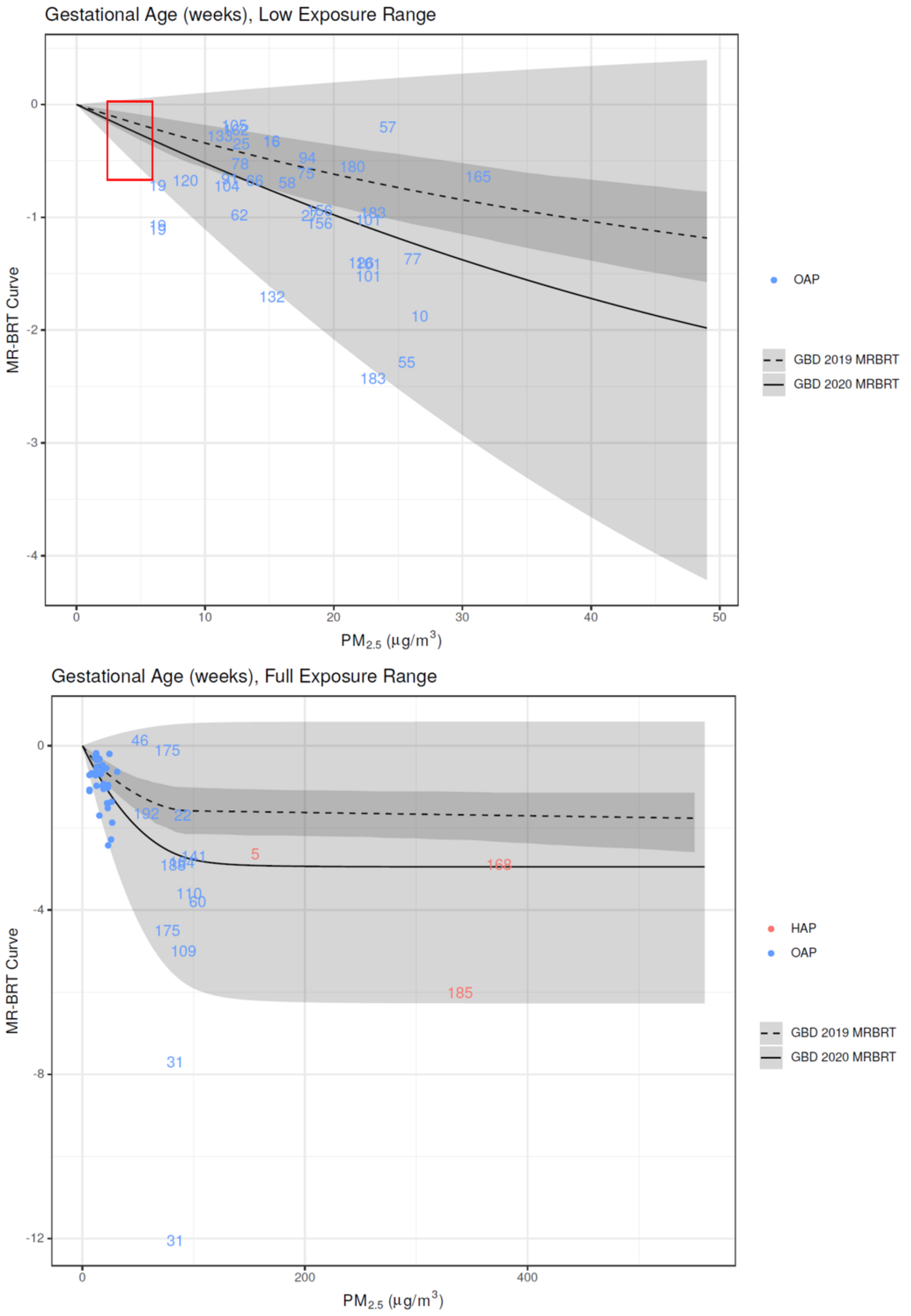

Figure S15: Relative risk curves for gestational age for low exposure (top) and the full exposure range (bottom); the GBD 2017 IER curve, the GBD 2019 MR-BRT **curve**, and the GBD 2021 MR-BRT curve are shown.

Section 6: Proportional population attributable fraction

The population attributable fraction (PAF) is the proportion of burden that would be eliminated if exposure to a particular risk factor were reduced to "ideal exposure scenario".[1] Please note that the formulas presented below are for the attributable fraction; because we assume 100% of the population is exposed, the population attributable fraction reduces to the attributable fraction. For further details on GBD PAF methodology, please see citation appendix 1 section 2.5.[1] For further details on our ambient air pollution estimation methodology, please see citation appendix 1 pp XX.[1]

Prior to GBD 2017, relative risks for both ambient and HAP exposures were obtained from the risk curve as a function of exposure, relative to the same TMREL. Were a country or territory to reduce only one of these risk factors, the other would remain. We did not consider the joint effects of particulate matter from outdoor exposure and burning solid fuels for cooking. For GBD 2017, we developed a new approach to use the risk curve for obtaining PAFs for both OAP and HAP, which was also implemented in GBD 2019 and 2021.

Let Exp_{OAP} be the ambient $PM_{2.5}$ exposure level and Exp_{HAP} be the excess exposure for those who use solid fuel for cooking. Let P_{HAP} be the proportion of the population using solid fuel for cooking. We calculated PAFs at each 0.1º × 0.1º grid cell. We assumed that the distribution of persons using solid fuel for cooking (HAP) was equivalent across all grid cells of the GBD location.

For the proportion of the population not exposed to HAP the relative risk was:

$$RR_{OAP} = \frac{MRBRT(z = Exp_{OAP})}{MRBRT(z = TMREL)}$$

And for those exposed to HAP, the relative risk was:

$$RR_{HAP} = \frac{MRBRT(z = Exp_{HAP} + Exp_{OAP})}{MRBRT(z = TMREL)}$$

We then calculate a population-level RR and PAF for all particulate matter exposure:

$$RR_{PM} = RR_{OAP}(1 - P_{HAP}) + RR_{HAP} * P_{HAP}$$

$$PAF_{PM} = \frac{RR_{PM} - 1}{RR_{PM}}$$

We population weight the grid-cell level particulate matter PAFs to get a country-level PAF, and finally, we split this PAF based on the average exposure to each OAP and HAP:

$$PAF_{OAP} = \frac{Exp_{OAP}}{Exp_{OAP} + P_{HAP} * Exp_{HAP}} * PAF_{PM}$$

$$PAF_{HAP} = \frac{P_{HAP} * Exp_{HAP}}{Exp_{OAP} + P_{HAP} * Exp_{HAP}} * PAF_{PM}$$

With this strategy, $PAF_{PM} = PAF_{HAP} + PAF_{OAP}$, and no burden is counted twice.

At every step of our PAF modelling pipeline, we generated 500 simulations. The reported point estimate is the mean of these simulations, and we took the 2.5^{th} and the 97.5^{th} percentile of distribution of the simulations to form the confidence interval.

Burden estimates by country and territory and cause are available in appendix 2 pp 7 and at: https://vizhub.healthdata.org/gbd-compare/.

Supplementary Figures S16 to S22

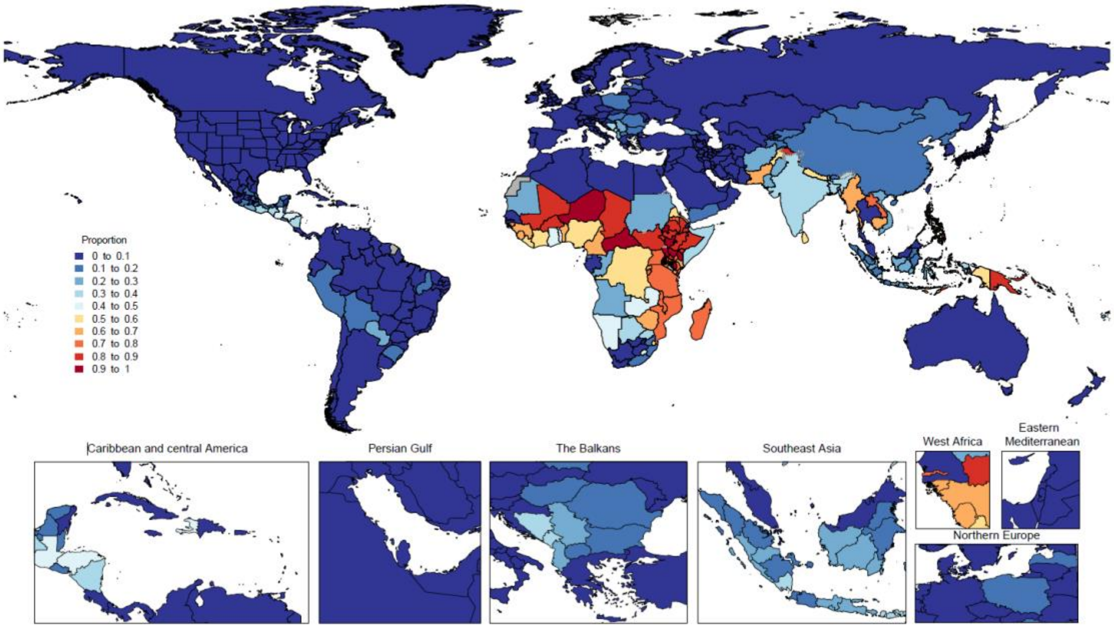

Figure S16. Proportion of population exposed to HAP from wood in 2021.
HAP=household air pollution.

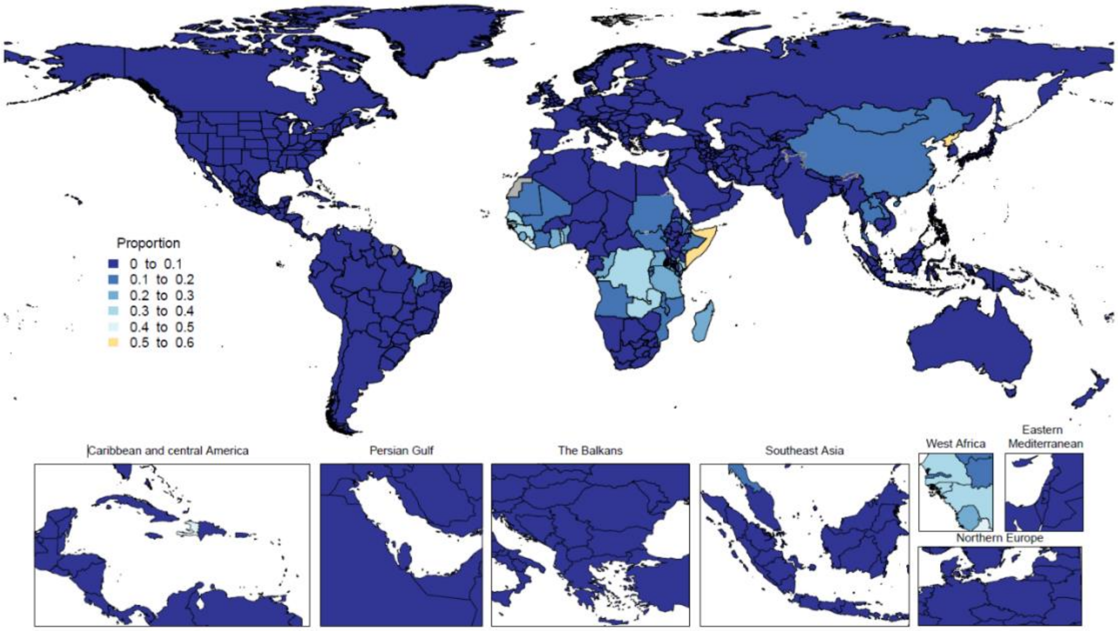

Figure S17. Proportion of population exposed to HAP from coal in 2021.
HAP=household air pollution.

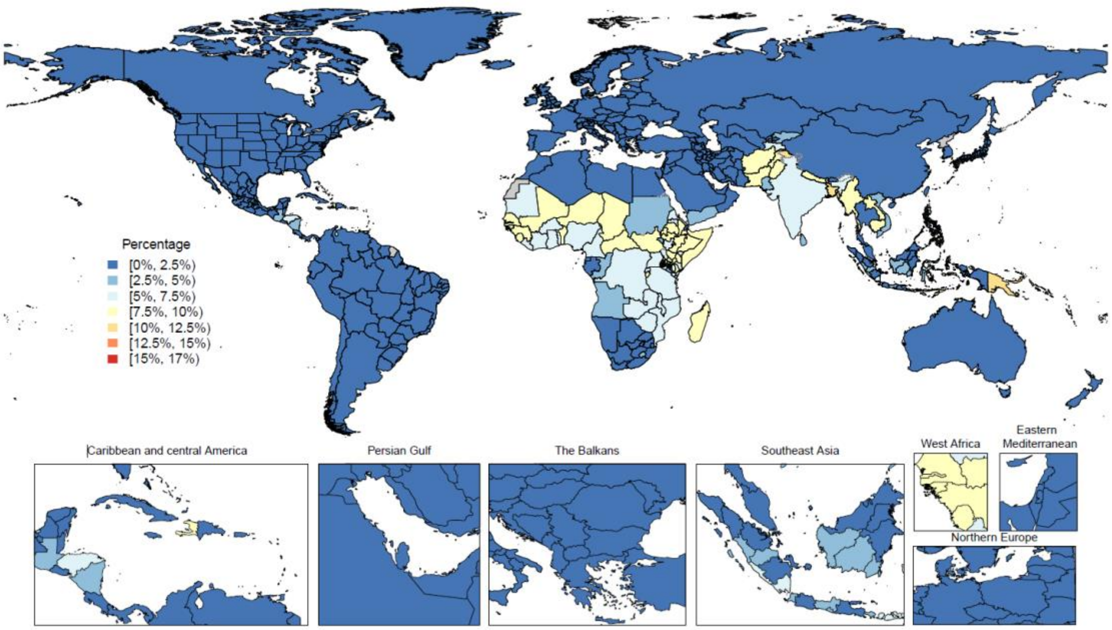

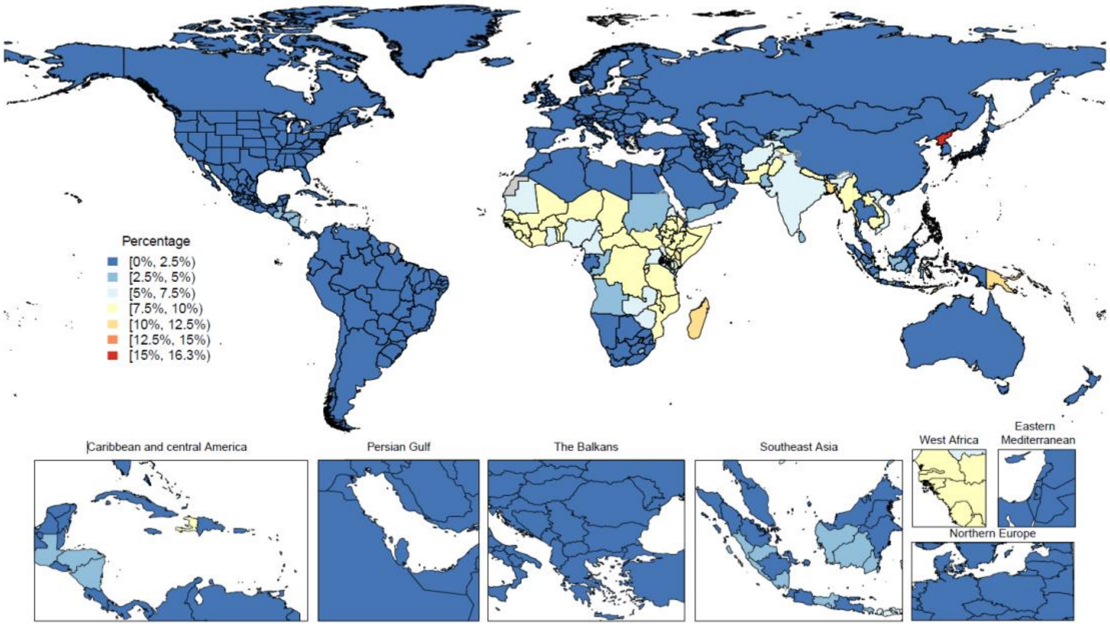

Figure S18. Estimated percentage of all-age DALYs attributable to HAP from solid cooking fuels for females (top) and males (bottom) in 2021.

DALY=disability-adjusted life-years. HAP=household air pollution.

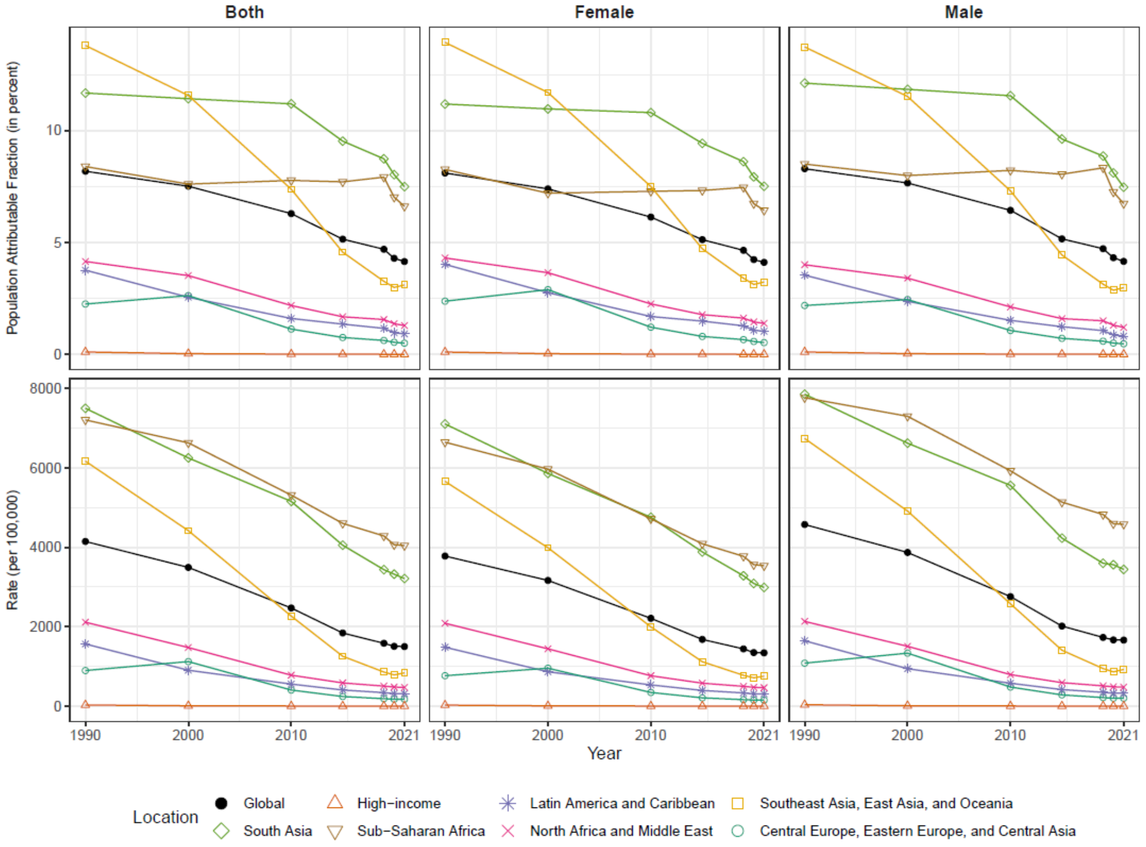

Figure S19. Temporal trends in all-cause burden attributable to HAP from solid cooking fuels in both sexes (left column), females (center column), and males (right column) by PAF (upper) and age-standardized rate of DALYs per 100,000 population (lower), 1990-2021.
DALY=disability-adjusted life-years. HAP=household air pollution. PAF=population attributable fraction.

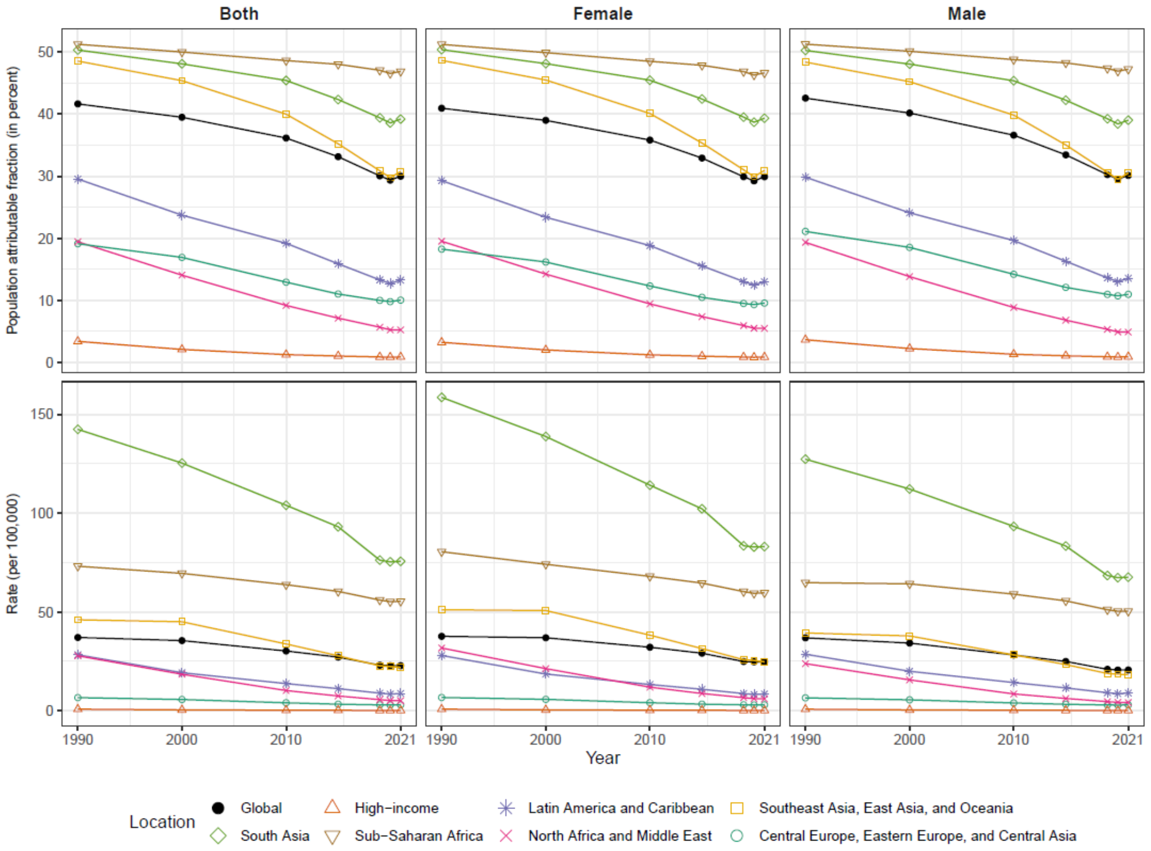

Figure S20. Temporal trends in cataract burden attributable to HAP from solid cooking fuels in both sexes (left column), females (center column), and males (right column) by PAF (upper) and age-standardized rate of DALYs per 100,000 population (lower), 1990-2021.

DALY=disability-adjusted life-years. HAP=household air pollution. PAF=population attributable fraction.

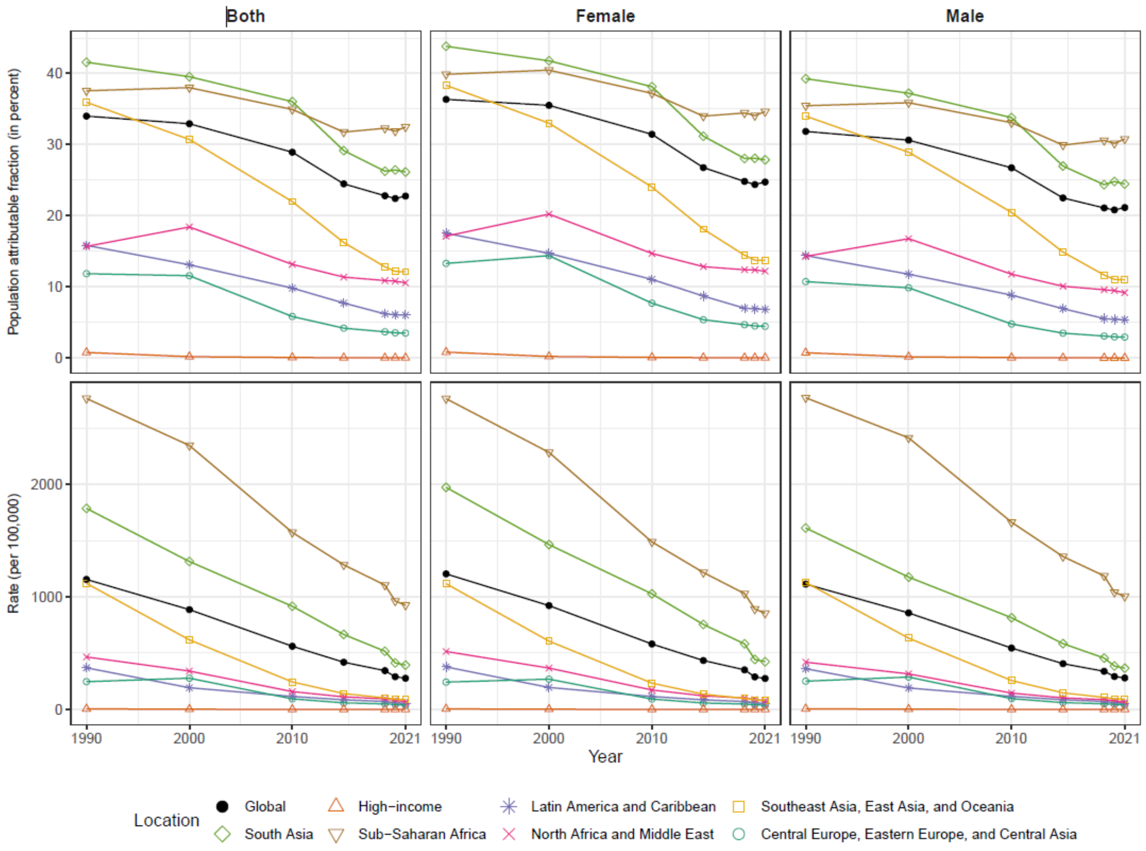

Figure S21. Temporal trends in LRI burden attributable to HAP from solid cooking fuels in both sexes (left column), females (center column), and males (right column) by PAF (upper) and age-standardized rate of DALYs per 100,000 population (lower), 1990-2021.

DALY=disability-adjusted life-years. HAP=household air pollution. LRI=lower respiratory infections. PAF=population attributable fraction.

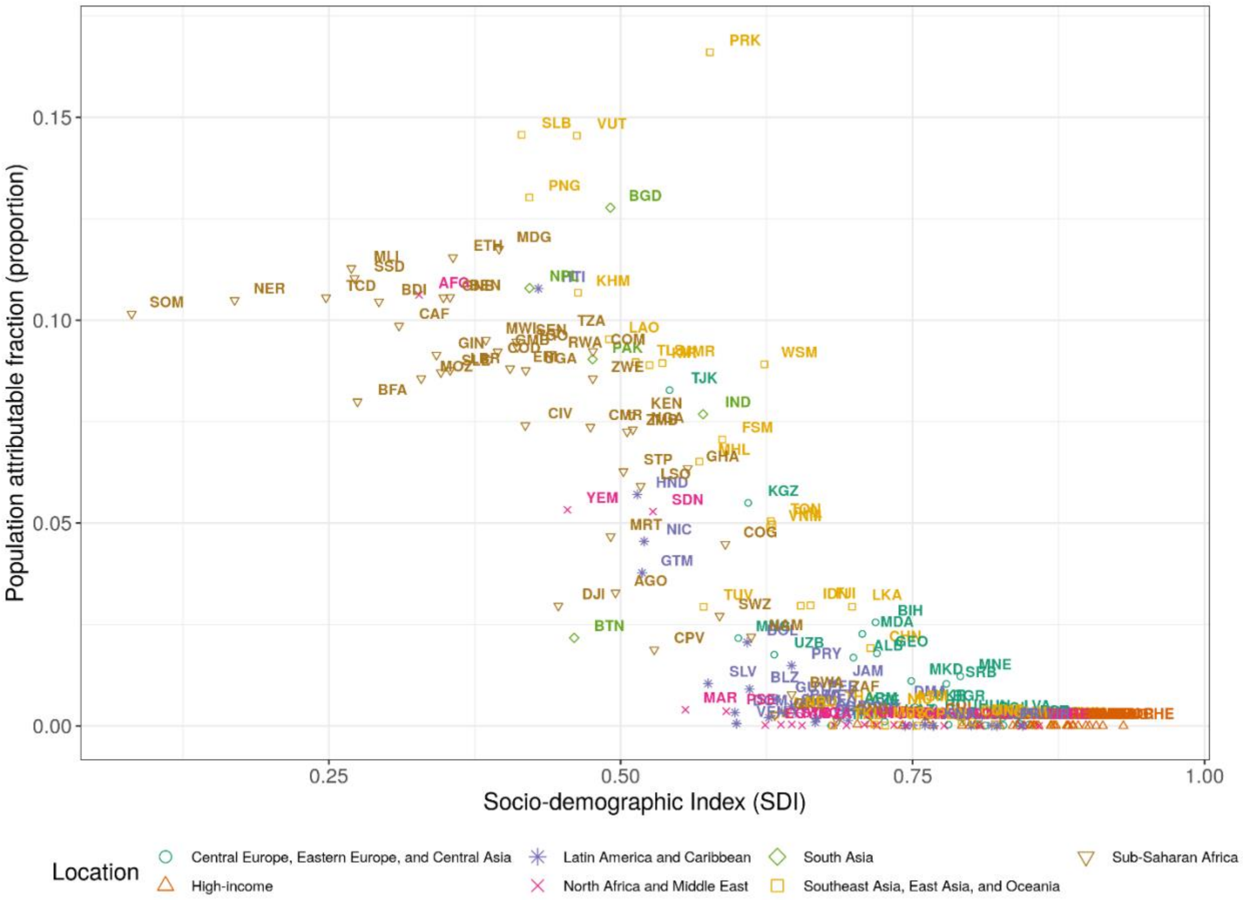

Figure S22. Population attributable fraction of HAP-attributable DALYs vs. SDI, 2021.
DALY=disability-adjusted life-years. HAP=household air pollution. SDI=Socio-demographic index.

References

1 Brauer M, Roth GA, Aravkin AY, *et al.* Global burden and strength of evidence for 88 risk factors in 204 countries and 811 subnational locations, 1990–2021: a systematic analysis for the Global Burden of Disease Study 2021. *The Lancet* 2024; **403**: 2162–203.

2 Murray CJL, Aravkin AY, Zheng P, *et al.* Global burden of 87 risk factors in 204 countries and territories, 1990–2019: a systematic analysis for the Global Burden of Disease Study 2019. *The Lancet* 2020; **396**: 1223–49.

3 WHO. Household energy database. World Health Organization. https://www.who.int/data/gho/data/themes/air-pollution/who-household-energy-db (accessed July 5, 2019).

4 Shupler M, Godwin W, Frostad J, Gustafson P, Arku RE, Brauer M. Global estimation of exposure to fine particulate matter (PM2.5) from household air pollution. *Environment International* 2018; **120**: 354–63.

5 IHME. Global Burden of Disease (GBD): Which locations are studied in the GBD? https://www.healthdata.org/research-analysis/about-gbd (accessed Oct 24, 2024).

6 Zheng P, Afshin A, Biryukov S, *et al.* The Burden of Proof studies: assessing the evidence of risk. *Nat Med* 2022; **28**: 2038–44.

7 Zheng P, Barber R, Sorensen RJD, Murray CJL, Aravkin AY. Trimmed Constrained Mixed Effects Models: Formulations and Algorithms. *Journal of Computational and Graphical Statistics* 2021; **30**: 544–56.

8 Forouzanfar MH, Afshin A, Alexander LT, *et al.* Global, regional, and national comparative risk assessment of 79 behavioural, environmental and occupational, and metabolic risks or clusters of risks, 1990–2015: a systematic analysis for the Global Burden of Disease Study 2015. *The Lancet* 2016; **388**: 1659–724.

9 Lim SS, Vos T, Flaxman AD, et al. A comparative risk assessment of burden of disease and injury attributable to 67 risk factors and risk factor clusters in 21 regions, 1990-2010: a systematic analysis for the Global Burden of Disease Study 2010. The Lancet 2012; 380: 2224–60.

10 Smith KR, Bruce N, Balakrishnan K, *et al.* Millions Dead: How Do We Know and What Does It Mean? Methods Used in the Comparative Risk Assessment of Household Air Pollution. *Annual Review of Public Health* 2014; **35**: 185–206.

11 WHO. WHO global air quality guidelines: particulate matter (PM2.5 and PM10), ozone, nitrogen dioxide, sulfur dioxide and carbon monoxide. World Health Organization, 2021 https://www.who.int/publications/i/item/9789240034228 (accessed Nov 14, 2024).

12 WHO. Global database of household air pollution measurements. World Health Organization. https://www.who.int/data/gho/data/themes/air-pollution/hap-measurement-db (accessed Nov 12, 2019).

13 Shupler M, Balakrishnan K, Ghosh S, *et al.* Global household air pollution database: Kitchen concentrations and personal exposures of particulate matter and carbon monoxide. *Data in Brief* 2018; **21**: 1292–5.

14 Shupler M, Hystad P, Birch A, *et al.* Household and personal air pollution exposure measurements from 120 communities in eight countries: results from the PURE-AIR study. *The Lancet Planet Health* 2020; **4**: e451–62.

15 Stanaway JD, Afshin A, Gakidou E, *et al.* Global, regional, and national comparative risk assessment of 84 behavioural, environmental and occupational, and metabolic risks or clusters of risks for 195 countries and territories, 1990–2017: a systematic analysis for the Global Burden of Disease Study 2017. *The Lancet* 2018; **392**: 1923–94.

16 Ghosh R, Causey K, Burkart K, Wozniak S, Cohen A, Brauer M. Ambient and household PM2.5 pollution and adverse perinatal outcomes: A meta-regression and analysis of attributable global burden for 204 countries and territories. *PLOS Medicine* 2021; **18**: e1003718.

17 EPA. Integrated Science Assessment (ISA) for Particulate Matter (Final Report, Dec 2009). Washington DC: United States Environmental Protection Agency, 2009 https://cfpub.epa.gov/ncea/risk/recordisplay.cfm?deid=216546 (accessed Oct 27, 2024).

18 Review of evidence on health aspects of air pollution: REVIHAAP project: technical report. Copenhagen Ø, Denmark: WHO Regional Office for Europe https://www.who.int/europe/publications/i/item/WHO-EURO-2013-4101-43860-61757 (accessed Oct 27, 2024).

19 Yin P, Brauer M, Cohen A, *et al.* Long-term Fine Particulate Matter Exposure and Nonaccidental and Cause-specific Mortality in a Large National Cohort of Chinese Men. *Environ Health Perspect* 2017; **125**: 117002.

20 Li T, Zhang Y, Wang J, *et al.* All-cause mortality risk associated with long-term exposure to ambient PM2·5 in China: a cohort study. *The Lancet Public Health* 2018; **3**: e470–7.

21 Yang Y, Tang R, Qiu H, *et al.* Long term exposure to air pollution and mortality in an elderly cohort in Hong Kong. *Environ Int* 2018; **117**: 99–106.

22 Hystad P, Larkin A, Rangarajan S, *et al.* Associations of outdoor fine particulate air pollution and cardiovascular disease in 157 436 individuals from 21 high-income, middle-income, and low-income countries (PURE): a prospective cohort study. *The Lancet Planetary Health* 2020; **4**: e235–45.

23 Burnett RT, Pope CA, Ezzati M, *et al.* An Integrated Risk Function for Estimating the Global Burden of Disease Attributable to Ambient Fine Particulate Matter Exposure. *Environmental Health Perspectives* 2014; **122**: 397–403.

24 Lind L, Sundström J, Ärnlöv J, Lampa E. Impact of Aging on the Strength of Cardiovascular Risk Factors: A Longitudinal Study Over 40 Years. *Journal of the American Heart Association* 2018; **7**: e007061.

25 Tanchangya J, Geater AF. Use of traditional cooking fuels and the risk of young adult cataract in rural Bangladesh: a hospital-based case-control study. BMC Ophthalmology. 2011; 11.

26 Jack DW, Ae-Ngibise KA, Gould CF, *et al.* A cluster randomised trial of cookstove interventions to improve infant health in Ghana. *BMJ Global Health* 2021; **6**: e005599.

27 McCollum ED, McCracken JP, Kirby MA, *et al.* Liquefied Petroleum Gas or Biomass Cooking and Severe Infant Pneumonia. *N Engl J Med* 2024; **390**: 32–43.

28 Clasen TF, Chang HH, Thompson LM, *et al.* Liquefied Petroleum Gas or Biomass for Cooking and Effects on Birth Weight. *N Engl J Med* 2022; **387**: 1735–46.

29 Younger A, Alkon A, Harknett K, Jean Louis R, Thompson LM. Adverse birth outcomes associated with household air pollution from unclean cooking fuels in low- and middle-income countries: A systematic review. *Environmental Research* 2022; **204**: 112274.
