# Supplementary material for: Global, regional, and national burden of household air pollution, 1990–2021: a systematic analysis for the Global Burden of Disease Study 2021
Source: Lancet. 2025 Apr 5;405(10485):1167–81. doi: 10.1016/S0140-6736(24)02840-X (PMC11971481; doi:10.1016/S0140-6736(24)02840-X)
Supplement: Supplementary appendix 3 [file mmc3.pdf]

THE LANCET

Supplementary appendix 3

This appendix formed part of the original submission and has been peer reviewed. We post it as supplied by the authors.

Supplement to: GBD 2021 HAP Collaborators. Global, regional, and national burden of household air pollution, 1990–2021: a systematic analysis for the Global Burden of Disease Study 2021. *Lancet* 2025; published online March 18. https://doi.org/10.1016/S0140-6736(24)02840-X.

Appendix 3: Authorship appendix to "Global, regional, and national burden of household air pollution, 1990–2021: a systematic examination for the Global Burden of Disease Study 2021"

This appendix provides further authorship detail for "Global, regional, and national burden of household air pollution, 1990–2021: a systematic examination for the Global Burden of Disease Study 2021"

Table of Contents

GBD 2021 HAP Collaborators **2**

Affiliations **3**

Authors' Contributions **15**

- Managing the overall research enterprise 15
- Writing the first draft of the manuscript 15
- Primary responsibility for applying analytical methods to produce estimates 15
- Primary responsibility for seeking, cataloguing, extracting, or cleaning data; designing or coding figures and tables 15
- Providing data or critical feedback on data sources 15
- Developing methods or computational machinery 16
- Providing critical feedback on methods or results 16
- Drafting the work or revising it critically for important intellectual content 18
- Managing the estimation or publications process 19

GBD 2021 HAP Collaborators

Fiona B Bennitt, Sarah Wozniak, Kate Causey, Sandra Spearman, Chukwuma Okereke, Vanessa Garcia, Nadim Hashmeh, Charlie Ashbaugh, Atef Abdelkader, Meriem Abdoun, Muhammed Jemal Abdurebi, Armita Abedi, Roberto Ariel Abeldaño Zuñiga, Richard Gyan Aboagye, Bilyaminu Abubakar, Ahmed Abu-Zaid, Mesafint Molla Adane, Oyelola A Adegboye, Victor Adekanmbi, Abiola Victor Adepoju, Temitayo Esther Adeyeoluwa, Olorunsola Israel Adeyomoye, Rishan Adha, Muhammad Sohail Afzal, Saira Afzal, Feleke Doyore Agide, Aqeel Ahmad, Danish Ahmad, Muayyad M Ahmad, Sajjad Ahmad, Ali Ahmadi, Sepideh Ahmadi, Anisuddin Ahmed, Ayman Ahmed, Haroon Ahmed, Marjan Ajami, Rufus Olusola Akinyemi, Salah Al Awaidy, Hanadi Al Hamad, Muaaz M Alajlani, Mulubirhan Assefa Alemayohu, Adel Ali Saeed Al-Gheethi, Abid Ali, Waad Ali, Sheikh Mohammad Alif, Sami Almustanyir, Nelson Alvis-Guzman, Nelson J Alvis-Zakzuk, Hany Aly, Hubert Amu, Ganiyu Adeniyi Amusa, Tadele Fentabel Anagaw, Boluwatife Stephen Anuoluwa, Iyadunni Adesola Anuoluwa, Saeid Anvari, Ekenedilichukwu Emmanuel Anyabolo, Geminn Louis Carace Apostol, Aleksandr Y Aravkin, Demelash Areda, Brhane Berhe Aregawi, Olatunde Aremu, Akeza Awealom Asgedom, Mubarek Yesse Ashemo, Tahira Ashraf, Seyyed Shamsadin Athari, Sina Azadnajafabad, Ahmed Y Azzam, Giridhara Rathnaiah Babu, Saeed Bahramian, Kiran Bam, Maciej Banach, Biswajit Banik, Mehmet Firat Baran, Francesco Barone-Adesi, Sandra Barteit, Hameed Akande Bashiru, Pritish Baskaran, Mohammad-Mahdi Bastan, Sanjay Basu, Saurav Basu, Sefealem Assefa Belay, Melesse Belayneh, Apostolos Beloukas, Derrick A Bennett, Devidas S Bhagat, Dinesh Bhandari, Pankaj Bhardwaj, Sonu Bhaskar, Ajay Nagesh Bhat, Priyadarshini Bhattacharjee, Gurjit Kaur Bhatti, Manpreet S Singh Bhatti, Cem Bilgin, Mary Sefa Boampong, Sri Harsha Boppana, Samuel Adolf Bosoka, Sofiane Boudalia, Fan Cao, Rama Mohan Chandika, Gashaw Sisay Chanie, Vijay Kumar Chattu, Anis Ahmad Chaudhary, Akhilanand Chaurasia, Guangjin Chen, Yifan Chen, Ritesh Chimoriya, Bryan Chong, Devasahayam J Christopher, Isaac Sunday Chukwu, Aaron J Cohen, Natalia Cruz-Martins, Omid Dadras, Xiaochen Dai, Patience Unekwuojo Daikwo, Samuel Demissie Darcho, Saswati Das, Juana Maria Delgado-Saborit, Belay Desye, Sagnik Dey, Meghnath Dhimal, Daniel Diaz, Thanh Chi Do, Ojas Prakashbhai Doshi, Abdel Rahman E'mar, Alireza Ebrahimi, Hisham Atan Edinur, Aziz Eftekharimehrabad, Temitope Cyrus Ekundayo, Ibrahim Farahat El Bayoumy, Syed Emdadul Haque, Theophilus I Emeto, Habtamu Demelash Enyew, Ayesha Fahim, Adekunle Gregory Fakunle, Sasan Faridi, Timur Fazylov, Alireza Feizkhah, Florian Fischer, Morenike Oluwatoyin Folayan, Sridevi G, Muktar A Gadanya, Xiang Gao, Miglas Welay Gebregergis, Mesfin Gebrehiwot, Teferi Gebru Gebremeskel, Afsaneh Ghasemzadeh, Nermin Ghith, Mahaveer Golechha, Davide Golinelli, Shi-Yang Guan, Zhifeng Guo, Bhawna Gupta, Lalit Gupta, Rabih Halwani, Ahmed I Hasaballah, Md Saquib Hasnain, Simon I Hay, Demisu Zenbaba Heyi, Kamal Hezam, Nguyen Quoc Hoan, Ramesh Holla, Hassan Hosseinzadeh, Chengxi Hu, Hong-Han Huynh, Bing-Fang Hwang, Segun Emmanuel Ibitoye, Oluwatope Olaniyi Idowu, Adalia Ikiroma, Mustapha Immurana, Arit Inok, Muhammad Iqhrammullah, Rakibul M Islam, Sheikh Mohammed Shariful Islam, Vinothini J, Ammar Abdulrahman Jairoun, Abhishek Jaiswal, Mihajlo Jakovljevic, Reza Jalilzadeh Yengejeh, Manthan Dilipkumar Janodia, Shubha Jayaram, Alelign Tasew Jema, Ravi Prakash Jha, Jost B Jonas, Nitin Joseph, Vidya Kadashetti, Kehinde Kazeem Kanmodi, Sushil Kumar Kansal, Ibraheem M Karaye, Gbenga A Kayode, Himanshu Khajuria, Amirmohammad Khalaji, Vishnu Khanal, Khaled Khatab, Khalid A Kheirallah, Atulya Aman Khosla, Majid Khosravi, Shivakumar KM, Luke D Knibbs, Gerbrand Koren, Parvaiz A Koul, Kewal Krishan, Barthelemy Kuate Defo, Mohammed Kuddus, Mukhtar Kulimbet, Vishnutheertha Kulkarni, Ashish Kumar, Dewesh Kumar, Nithin Kumar, Om P Kurmi, Chandrakant Lahariya, Hanpeng Lai, Tuo Lan, Paolo Lauriola, Nhi Huu Hanh Le, Munjae Lee, Seung Won Lee, Stephen S Lim, Gang Liu, Shuke Liu, Wei Liu, José Francisco López-Gil, Jay B Lusk, Sandeep B Maharaj, Kashish Malhotra, Ahmad Azam

Malik, Iram Malik, Lesibana Anthony Malinga, Alexander G Mathioudakis, Rita Mattiello, Andrea Maugeri, Tesfahun Mekene Meto, Hadush Negash Meles, Ritesh G Menezes, Sultan Ayoub Meo, Seid Tiku Mereta, Tuomo J Meretoja, Tomislav Mestrovic, Laurette Mhlanga, Ted R Miller, Andreea Mirica, Erkin M Mirrakhimov, Moonis Mirza, Awoke Misganaw, Prasanna Mithra, Jama Mohamed, Nouh Saad Mohamed, Abdollah Mohammadian-Hafshejani, Mustapha Mohammed, Shafiu Mohammed, Ali H Mokdad, Shaher Momani, Himel Mondal, Lidia Morawska, Rohith Motappa, Sumaira Mubarik, Kavita Munjal, Yanjinlkham Munkhsaikhan, Christopher J L Murray, Woojae Myung, Sanjeev Nair, Vinay Nangia, Muhammad Naveed, Nawsherwan, Rawlance Ndejjo, Dang Nguyen, Hien Quang Nguyen, Van Thanh Nguyen, Taxiarchis Konstantinos Nikolouzakis, Vikram Niranjan, Efaq Ali Noman, Syed Toukir Ahmed Noor, Abbas Norouzian Baghani, Jean Jacques Noubiap, Ogochukwu Janet Nzoputam, Bogdan Oancea, Ismail A Odetokun, Daniel Bogale Odo, Akinyemi O D Ofakunrin, Onome Bright Oghenetega, Osaretin Christabel Okonji, Andrew T Olagunju, Tosin Abiola Olasehinde, Isaac Iyinoluwa Olufadewa, Gideon Olamilekan Oluwatunase, Ahmed Omar Bali, Mohammad Mehdi Ommati, Abiodun Olusola Omotayo, Maureene Auma Ondayo, Adrian Otoiu, Mayowa O Owolabi, Mahesh Padukudru P A, Jagadish Rao Padubidri, Ioannis Pantazopoulos, Shahina Pardhan, Pragyan Paramita Parija, Romil R Parikh, Eun-Kee Park, Ashwaghosha Parthasarathi, Jay Patel, Siddhartha Pati, Shrikant Pawar, Prince Peprah, Gavin Pereira, Arokiasamy Perianayagam, Hoang Tran Pham, Ramesh Poluru, Akram Pourshams, Jalandhar Pradhan, Elton Junio Sady Prates, Dimas Ria Angga Pribadi, Jagadeesh Puvvula, Ata Rafiee, Pankaja Raghav, Fakher Rahim, Mohammad Hifz Ur Rahman, Mosiur Rahman, Muhammad Aziz Rahman, Amir Masoud Rahmani, Mohammad Rahmanian, Sathish Rajaa, Rayan Rajabi, Prashant Rajput, Mahmoud Mohammed Ramadan, Juwel Rana, Kritika Rana, Chhabi Lal Ranabhat, Drona Prakash Rasali, Santosh Kumar Rauniyar, Salman Rawaf, Elrashdy M Moustafa Mohamed Redwan, Nazila Rezaei, Jefferson Antonio Buendia Rodriguez, Susanne Röhr, Gholamreza Roshandel, Himanshu Sekhar Rout, Priyanka Roy, Michele Russo, Cameron John Sabet, Basema Ahmad Saddik, Umar Saeed, Narjes Saheb Sharif-Askari, Amirhossein Sahebkar, Pragyan Monalisa Sahoo, Afeez Abolarinwa Salami, Dauda Salihu, Abdallah M Samy, Milena M Santric-Milicevic, Tanmay Sarkar, Maheswar Satpathy, Ganesh Kumar Saya, Md Abu Sayeed, Austin E Schumacher, Mihretu Tagesse Sergindo, Yashendra Sethi, Allen Seylani, Samiah Shahid, Sunder Sham, Muhammad Aaqib Shamim, Anas Shamsi, Aziz Sheikh, Pavanchand H Shetty, Aminu Shittu, Ivy Shiue, Emmanuel Edwar Siddig, Paramdeep Singh, Surjit Singh, Md Shahjahan Siraj, Jeffrey D Stanaway, Leo Stockfelt, Kurt Straif, Chandan Kumar Swain, Berwin Singh Swami Vetha, Seyyed Mohammad Tabatabaei, Mircea Tampa, Haosu Tang, Manoj Tanwar, Elvis Enowbeyang Tarkang, Yibekal Manaye Tefera, Mohamad-Hani Temsah, Reem Mohamad Hani Temsah, Ramna Thakur, Friedrich Thienemann, Nigusie Selomon Tibebu, Krishna Tiwari, Marcos Roberto Tovani-Palone, Jaya Prasad Tripathy, Aristidis Tsatsakis, Munkhtuya Tumurkhuu, Aniefiok John Udoakang, Sana Ullah, Sanaz Vahdati, Siavash Vaziri, Madhur Verma, Simone Vidale, Simona Villani, Karn Vohra, Theo Vos, Gizachew Tadesse Wassie, Haftom Legese Weldetinsaa, Adhena Ayaliew Werkneh, Nuwan Darshana Wickramasinghe, Marcin W Wojewodzic, Tewodros Eshete Wonde, Felicia Wu, Zenghong Wu, Hong Xiao, Suowen Xu, Mukesh Kumar Yadav, Saba Yahoo Syed, Sanni Yaya, Arzu Yiğit, Vahit Yiğit, Dehui Yin, Dong Keon Yon, Naohiro Yonemoto, Chuanhua Yu, Leila Zaki, Mohammed G M Zeariya, Youjie Zeng, Chunxia Zhai, Haijun Zhang, Zhiqiang Zhang, Bin Zhu, Sa'ed H Zyoud, Samer H Zyoud, Michael Brauer, and Katrin Burkart.

Affiliations

Department of Civil and Environmental Engineering (F B Bennitt BA), University of Massachusetts Amherst, Amherst, MA, USA; Weill Cornell Medical College (S Wozniak MPH), Weill Cornell Medicine, New York, NY, USA; Research, Insights, and Innovation (K Causey MPH), Independent Consultant,

Seattle, WA, USA; Institute for Health Metrics and Evaluation (S Spearman MS, C Okereke MPH, V Garcia BS, N Hashmeh MSc, C Ashbaugh MA, A Y Aravkin PhD, A J Cohen DSc, X Dai PhD, Prof S I Hay FMedSci, Prof S S Lim PhD, T Mestrovic PhD, Prof A H Mokdad PhD, Prof C J L Murray DPhil, A E Schumacher PhD, J D Stanaway PhD, Prof T Vos PhD, Prof M Brauer DSc, K Burkart PhD), Department of Applied Mathematics (A Y Aravkin PhD), Department of Health Metrics Sciences, School of Medicine (A Y Aravkin PhD, X Dai PhD, Prof S I Hay FMedSci, Prof S S Lim PhD, A Misganaw PhD, Prof A H Mokdad PhD, Prof C J L Murray DPhil, J D Stanaway PhD, Prof T Vos PhD, K Burkart PhD), University of Washington, Seattle, WA, USA; Department of Mathematics and Sciences (A Abdelkader PhD), Nonlinear Dynamics Research Center (NDRC) (Prof S Momani PhD), Center for Medical and Bio-Allied Health Sciences Research (A Shamsi PhD, S H Zyoud PhD), Ajman University, Ajman, United Arab Emirates; Department of Medicine (Prof M Abdoun PhD), University of Setif Algeria, Sétif, Algeria; Department of Health, Sétif, Algeria (Prof M Abdoun PhD); Department of Public Health (M J Abdurebi MPH), Bule Hora University, Bule Hora, Ethiopia; Department of Nursing (M J Abdurebi MPH), Rift Valley University, Robe, Ethiopia; Department of Emergency Medicine (A Abedi MD), Department of Immunology (S Athari PhD), Zanjan University of Medical Sciences, Zanjan, Iran; Postgraduate Department (Prof R Abeldaño Zuñiga PhD), University of Sierra Sur, Miahuatlan de Porfirio Diaz, Mexico; Yhteiskuntadatatieteen keskus (Centre for Social Data Science) (Prof R Abeldaño Zuñiga PhD), University of Helsinki, Helsinki, Finland (T J Meretoja MD); Department of Family and Community Health (R G Aboagye MPH), Department of Population and Behavioural Sciences (H Amu PhD, Prof E E Tarkang PhD), Department of Epidemiology and Biostatistics (S A Bosoka MPhil), Institute of Health Research (M Immurana PhD), University of Health and Allied Sciences, Ho, Ghana; Department of Pharmacology and Toxicology (B Abubakar PhD), Department of Veterinary Public Health and Preventive Medicine (A Shittu MSc), Usmanu Danfodiyo University, Sokoto, Sokoto, Nigeria; Clinical Science Department (Prof M O Folayan PhD), Nigerian Institute of Medical Research, Lagos, Nigeria (B Abubakar PhD); Department of Biochemistry and Molecular Medicine (A Abu-Zaid PhD), College of Medicine (S Almustanyir MD), College of Pharmacy (R M H Temsah PharmD), Alfaisal University, Riyadh, Saudi Arabia; College of Graduate Health Sciences (A Abu-Zaid PhD), University of Tennessee, Memphis, TN, USA; College of Medicine and Health Sciences (M M Adane PhD), Department of Health Promotion (T F Anagaw MPH), Department of Biomedical Sciences (S A Belay MSc), Department of Public Health (M Belayneh PhD), Department of Epidemiology and Biostatistics (G T Wassie MPH), Bahir Dar University, Bahir Dar, Ethiopia; Menzies School of Health Research (Prof O A Adegboye PhD), Charles Darwin University, Darwin, NT, Australia; Department of Obstetrics and Gynecology (V Adekanmbi PhD), University of Texas Medical Branch, Galveston, TX, USA; Department of HIV and Infectious Diseases (A V Adepoju MD), Jhpiego, Abuja, Nigeria; Department of Adolescent Research and Care (A V Adepoju MD), Adolescent Friendly Research Initiative and Care, Ado Ekiti, Nigeria; Department of Pharmacology and Therapeutics (T E Adeyeoluwa PhD), Department of Physiology (O I Adeyomoye PhD), Department of Environmental and Occupational Health (B S Anuoluwa MPH, P U Daikwo MSc), Department of Microbiology (I A Anuoluwa PhD, T C Ekundayo PhD), Department of Chemistry (O O Idowu MSc), Department of Anatomy (G O Oluwatunase MSc), Department of Biosciences and Biotechnology (A J Udoakang PhD), University of Medical Sciences, Ondo, Ondo, Nigeria; Department of Veterinary Medicine (T E Adeyeoluwa PhD), Institute for Advanced Medical Research and Training (Prof R O Akinyemi PhD), Department of Health Promotion and Education (S Ibitoye PhD), Faculty of Public Health (I I Olufadewa MHS), Department of Medicine (Prof M O Owolabi DrM), University of Ibadan, Ibadan, Nigeria; Department of Statistics (R Adha PhD), National Chengchi University, Taipei, Taiwan; Department of Life Sciences (M S Afzal PhD), University of

Management and Technology, Lahore, Pakistan; Department of Community Medicine (Prof S Afzal PhD), King Edward Memorial Hospital, Lahore, Pakistan; Department of Public Health (Prof S Afzal PhD), Public Health Institute, Lahore, Pakistan; Department of Health Education and Health Promotion (F D Agide PhD), Department of Public Health (M Y Ashemo MPH, M T Sergindo MSc), Wachemo University, Hossana, Ethiopia; College of Medicine (A Ahmad PhD), Shaqra University, Shaqra, Saudi Arabia; School of Medicine and Psychology (D Ahmad PhD), Australian National University, Canberra, ACT, Australia; Public Health Foundation of India, Gandhinagar, India (D Ahmad PhD); School of Nursing (Prof M M Ahmad PhD), University of Jordan, Amman, Jordan; Department of Health and Biological Sciences (S Ahmad PhD), Abasyn University, Peshawar, Pakistan; Department of Natural Sciences (S Ahmad PhD), Lebanese American University, Beirut, Lebanon; Department of Epidemiology and Biostatistics (A Ahmadi PhD), Modeling in Health Research Center (A Mohammadian-Hafshejani PhD), Shahrekord University of Medical Sciences, Shahrekord, Iran; Department of Epidemiology (A Ahmadi PhD), School of Advanced Technologies in Medicine (S Ahmadi PhD), National Nutrition and Food Technology Research Institute (M Ajami PhD), Student Research Committee (M Rahmanian MD), Shahid Beheshti University of Medical Sciences, Tehran, Iran; Maternal and Child Health Division (A Ahmed MS, S Noor MS, M Siraj MMedSc), International Centre for Diarrhoeal Disease Research, Bangladesh, Dhaka, Bangladesh; Department of Women's and Children's Health (A Ahmed MS), Uppsala University, Uppsala, Sweden; Institute of Endemic Diseases (A Ahmed MSc), Unit of Basic Medical Sciences (E E Siddig MD), University of Khartoum, Khartoum, Sudan; Swiss Tropical and Public Health Institute (A Ahmed MSc), University of Basel, Basel, Switzerland; Department of Biosciences (H Ahmed PhD), COMSATS Institute of Information Technology, Islamabad, Pakistan; Institute of Neuroscience (Prof R O Akinyemi PhD), The Translational and Clinical Institute (P Bhattacharjee MD), Newcastle University, Newcastle upon Tyne, UK; Department of Communicable Diseases (S Al Awaidy MSc), Ministry of Health, Muscat, Oman; Middle East, Eurasia, and Africa Influenza Stakeholders Network, Muscat, Oman (S Al Awaidy MSc); Department of Geriatric and Long Term Care (H Al Hamad MD), Rumailah Hospital (H Al Hamad MD), Hamad Medical Corporation, Doha, Qatar; Faculty of Pharmacy (Prof M M Alajlani PhD), Al-Sham Private University, Damascus, Syria; Department of Biostatistics and Clinical Epidemiology (M Alemayohu PhD), Department of Public Health, Experimental and Forensic Medicine (Prof S Villani PhD), University of Pavia, Pavia, Italy; Department of Epidemiology (M Alemayohu PhD), Department of Environmental Health (A A Asgedom PhD), Department of Environmental Health and Behavioral Sciences (A A Werkneh MSc), Mekelle University, Mekelle, Ethiopia; Global Centre for Environmental Remediation (A A S Al-Gheethi PhD), University of Newcastle, Newcastle, NSW, Australia; Cooperative Research Centre for Contamination Assessment and Remediation of the Environment, Newcastle, NSW, Australia (A A S Al-Gheethi PhD); Department of Zoology (A Ali PhD), Abdul Wali Khan University Mardan, Mardan, Pakistan; Department of Geography (W Ali PhD), Sultan Qaboos University, Muscat, Oman; Institute of Health and Wellbeing (S M Alif PhD, B Banik PhD), Federation University Australia, Melbourne, VIC, Australia; School of Public Health and Preventive Medicine (S M Alif PhD), Department of Epidemiology and Preventive Medicine (R M Islam PhD), Monash University, Melbourne, VIC, Australia; Ministry of Health, Riyadh, Saudi Arabia (S Almustanyir MD); Research Group in Health Economics (Prof N Alvis-Guzman PhD), Universidad de Cartagena (University of Cartagena), Cartagena, Colombia; Research Group in Hospital Management and Health Policies (Prof N Alvis-Guzman PhD), Department of Economic Sciences (N J Alvis-Zakzuk MSc), Universidad de la Costa (University of the Coast), Barranquilla, Colombia; National Health Observatory (N J Alvis-Zakzuk MSc), National Institute of Health, Bogota, Colombia; Department of Pediatrics (Prof H Aly MD, A E’mar MD), Cleveland Clinic, Cleveland, OH, USA;

Department of Medicine (G A Amusa MD), Department of Pediatrics (A O D Ofakunrin MD), University of Jos, Jos, Nigeria; Department of Internal Medicine (G A Amusa MD), Department of Pediatrics (A O D Ofakunrin MD), Jos University Teaching Hospital, Jos, Nigeria; Regenerative Medicine, Organ Procurement and Transplantation Multi-disciplinary Center (S Anvari MD), Department of Social Medicine and Epidemiology (A Feizkhah MD), Guilan University of Medical Sciences, Rasht, Iran; Department of Medical Laboratory Sciences (E E Anyabolo BMLS), University of Nigeria Nsukka, Enugu, Nigeria; Operations Department (E E Anyabolo BMLS), Breast Without Spot, Enugu, Nigeria; School of Medicine and Public Health (G C Apostol MD), Ateneo De Manila University, Pasig City, Philippines; Inter-Agency Committee on Environmental Health (G C Apostol MD), Department of Health Philippines, Manila, Philippines; College of Art and Science (D Areda PhD), Ottawa University, Surprise, AZ, USA; School of Life Sciences (D Areda PhD), Arizona State University, Tempe, AZ, USA; College of Medicine and Health Sciences (B B Aregawi PhD), Department of Midwifery (M W Gebregergis MSc), Department of Medical Laboratory Sciences (H N Meles MSc, H L Weldetinsaa MSc), Adigrat University, Adigrat, Ethiopia; Department of Public Health (O Aremu PhD), Birmingham City University, Birmingham, UK; Department of Public Health (M Y Ashemo MPH), Department of Environmental Health Sciences and Technology (S Mereta PhD), Jimma University, Jimma, Ethiopia; Pioneer Journal of Biostatistics and Medical Research (PJBMR), Pakistan, Pakistan (T Ashraf PhD); Department of Surgery (S Azadnajafabad MD), Washington University in St. Louis, St. Louis, MO, USA; Leeds Institute of Rheumatic and Musculoskeletal Medicine (S Azadnajafabad MD), School of Dentistry (J Patel BSc), University of Leeds, Leeds, UK; ASIDE Healthcare, Lewes, DE, USA (A Azzam MD); Faculty of Medicine (A Azzam MD), October 6 University, 6th of October City, Egypt; Department of Population Medicine (Prof G Babu PhD), QU Health (M Mohammed PhD), Social and Economic Survey Research Institute (Prof A Perianayagam PhD), Qatar University, Doha, Qatar; School of Medicine (S Bahramian MD), Isfahan University of Medical Sciences, Isfahan, Iran; Department of Medicine (K Bam MPH), School of Nursing and Midwifery (D Bhandari PhD), Monash University, Clayton, VIC, Australia; Department of Hypertension (Prof M Banach PhD), Medical University of Lodz, Lodz, Poland; Polish Mothers' Memorial Hospital Research Institute, Lodz, Poland (Prof M Banach PhD); Manna Institute (B Banik PhD), University of New England, Armidale, NSW, Australia; Vocational School of Technical Sciences (M Baran PhD), Batman University, Batman, Turkiye; Department of Translational Medicine (F Barone-Adesi PhD), University of Eastern Piedmont, Novara, Italy; Heidelberg Institute of Global Health (HIGH) (S Barteit PhD), Heidelberg University Hospital, Heidelberg, Germany; Department of Animal Sciences (H A Bashiru PhD), Department of Child Dental Health (Prof M O Folayan PhD), Obafemi Awolowo University, Ile-Ife, Nigeria; Department of Community Medicine (P Baskaran MD), Sri Manakula Vinayagar Medical College and Hospital, Puducherry, Puducherry, India; Non-communicable Diseases Research Center (M Bastan MD, N Rezaei MD), Iranian Research Center for HIV/AIDS (IRCHA) (O Dadras PhD), Institute for Environmental Research (S Faridi PhD), School of Medicine (A Khalaji MD), Digestive Diseases Research Institute (Prof A Pourshams MD), Tehran University of Medical Sciences, Tehran, Iran; School of Medicine (M Bastan MD), Department of Health Economics (M Khosravi PhD), Department of Medicine (R Rajabi MD), Iran University of Medical Sciences, Tehran, Iran; Center for Primary Care (S Basu PhD), Division of General Internal Medicine (Prof A Sheikh MD), Harvard University, Boston, MA, USA; School of Public Health (S Basu PhD), Department of Primary Care and Public Health (Prof S Rawaf MD), The George Institute for Global Health (Prof S Yaya PhD), Imperial College London, London, UK; Department of Academics (S Basu MD), Indian Institute of Public Health, Gurgaon, India; Department of Public Health (M Belayneh PhD), University of South Africa, Pretoria, South Africa; Department of Biomedical Sciences (Prof A Beloukas

PhD), National AIDS Reference Center of Southern Greece (Prof A Beloukas PhD), University of West Attica, Athens, Greece; Nuffield Department of Population Health (D A Bennett PhD), University of Oxford, Oxford, UK; Department of Forensic Chemistry (D S Bhagat PhD), Government Institute of Forensic Science, Aurangabad, Aurangabad, India; School of Public Health (D Bhandari PhD), University of Adelaide, Adelaide, SA, Australia; Department of Community Medicine and Family Medicine (Prof P Bhardwaj MD, Prof P Raghav MD), School of Public Health (Prof P Bhardwaj MD), Department of Pharmacology (M Shamim MBBS, S Singh MD, K Tiwari MBBS), All India Institute of Medical Sciences, Jodhpur, India; Global Health Neurology Lab (S Bhaskar MD), NSW Brain Clot Bank, Sydney, NSW, Australia; Division of Cerebrovascular Medicine and Neurology (S Bhaskar MD), National Cerebral and Cardiovascular Center, Suita, Japan; Department of General Medicine (A N Bhat MD), Department of Community Medicine (N Joseph MD, N Kumar MD, P Mithra MD, R Motappa MD), Department of Forensic Medicine and Toxicology (Prof J Padubidri MD, P H Shetty MD), Manipal Academy of Higher Education, Mangalore, India; Department of Clinical Medicine (P Bhattacharjee MD), Cambridge University Hospitals NHS Foundation Trust, Cambridge, UK; Department of Medical Lab Technology (Prof G K Bhatti PhD), Chandigarh University, Mohali, India; Department of Botanical and Environmental Sciences (Prof M S S Bhatti PhD), Guru Nanak Dev University, Amritsar, India; Department of Radiology (C Bilgin MD), Neurovascular Research Laboratory (C Bilgin MD), Mayo Clinic College of Medicine, Rochester, MN, USA; Department of Sociology and Social Work (M S Boampong PhD), Kwame Nkrumah University of Science and Technology, Kumasi, Ghana; Department of Anesthesia and Critical Care Medicine (S Boppana MD), Department of International Health (H Zhang MS), Johns Hopkins University, Baltimore, MD, USA; Disease Surveillance Department (S A Bosoka MPhil), Ghana Health Service, Ho, Ghana; Faculty of Natural Sciences and Life Sciences (Prof S Boudalia PhD), Guelma University, Guelma, Algeria; Department of Ophthalmology (F Cao MD), Beijing Institute of Ophthalmology, Beijing, China; Clinical Nutrition Department (R M Chandika PhD), Jazan University, Jazan, Saudi Arabia; Department of Clinical Pharmacy (G S Chanie MSc), University of Gondar, Gondar, Ethiopia; Temerty Faculty of Medicine (V Chattu MD), University of Toronto, Toronto, ON, Canada; Department of Community Medicine (V Chattu MD), Datta Meghe Institute of Medical Sciences, Sawangi, India; Department of Biology (A A Chaudhary PhD), Al-Imam Mohammad Ibn Saud Islamic University, Riyadh, Saudi Arabia; Department of Oral Medicine and Radiology (Prof A Chaurasia MD), King George's Medical University, Lucknow, India; Department of Stomatology (G Chen DMD), Department of Occupational and Environmental Health (H Lai PhD), Division of Gastroenterology (Prof Z Wu PhD), Huazhong University of Science and Technology, Wuhan, China; Hubei Province Key Laboratory of Oral and Maxillofacial Development and Regeneration, Wuhan, China (G Chen DMD); Centre for Health Management and Policy Research (Y Chen MPH), National Health Commission of China (NHC) Key Laboratory of Health Economics and Policy Research (Y Chen MPH), Shandong University, Jinan, China; Concord Institute of Academic Surgery (R Chimoriya PhD), Sydney Local Health District, Sydney, NSW, Australia; Concord Clinical School (R Chimoriya PhD), School of Public Health (L D Knibbs PhD), School of Chemical & Biomolecular Engineering (E A Noman PhD), University of Sydney, Sydney, NSW, Australia; Department of Medicine (B Chong MBBS), National University of Singapore, Singapore, Singapore; Department of Pulmonary Medicine (Prof D J Christopher MD), Christian Medical College and Hospital (CMC), Vellore, India; Department of Paediatric Surgery (I S Chukwu BMedSc), Federal Medical Centre, Umuahia, Nigeria; Health Effects Institute, Boston, MA, USA (A J Cohen DSc); Department of Diagnostic and Therapeutic Technologies (Prof N Cruz-Martins PhD), Cooperativa de Ensino Superior Politécnico e Universitário (Polytechnic and University Higher Education Cooperative), Vila Nova de Famalicão,

Portugal; Institute for Research and Innovation in Health (i3S) (Prof N Cruz-Martins PhD), University of Porto, Porto, Portugal; Research Center for Child Psychiatry (O Dadras PhD), University of Turku, Turku, Finland; Department of Public Health (S D Darcho MPH), Haramaya University, Harar, Ethiopia; Department of Biochemistry (S Das MD), Ministry of Health and Welfare, New Delhi, India; Department of Medicine (J Delgado-Saborit PhD), Universitat Jaume I, Castellon, Spain; Department of Environmental Health (B Desye MSc, M Gebrehiwot DSc), Wollo University, Dessie, Ethiopia; Centre for Atmospheric Sciences (Prof S Dey PhD), Indian Institute of Technology Delhi, New Delhi, India; Research Department (M Dhimal PhD), Nepal Health Research Council, Kathmandu, Nepal; Institute of Occupational, Social and Environmental Medicine (M Dhimal PhD), Goethe University, Frankfurt am Main, Germany; Faculty of Science (Prof D Diaz PhD), National Autonomous University of Mexico, Mexico City, Mexico; Department of Medicine (T C Do MD), Pham Ngoc Thach University of Medicine, Ho Chi Minh City, Vietnam; Independent Consultant, South Plainfield, NJ, USA (O P Doshi MS); Department of Orthopaedic Surgery (A Ebrahimi MD), Massachusetts General Hospital, Boston, MA, USA; School of Health Sciences (H A Edinur PhD), Universiti Sains Malaysia (University of Science Malaysia), Kubang Kerian, Malaysia; Department of Biochemistry (A Eftekharimehrabad PhD), Ege University, Izmir, Turkiye; Azerbaijan State University of Economics (UNEC), Baku, Azerbaijan (A Eftekharimehrabad PhD); Department of Public Health and Community Medicine (Prof I F El Bayoumy DrPH), Tanta University, Tanta city, Egypt; School of Public Health (Prof I F El Bayoumy DrPH), Texila American University, Guyana, Guyana; Department of Research (S E Emdadul Haque PhD), UChicago Research Bangladesh, Dhaka, Bangladesh; Department of Public Health and Tropical Medicine (T I Emeto PhD), James Cook University, Townsville, QLD, Australia; Department of Public Health (H D Enyew PhD), Pediatrics and Child Health Nursing (N Tibebu MSc), Debre Tabor University, Debre Tabor, Ethiopia; Department of Oral Biology (A Fahim PhD), Riphah International University, Islamabad, Pakistan; Department of Public Health (A G Fakunle PhD), Osun State University, Osogbo, Osogbo, Nigeria; Laboratory of Experimental Medicine (T Fazylov MD), Research and Publication Activity Division (M Kulimbet MSc), Kazakh National Medical University, Almaty, Kazakhstan; Institute of Public Health (F Fischer PhD), Charité Universitätsmedizin Berlin (Charité Medical University Berlin), Berlin, Germany; Department of Community Medicine and Family Medicine (S G MD, V J MD), All India Institute of Medical Sciences, Gorakhpur, India; Department of Community Medicine (Prof M A Gadanya MD), Bayero University Kano, Kano, Nigeria; Department of Community Medicine (Prof M A Gadanya MD), Aminu Kano Teaching Hospital, Kano, Nigeria; Department of Biostatistics (Prof X Gao PhD), Key Lab of Environment and Health (Prof X Gao PhD), Department of Epidemiology (D Yin DrPH), Xuzhou Medical University, Xuzhou, China; College of Medicine and Public Health (T G Gebremeskel PhD), Flinders University, Adelaide, SA, Australia; College of Medicine and Public Health (T G Gebremeskel PhD), Aksum University, Aksum, Ethiopia; Immunology Research Center (A Ghasemzadeh MD), Tabriz University of Medical Sciences, Tabriz, Iran; Research Group for Childhood Cancer (N Ghith PhD), Cancer Research Institute, Danish Cancer Society, Copenhagen, Denmark; Department of Health Systems and Policy Research (Prof M Golechha PhD), Indian Institute of Public Health, Gandhinagar, India; Department of Life Sciences, Health and Healthcare Professions (Prof D Golinelli MD), Link Campus University, Rome, Italy; Health Services Research, Evaluation and Policy Unit (Prof D Golinelli MD), AUSL della Romagna, Ravenna, Italy; Department of Epidemiology and Biostatistics (S Guan MD), Anhui Medical University, Hefei, China; Group Health Department (Z Guo MPH), Nanyang Central Hospital, Nanyang, China; Department of Public Health (B Gupta PhD), Torrens University Australia, Melbourne, VIC, Australia; Department of Anaesthesia (Prof L Gupta MD), Maulana Azad Medical College, New Delhi, India; Clinical Sciences Department (Prof R

Halwani PhD, N Saheb Sharif-Askari PhD), College of Medicine (Prof R Halwani PhD, Prof B A Saddik PhD), Department of Clinical Sciences (Prof M M Ramadan PhD), University of Sharjah, Sharjah, United Arab Emirates; Department of Zoology and Entomology (A I Hasaballah PhD, M G M Zeariya PhD), Al-Azhar University, Cairo, Egypt; Department of Pharmacy (Prof M S Hasnain PhD), Marwadi University, Rajkot, India; Department of Public Health (D Z Heyi MPH), Madda Walabu University, Robe, Ethiopia; Department of Microbiology (K Hezam PhD), Faculty of Applied Sciences (E A Noman PhD), Taiz University, Taiz, Yemen; School of Medicine (K Hezam PhD), Nankai University, Tianjin, China; School of Dentistry (N Hoan DDS), Hanoi Medical University, Hanoi, Vietnam; Kasturba Medical College, Mangalore (R Holla MD), Manipal Academy of Higher Education, Manipal, India; School of Health and Society (H Hosseinzadeh PhD), University of Wollongong, Wollongong, NSW, Australia; Department of Psychology (C Hu PhD), Tsinghua University, Beijing, China; International Master Program for Translational Science (H Huynh BS), Taipei Medical University, Taipei, Taiwan; Department of Occupational Safety and Health (Prof B Hwang PhD), China Medical University, Taiwan, Taichung, Taiwan; Department of Occupational Therapy (Prof B Hwang PhD), Asia University, Taiwan, Taichung, Taiwan; Collaborative Alliance Research and Education (CARE) Programme (A Ikiroma PhD), Episcope Research Service, Aberdeen, Scotland; Faculty of Health and Life Sciences (A Inok PhD), University of Exeter, Exeter, UK; Faculty of Public Health (M Iqhrammullah PhD), Universitas Muhammadiyah Aceh, Banda Aceh, Indonesia; Institute for Physical Activity and Nutrition (Prof S Islam PhD), Deakin University, Burwood, VIC, Australia; Department of Health and Safety (A A Jairoun PhD), Dubai Municipality, Dubai, United Arab Emirates; Centre for Community Medicine (A Jaiswal MD), All India Institute of Medical Sciences, New Delhi, India; The World Academy of Sciences UNESCO, Trieste, Italy (Prof M Jakovljevic PhD); Shaanxi University of Technology, Hanzhong, China (Prof M Jakovljevic PhD); Department of Environmental Engineering (Prof R Jalilzadeh Yengejeh PhD), Islamic Azad University, Ahvaz, Iran; Malla Reddy Vishwa Vidyapeeth, Hyderabad, India (Prof M D Janodia PhD); Department of Biochemistry (Prof S Jayaram MD), Government Medical College, Mysuru, India; Department of Public Health (A Jema MPH), Madda Walabu University, Goba, Ethiopia; Department of Community Medicine (R P Jha MSc), Dr. Baba Saheb Ambedkar Medical College & Hospital, Delhi, India; Department of Community Medicine (R P Jha MSc), Banaras Hindu University, Varanasi, India; Rothschild Foundation Hospital (Prof J B Jonas MD), Institut Français de Myopie, Paris, France; Singapore Eye Research Institute (Prof J B Jonas MD), Singapore Eye Research Institute, Singapore, Singapore; Department of Oral and Maxillofacial Pathology (V Kadashetti MDS), Department of Public Health Dentistry (Prof S KM MD), Krishna Vishwa Vidyapeeth (Deemed to be University), Karad, India; Faculty of Dentistry (K K Kanmodi MPH, A A Salami BDS), University of Puthisastra, Phnom Penh, Cambodia; Office of the Executive Director (K K Kanmodi MPH), Cephas Health Research Initiative Inc, Ibadan, Nigeria; Dr. S S Bhatnagar University Institute of Chemical Engineering & Technology (Prof S K Kansal PhD), Department of Anthropology (Prof K Krishan PhD), Panjab University, Chandigarh, India; School of Health Professions and Human Services (I M Karaye MD), Hofstra University, Hempstead, NY, USA; Department of Anesthesiology (I M Karaye MD), Montefiore Medical Center, Bronx, NY, USA; International Research Center of Excellence (G A Kayode PhD), Institute of Human Virology Nigeria, Abuja, Nigeria; Julius Centre for Health Sciences and Primary Care (G A Kayode PhD), Copernicus Institute of Sustainable Development (G Koren PhD), Utrecht University, Utrecht, Netherlands; Amity Institute of Forensic Sciences (H Khajuria PhD), Amity Institute of Pharmacy (K Munjal PhD), Amity University, Noida, India; Endocrinology and Metabolism Research Institute (A Khalaji MD), Non-Communicable Diseases Research Center (NCDRC), Tehran, Iran; Department of Health (V Khanal PhD), Nepal Development Society, Chitwan, Nepal; Department of Preventable Non

Communicable Disease (V Khanal PhD), Menzies School of Health Research, Alice Springs, NT, Australia; College of Health, Wellbeing and Life Sciences (Prof K Khatab PhD), Sheffield Hallam University, Sheffield, UK; College of Arts and Sciences (Prof K Khatab PhD), Ohio University, Zanesville, OH, USA; Department of Public Health (Prof K A Kheirallah PhD), Jordan University of Science and Technology, Irbid, Jordan; Department of Internal Medicine (A A Khosla MD), Corewell Health East William Beaumont University Hospital, Royal Oak, MI, USA; Department of Medical Oncology (A A Khosla MD), Miami Cancer Institute, Miami, FL, USA; Department of Health Management and Economics (M Khosravi PhD), Qom University of Medical Sciences, Qom, Iran; Department of Internal and Pulmonary Medicine (Prof P A Koul MD), Sheri Kashmir Institute of Medical Sciences, Srinagar, India; Department of Demography (Prof B Kuate Defo PhD), Department of Social and Preventive Medicine (Prof B Kuate Defo PhD), University of Montreal, Montreal, QC, Canada; Department of Biochemistry (Prof M Kuddus PhD), Department of Public Health (M G M Zeariya PhD), University of Hail, Hail, Saudi Arabia; Center of Medicine and Public Health (M Kulimbet MSc), Director of Central Asia Research Collaboration Group (Prof F Rahim PhD), Asfendiyarov Kazakh National Medical University, Almaty, Kazakhstan; Department of Medicine (V Kulkarni MS), Queensland Health, Brisbane, QLD, Australia; Department of Cardiovascular Medicine (A Kumar MD), Department of Informatics and Radiology (S Vahdati MD), Mayo Clinic, Rochester, MN, USA; Department of Community Medicine (D Kumar MD), Rajendra Institute of Medical Sciences, Ranchi, India; Faculty of Health and Life Sciences (O P Kurmi PhD), Coventry University, Coventry, UK; Department of Medicine (O P Kurmi PhD), Department of Psychiatry and Behavioural Neurosciences (Prof A T Olagunju PhD), McMaster University, Hamilton, ON, Canada; Integrated Department of Epidemiology, Health Policy, Preventive Medicine and Pediatrics (Prof C Lahariya MD), Foundation for People-centric Health Systems, New Delhi, India; Centre for Health: The Specialty Practice, New Delhi, India (Prof C Lahariya MD); Department of Respiratory and Critical Care Medicine (H Lai PhD), Northern Jiangsu People's Hospital, Yangzhou, China; Department of Surgery (T Lan PhD), Washington University in St. Louis, St Louis, MO, USA; International Society of Doctors for the Environment, Arezzo, Italy (P Lauriola MD); Faculty of Medicine (N Le MD), Department of General Medicine (V T Nguyen MD), University of Medicine and Pharmacy at Ho Chi Minh City, Ho Chi Minh City, Vietnam; Department of Cardiovascular Research (N Le MD), Methodist Hospital, Merrillville, IN, USA; Department of Medical Science (M Lee PhD), Ajou University School of Medicine, Suwon, South Korea; Department of Precision Medicine (Prof S Lee MD), Sungkyunkwan University, Suwon-si, South Korea; School of Life Sciences (G Liu PhD), University of Technology Sydney, Sydney, NSW, Australia; Department of Cardiology (S Liu MSc), Guiqian International General Hospital, Guiyang, China; Institute for Health and Environment (W Liu PhD), Chongqing University of Science and Technology, Chongqing, China; One Health Research Group (J López-Gil PhD), Universidad de Las Américas (University of the Americas), Quito, Ecuador; Department of Population Health Sciences (J B Lusk MD), Duke University, Durham, NC, USA; School of Pharmacy (S B Maharaj DBA), University of the West Indies, St. Augustine, Trinidad and Tobago; Planetary Health Alliance, Boston, MA, USA (S B Maharaj DBA); Rama Medical College Hospital and Research Centre, Uttar Pradesh, India (K Malhotra MBBS); Institute of Applied Health Research (K Malhotra MBBS), Geography, Earth and Environmental Sciences (K Vohra PhD), University of Birmingham, Birmingham, UK; Rabigh Faculty of Medicine (Prof A Malik PhD), King Abdulaziz University, Jeddah, Saudi Arabia; Department of Electrical Engineering (I Malik PhD), Prince Sattam bin Abdulaziz University, Al Kharj, Saudi Arabia; Department of Medical Microbiology (L A Malinga PhD), University of Pretoria, Pretoria, South Africa; Department of Health Research (L A Malinga PhD), Ministry of Health, Pretoria, South Africa; Division of Immunology, Immunity to Infection and

Respiratory Medicine (A G Mathioudakis PhD), University of Manchester, Manchester, UK; North West Lung Centre (A G Mathioudakis PhD), Manchester University NHS Foundation Trust, Manchester, UK; Department of Social Medicine (R Mattiello PhD), Federal University of Rio Grande do Sul, Porto Alegre, Brazil; Department of Medical and Surgical Sciences and Advanced Technologies "GF Ingrassia" (A Maugeri PhD), University of Catania, Catania, Italy; Department of Public Health (T Mekene Meto MPH), Arba Minch University, Arba Minch, Ethiopia; Division of Forensic Medicine (Prof R G Menezes MD), Imam Abdulrahman Bin Faisal University, Dammam, Saudi Arabia; Department of Physiology (Prof S A Meo PhD), Pediatric Intensive Care Unit (Prof M Temsah MD), King Saud University, Riyadh, Saudi Arabia; Comprehensive Cancer Center (T J Meretoja MD), Helsinki University Hospital, Helsinki, Finland; University Centre Varazdin (T Mestrovic PhD), University North, Varazdin, Croatia; Northwestern University, Chicago, IL, USA (L Mhlanga PhD); South African Centre for Epidemiological Modelling and Analysis (SACEMA) (L Mhlanga PhD), Stellenbosch University, Cape Town, South Africa; Pacific Institute for Research & Evaluation, Beltsville, MD, USA (T R Miller PhD); School of Public Health (T R Miller PhD), Curtin University, Perth, WA, Australia; Department of Statistics and Econometrics (A Mirica PhD, A Otoiu PhD), Bucharest University of Economic Studies, Bucharest, Romania; Internal Medicine Programme (Prof E M Mirrakhimov PhD), Kyrgyz State Medical Academy, Bishkek, Kyrgyzstan; Department of Atherosclerosis and Coronary Heart Disease (Prof E M Mirrakhimov PhD), National Center of Cardiology and Internal Disease, Bishkek, Kyrgyzstan; Department of Hospital Administration (M Mirza MD), Department of Radiodiagnosis (P Singh MD), Department of Community Medicine and Family Medicine (M Verma MD), All India Institute of Medical Sciences, Bathinda, India; National Data Management Center for Health (A Misganaw PhD), Ethiopian Public Health Institute, Addis Ababa, Ethiopia; College of Applied and Natural Science (J Mohamed MSc), University of Hargeisa, Hargeisa, Somalia; Molecular Biology Unit (N S Mohamed MSc), Bio-Statistical and Molecular Biology Department (N S Mohamed MSc), Sirius Training and Research Centre, Khartoum, Sudan; Health Systems and Policy Research Unit (Prof S Mohammed PhD), Ahmadu Bello University, Zaria, Nigeria; Heidelberg Institute of Global Health (HIGH) (Prof S Mohammed PhD), Heidelberg University, Heidelberg, Germany; Department of Mathematics (Prof S Momani PhD), The University of Jordan, Amman, Jordan; Department of Physiology (H Mondal MD), All India Institute of Medical Sciences, Deoghar, India; International Laboratory for Air Quality and Health (Prof L Morawska PhD), Queensland University of Technology, Brisbane, QLD, Australia; Unit of Pharmacotherapy, Epidemiology and Economics (Prof S Mubarik PhD), University of Groningen (Rijksuniversiteit Groningen), Groningen, Netherlands; Department of Epidemiology and Biostatistics (Prof S Mubarik PhD, Prof C Yu PhD), Wuhan University, Wuhan, China; Department of Community and Global Health (Y Munkhsaikhan MD), The University of Tokyo, Tokyo, Japan; Department of Psychiatry (W Myung PhD), Seoul National University, Seoul, South Korea; Department of Neuropsychiatry (W Myung PhD), Seoul National University Bundang Hospital, Seongnam, South Korea; Department of Pulmonary Medicine (Prof S Nair MD), Government Medical College, Thrissur, Thrissur, India; Health Action by People, Trivandrum, India (Prof S Nair MD); Suraj Eye Institute, Nagpur, India (V Nangia MD); Department of Biotechnology (M Naveed PhD), University of Central Punjab, Lahore, Pakistan; Xiamen Cardiovascular Hospital of Xiamen University (N PhD), Fujian Branch of National Clinical Research Center for Cardiovascular Diseases, Xiamen, China; Department of Disease Control and Environmental Health (R Ndejjo PhD), Makerere University, Kampala, Uganda; Harvard T.H. Chan School of Public Health (D Nguyen BS), Harvard University, Cambridge, MA, USA; Department of Medical Engineering (D Nguyen BS), University of South Florida, Tampa, FL, USA; Cardiovascular Research Department (H Q Nguyen MD), Methodist Hospital, Merrillville, IL, USA;

Tuberculosis Group (V T Nguyen MD), Oxford University Clinical Research Unit, Vietnam, Ho Chi Minh City, Vietnam; Department of General Surgery (T K Nikolouzakis PhD), University Hospital of Heraklion, Heraklion, Greece; Laboratory of Toxicology (T K Nikolouzakis PhD), Department of Medicine (Prof A Tsatsakis DSc), University of Crete, Heraklion, Greece; School of Medicine, University of Limerick (V Niranjan PhD), University of Limerick, Limerick, Ireland; Department of Public Health (V Niranjan PhD), UNICAF, Larnaca, Cyprus; Department of Statistics (S Noor MS), Shahjalal University of Science and Technology, Sylhet, Bangladesh; Environmental Health Research Center (A Norouzian Baghani PhD), Lorestan University of Medical Sciences, Khorramabad, Iran; Division of Cardiology (J Noubiap MD), University of California San Francisco, San Francisco, CA, USA; Department of Physiology (O J Nzoputam PhD), University of Benin, Edo, Nigeria; Department of Physiology (O J Nzoputam PhD), Benson Idahosa University, Benin City, Nigeria; Department of Applied Economics and Quantitative Analysis (Prof B Oancea PhD), University of Bucharest, Bucharest, Romania; Bioinformatics Department (Prof B Oancea PhD), National Institute of Research and Development for Biological Sciences, Bucharest, Romania; Department of Veterinary Public Health and Preventive Medicine (I A Odetokun PhD), University of Ilorin, Ilorin, Nigeria; Department of Public Health (D B Odo MPH), Arsi University, Asella, Ethiopia; Department of Physiology (O B Oghenetega PhD), Babcock University, Ilisan-Remo, Nigeria; School of Pharmacy (O C Okonji MSc), University of the Western Cape, Cape Town, South Africa; Department of Psychiatry (Prof A T Olagunju PhD), University of Lagos, Lagos, Nigeria; Federal Institute of Industrial Research Oshodi, Lagos, Nigeria (T A Olasehinde PhD); Department of Biochemistry, Genetics and Microbiology (T A Olasehinde PhD), University of KwaZulu-Natal, Westville, South Africa; Slum and Rural Health Initiative Research Academy (I I Olufadewa MHS), Slum and Rural Health Initiative, Ibadan, Nigeria; Department of Anatomy (G O Oluwatunase MSc), Olabisi Onabanjo University, Sagamu, Nigeria; Diplomacy and Public Relations Department (A Omar Bali PhD), University of Human Development, Sulaymaniyah, Iraq; College of Animal Science and Technology (M Ommati PhD), Henan University of Science and Technology, Henan, China; Food Security and Safety Niche Area (Prof A O Omotayo PhD), North-West University, Mafikeng, South Africa; Department of Environmental Biology and Health (M A Ondayo PhD), Department of Environmental Health and Biology (M A Ondayo PhD), University of Eldoret, Eldoret, Kenya; Department of Medicine (Prof M O Owolabi DrM), Department of Oral and Maxillofacial Surgery (A A Salami BDS), University College Hospital, Ibadan, Ibadan, Nigeria; Department of Respiratory Medicine (Prof M P P A DNB), Jagadguru Sri Shivarathreeswara University, Mysore, India; Department of Emergency Medicine (Prof I Pantazopoulos PhD), University of Thessaly, Larissa, Greece; Department of Emergency Medicine (Prof I Pantazopoulos PhD), University of Bern, Bern, Switzerland; Vision and Eye Research Institute (Prof S Pardhan PhD), Anglia Ruskin University, Cambridge, UK; Department of Community Medicine (P P Parija MD), All India Institute of Medical Sciences, Jammu, India; Division of Health Policy and Management (R R Parikh MD), University of Minnesota, Minneapolis, MN, USA; Department of Medical Humanities and Social Medicine (Prof E Park PhD), Kosin University, Busan, South Korea; Center for Pharmacoepidemiology and Treatment Science (A Parthasarathi MD), Rutgers University, New Brunswick, NJ, USA; Research Center (A Parthasarathi MD), Allergy Asthma and Chest Center, Mysore, India; Global Health Governance Programme (J Patel BSc), Centre for Medical Informatics (Prof A Sheikh MD), University of Edinburgh, Edinburgh, UK; Natnov Bioscience, Odisha, India (S Pati PhD); Department of Genetics (S Pawar PhD), Yale University, New Haven, CT, USA; Australian Institute of Health Innovation (P Peprah MSc), Macquarie University, Sydney, NSW, Australia; School of Population Health (Prof G Pereira PhD), Curtin University, Bentley, WA, Australia; Centre for Fertility and Health (Prof G Pereira PhD), Department of Chemical Toxicology (M W Wojewodzic PhD),

Norwegian Institute of Public Health, Oslo, Norway; Department of Internal Medicine (H Pham MD), Weiss Memorial Hospital, Chicago, IL, USA; Department of Data Management and Analysis (R Poluru PhD), The International Clinical Epidemiology Network (INCLEN) Trust International, New Delhi, India; Department of Humanities and Social Sciences (Prof J Pradhan PhD), National Institute of Technology Rourkela, Rourkela, India; Department of Maternal-Child Nursing and Public Health (E J S Prates BS), Federal University of Minas Gerais, Belo Horizonte, Brazil; Health Sciences Department (D R A Pribadi MSc), Muhammadiyah University of Surakarta, Sukoharjo, Indonesia; Department of Biostatistics, Epidemiology, and Informatics (J Puvvula PhD), University of Pennsylvania, Philadelphia, PA, USA; Department of Medicine (A Rafiee MSc), University of Alberta, Edmonton, AB, Canada; Osh State University, Osh, Kyrgyzstan (Prof F Rahim PhD); College of Medicine and Health Sciences (M Rahman PhD), National University of Science and Technology, Sohar, Oman; Department of Population Science and Human Resource Development (Prof M Rahman DrPH), University of Rajshahi, Rajshahi, Bangladesh; Institute of Health and Wellbeing (Prof M Rahman PhD), Federation University Australia, Berwick, VIC, Australia; School of Nursing and Midwifery (Prof M Rahman PhD), La Trobe University, Melbourne, VIC, Australia; Future Technology Research Center (A Rahmani PhD), National Yunlin University of Science and Technology, Yunlin, Taiwan; Department of Community Medicine (S Rajaa MD), Employees' State Insurance Model Hospital, Chennai, India; Centre for Chronic Disease Control, New Delhi, India (P Rajput PhD); Department of Cardiology (Prof M M Ramadan PhD), Mansoura University, Mansoura, Egypt; South Asian Institute for Social Transformation (SAIST), Dhaka, Bangladesh (J Rana MPH); Department of Epidemiology, Biostatistics and Occupational Health (J Rana MPH), McGill University, Montreal, QC, Canada; Translational Health Research Institute (K Rana PhD), Western Sydney University, Sydney, NSW, Australia; Department of Research (C L Ranabhat PhD), Eastern Scientific LLC, Richmond, KY, USA; Planetary Health Research Centre (PHRC), Kathmandu, Nepal (C L Ranabhat PhD); Data Analytic Services (D P Rasali PhD), British Columbia Centre for Disease Control, Vancouver, BC, Canada; School of Population and Public Health (D P Rasali PhD, Prof M Brauer DSc), University of British Columbia, Vancouver, BC, Canada; Department of Global Health Policy (S K Rauniyar PhD), University of Tokyo, Tokyo, Japan; Academic Public Health England (Prof S Rawaf MD), Public Health England, London, UK; Department of Biological Sciences (Prof E M M Redwan PhD), King Abdulaziz University, Jeddah, Egypt; Department of Protein Research (Prof E M M Redwan PhD), Research and Academic Institution, Alexandria, Egypt; Department of Pharmacology and Toxicology (Prof J A B Rodriguez PhD), University of Antioquia, Medellin, Colombia; Warwick Medical School (Prof J A B Rodriguez PhD), University of Warwick, Coventry, UK; School of Psychology (S Röhr PhD), Massey University, Auckland, New Zealand; Centre for Healthy Brain Ageing (CHeBA) (S Röhr PhD), School of Population Health (Prof B A Saddik PhD), University of New South Wales, Sydney, NSW, Australia; Golestan Research Center of Gastroenterology and Hepatology (G Roshandel PhD), Golestan University of Medical Sciences, Gorgan, Iran; Department of Analytical and Applied Economics (Prof H Rout PhD, C Swain MPhil), RUSA Centre of Excellence in Public Policy and Governance (Prof H Rout PhD), Department of Analytical & Applied Economics (P Sahoo MA), UGC Centre of Advanced Study in Psychology (Prof M Satpathy PhD), Utkal University, Bhubaneswar, India; Department of Labour (P Roy PhD), Directorate of Factories, Government of West Bengal, Kolkata, India; Department of Cardiology (M Russo PhD), S. Maria dei Battuti Hospital, Conegliano, Italy; Department of Medicine (C J Sabet MA), Georgetown University, Washington, DC, USA; Operational Research Center in Healthcare (Prof U Saeed PhD), Near East University (NEU), Nicosia Cyprus, Turkiye; International Center of Medical Sciences Research (ICMSR), Islamabad, Pakistan (Prof U Saeed PhD); Center for Global Health Research (Prof A Sahebkar PhD),

Saveetha Dental College and Hospitals (M Tovani-Palone PhD), Saveetha University, Chennai, India; Biotechnology Research Center (Prof A Sahebkar PhD), Department of Medical Informatics (S Tabatabaei PhD), Clinical Research Development Unit (S Tabatabaei PhD), Mashhad University of Medical Sciences, Mashhad, Iran; College of Nursing (D Salihu PhD), Jouf University, Jouf, Saudi Arabia; Department of Entomology (A M Samy PhD), Medical Ain Shams Research Institute (MASRI) (A M Samy PhD), Ain Shams University, Cairo, Egypt; Faculty of Medicine (Prof M M Santric-Milicevic PhD), School of Public Health and Health Management (Prof M M Santric-Milicevic PhD), University of Belgrade, Belgrade, Serbia; Department of Food Processing Technology (T Sarkar PhD), West Bengal State Council of Technical Education, Malda, India; Udyam-Global Association for Sustainable Development, Bhubaneswar, India (Prof M Satpathy PhD); Department of Preventive and Social Medicine (G Saya MD), Jawaharlal Institute of Postgraduate Medical Education and Research, Puducherry, India; National Centre for Epidemiology and Population Health (M Sayeed MS), Australian National University, Acton, ACT, Australia; Department of Medicine (Y Sethi MD), Swami Vivekanand Subharti University, Meerut, India; National Heart, Lung, and Blood Institute (A Seylani MD), National Institutes of Health, Rockville, MD, USA; Institute of Molecular Biology and Biotechnology (S Shahid PhD), Research Centre for Health Sciences (RCHS) (S Shahid PhD), The University of Lahore, Lahore, Pakistan; Department of Pathology and Laboratory Medicine (S Sham MD), Northwell Health, New York, NY, USA; Centre For Interdisciplinary Research In Basic Sciences (CIRBSc) (A Shamsi PhD), Jamia Millia Islamia, New Delhi, India; Oulu Business School (I Shiue PhD), Martti Ahtisaari Institute (I Shiue PhD), University of Oulu, Oulu, Finland; Department of Medical Microbiology and Infectious Diseases (E E Siddig MD), Erasmus University, Rotterdam, Netherlands; Occupational and Environmental Medicine Department (L Stockfelt PhD), University of Gothenburg, Gothenburg, Sweden; Global Observatory on Pollution and Health (Prof K Straif PhD), Boston College, Chestnut Hill, MA, USA; ISGlobal Instituto de Salud Global de Barcelona, Barcelona, Spain (Prof K Straif PhD); Department of Foundational Science (B Swami Vetha PhD), East Carolina University, Greenville, NC, USA; Department of Dermatology (M Tampa PhD), Carol Davila University of Medicine and Pharmacy, Bucharest, Romania; Department of Dermato-Venereology (M Tampa PhD), Dr. Victor Babes Clinical Hospital of Infectious Diseases and Tropical Diseases, Bucharest, Romania; State Key Laboratory of Numerical Modeling for Atmospheric Sciences and Geophysical Fluid Dynamics (LASG) (H Tang PhD), Chinese Academy of Sciences, Beijing, China; Department of Radiology (M Tanwar MD), University of Alabama at Birmingham, Birmingham, AL, USA; School of Nursing and Public Health (Prof E E Tarkang PhD), University of KwaZulu-Natal, Durban, South Africa; Department of Public Health (Y M Tefera MPH), Dire Dawa University, Dire Dawa, Ethiopia; School of Humanities and Social Sciences (R Thakur PhD), Indian Institute of Technology Mandi, Mandi, India; Department of Medicine (Prof F Thienemann PhD), University of Cape Town, Cape Town, South Africa; Department of Internal Medicine (Prof F Thienemann PhD), University of Zürich, Zurich, Switzerland; Department of Community Medicine and Family Medicine (J P Tripathy MD), All India Institute of Medical Sciences, Nagpur, India; Department of Internal Medicine (M Tumurkhuu PhD), Wake Forest University, Winston-Salem, NC, USA; Department of Zoology (S Ullah PhD), Division of Science and Technology (S Ullah PhD), University of Education Lahore, Lahore, Pakistan; Department of Infectious Disease (Prof S Vaziri MD), Kermanshah University of Medical Sciences, Kermanshah, Iran; Department of Neurology (S Vidale MD), Infermi Hospital, Rimini, Italy; Department of Neurology & Stroke Unit (S Vidale MD), Sant'Anna Hospital, Como, Italy; Department of Community Medicine (N D Wickramasinghe MD), Rajarata University of Sri Lanka, Anuradhapura, Sri Lanka; Department of Research (M W Wojewodzic PhD), Cancer Registry of Norway, Oslo, Norway; Department of Public Health (T E Wonde MPH), Debre Markos University, Debre Markos,

Ethiopia; Department of Food Science and Human Nutrition (Prof F Wu PhD), Michigan State University, East Lansing, MI, USA; School of Public Health (H Xiao PhD), Zhejiang University, Zhejiang, China; Department of Public Health Science (H Xiao PhD), Fred Hutchinson Cancer Research Center, Seattle, WA, USA; Department of Endocrinology (Prof S Xu PhD), University of Science and Technology of China, Hefei, China; School of Medicine (Prof S Xu PhD), University of Rochester, Rochester, NY, USA; Department of Microbiology (M Yadav PhD), Central University of Punjab, Bathinda, India; Department of Community Medicine (S Yahoo Syed MD), Apollo Hospital, Hyderabad, India; Department of Health Management (A Yiğit PhD, V Yiğit PhD), Süleyman Demirel Üniversitesi (Süleyman Demirel University), Isparta, Turkiye; Department of Pediatrics (Prof D Yon MD), Kyung Hee University, Seoul, South Korea; Department of Biostatistics (Prof N Yonemoto PhD), University of Toyama, Toyama, Japan; Department of Public Health (Prof N Yonemoto PhD), Juntendo University, Tokyo, Japan; Department of Parasitology and Entomology (L Zaki PhD), Tarbiat Modares University, Tehran, Iran; Department of Anesthesiology (Y Zeng MD), Third Xiangya Hospital of Central South University, Changsha, China; Department of Public Health (C Zhai MPH), Nantong Sixth People's Hospital (Nantong Hospital Affiliated to Shanghai University), Nantong, China; School of Public Health (H Zhang MS), Peking University, Beijing, China; Tianjin Medical University General Hospital (Z Zhang MD), Tianjin Centers for Disease Control and Prevention, Tianjin, China; School of Public Health and Emergency Management (B Zhu PhD), Southern University of Science and Technology, Shenzhen, China; Department of Clinical and Community Pharmacy (Prof S H Zyoud PhD), An-Najah National University, Nablus, Palestine; Clinical Research Centre (Prof S H Zyoud PhD), An-Najah National University Hospital, Nablus, Palestine

Authors’ Contributions

Managing the overall research enterprise

Charlie Ashbaugh, Michael Brauer, and Katrin Burkart.

Writing the first draft of the manuscript

Fiona Bennitt, Michael Brauer, Katrin Burkart, and Sarah Wozniak.

Primary responsibility for applying analytical methods to produce estimates

Fiona Bennitt, Michael Brauer, Katrin Burkart, Kate Causey, Nadim Hashme, Sandra Spearman, and Sarah Wozniak.

Primary responsibility for seeking, cataloguing, extracting, or cleaning data; designing or coding figures and tables

Fiona Bennitt, Michael Brauer, Katrin Burkart, Kate Causey, Vanessa Garcia, Chukwuma Okereke, Sandra Spearman, and Sarah Wozniak.

Providing data or critical feedback on data sources

Armita Abedi, Roberto Ariel Abeldaño Zuñiga, Richard Gyan Aboagye, Ahmed Abu-Zaid, Victor Adekanmbi, Muhammad Sohail Afzal, Saira Afzal, Danish Ahmad, Sajjad Ahmad, Ali Ahmadi, Ayman Ahmed, Haroon Ahmed, Rufus Olusola Akinyemi, Salah Al Awaidy, Hanadi Al Hamad, Mulubirhan Assefa Alemayohu, Abid Ali, Waad Ali, Sheikh Mohammad Alif, Sami Almustanyir, Nelson Alvis-Guzman, Hubert Amu, Tadele Fentabel Anagaw, Saeid Anvari, Ekenedilichukwu Emmanuel Anyabolo, Geminn Louis Carace Apostol, Olatunde Aremu, Akeza Awealom Asgedom, Tahira Ashraf, Seyyed Shamsadin Athari, Ahmed Y Azzam, Giridhara Rathnaiah Babu, Maciej Banach, Biswajit Banik, Mehmet Firat Baran, Mohammad-Mahdi Bastan, Sanjay Basu, Melesse Belayneh, Apostolos Beloukas, Fiona B Bennitt,

Devidas S Bhagat, Sonu Bhaskar, Ajay Nagesh Bhat, Priyadarshini Bhattacharjee, Gurjit Kaur Bhatti, Cem Bilgin, Sri Harsha Boppana, Michael Brauer, Katrin Burkart, Fan Cao, Kate Causey, Gashaw Sisay Chanie, Vijay Kumar Chattu, Akhilanand Chaurasia, Guangjin Chen, Ritesh Chimoriya, Bryan Chong, Devasahayam J Christopher, Aaron J Cohen, Natalia Cruz-Martins, Xiaochen Dai, Samuel Demissie Darcho, Saswati Das, Meghnath Dhimal, Thanh Chi Do, Ojas Prakashbhai Doshi, Aziz Eftekharimehrabad, Temitope Cyrus Ekundayo, Ibrahim Farahat El Bayoumy, Syed Emdadul Haque, Habtamu Demelash Enyew, Ayesha Fahim, Alireza Feizkhah, Morenike Oluwatoyin Folayan, Sridevi G, Muktar A Gadanya, Teferi Gebru Gebremeskel, Mahaveer Golechha, Davide Golinelli, Shi-Yang Guan, Demisu Zenbaba Heyi, Nguyen Quoc Hoan, Chengxi Hu, Hong-Han Huynh, Segun Emmanuel Ibitoye, Ammar Abdulrahman Jairoun, Abhishek Jaiswal, Mihajlo Jakovljevic, Shubha Jayaram, Jost B Jonas, Vidya Kadashetti, Gbenga A Kayode, Himanshu Khajuria, Khaled Khatab, Atulya Aman Khosla, Majid Khosravi, Shivakumar KM, Luke D Knibbs, Gerbrand Koren, Parvaiz A Koul, Kewal Krishan, Barthelemy Kuate Defo, Chandrakant Lahariya, Nhi Huu Hanh Le, Munjae Lee, Seung Won Lee, Stephen S Lim, Gang Liu, Shuke Liu, Wei Liu, Jay B Lusk, Sandeep B Maharaj, Kashish Malhotra, Alexander G Mathioudakis, Rita Mattiello, Andrea Maugeri, Tesfahun Mekene Meto, Ritesh G Menezes, Ted R Miller, Erkin M Mirrakhimov, Awoke Misganaw, Nouh Saad Mohamed, Abdollah Mohammadian-Hafshejani, Mustapha Mohammed, Shafiu Mohammed, Ali H Mokdad, Rohith Motappa, Sumaira Mubarik, Yanjinlkham Munkhsaikhan, Christopher J L Murray, Sanjeev Nair, Muhammad Naveed, Dang Nguyen, Hien Quang Nguyen, Van Thanh Nguyen, Taxiarchis Konstantinos Nikolouzakis, Syed Toukir Ahmed Noor, Jean Jacques Noubiap, Bogdan Oancea, Ismail A Odetokun, Onome Bright Oghenetega, Andrew T Olagunju, Gideon Olamilekan Oluwatunase, Ahmed Omar Bali, Mohammad Mehdi Ommati, Abiodun Olusola Omotayo, Maureene Auma Ondayo, Mayowa O Owolabi, Mahesh Padukudru P A, Jagadish Rao Padubidri, Romil R Parikh, Siddhartha Pati, Shrikant Pawar, Prince Peprah, Gavin Pereira, Arokiasamy Perianayagam, Hoang Tran Pham, Ramesh Poluru, Jalandhar Pradhan, Elton Junio Sady Prates, Dimas Ria Angga Pribadi, Jagadeesh Puvvula, Pankaja Raghav, Amir Masoud Rahmani, Sathish Rajaa, Mahmoud Mohammed Ramadan, Juwel Rana, Kritika Rana, Chhabi Lal Ranabhat, Santosh Kumar Rauniyar, Salman Rawaf, Jefferson Antonio Buendia Rodriguez, Priyanka Roy, Cameron John Sabet, Basema Ahmad Saddik, Umar Saeed, Pragyan Monalisa Sahoo, Afeez Abolarinwa Salami, Abdallah M Samy, Milena M Santric-Milicevic, Maheswar Satpathy, Yashendra Sethi, Samiah Shahid, Sunder Sham, Muhammad Aaqib Shamim, Anas Shamsi, Aminu Shittu, Ivy Shiue, Paramdeep Singh, Chandan Kumar Swain, Seyyed Mohammad Tabatabaei, Mircea Tampa, Manoj Tanwar, Elvis Enowbeyang Tarkang, Yibekal Manaye Tefera, Friedrich Thienemann, Krishna Tiwari, Marcos Roberto Tovani-Palone, Munkhtuya Tumurkhuu, Sana Ullah, Sanaz Vahdati, Theo Vos, Tewodros Eshete Wonde, Sarah Wozniak, Felicia Wu, Hong Xiao, Suowen Xu, Sanni Yaya, Arzu Yiğit, Dong Keon Yon, Naohiro Yonemoto, Chuanhua Yu, Chunxia Zhai, and Sa'ed H Zyoud.

Developing methods or computational machinery

Aleksandr Y Aravkin, Michael Brauer, Xiaochen Dai, Ali H Mokdad, Christopher J L Murray, Austin E Schumacher, and Theo Vos.

Providing critical feedback on methods or results

Atef Abdelkader, Meriem Abdoun, Muhammed Jemal Abdurebi, Armita Abedi, Roberto Ariel Abeldaño Zuñiga, Richard Gyan Aboagye, Bilyaminu Abubakar, Ahmed Abu-Zaid, Mesafint Molla Adane, Oyelola A Adegboye, Victor Adekanmbi, Abiola Victor Adepoju, Temitayo Esther Adeyeoluwa, Rishan Adha, Muhammad Sohail Afzal, Saira Afzal, Feleke Doyore Agide, Aqeel Ahmad, Danish Ahmad, Muayyad M Ahmad, Sajjad Ahmad, Ali Ahmadi, Anisuddin Ahmed, Ayman Ahmed, Haroon Ahmed, Rufus Olusola

Akinyemi, Salah Al Awaidy, Hanadi Al Hamad, Muaaz M Alajlani, Mulubirhan Assefa Alemayohu, Adel Ali Saeed Al-Gheethi, Abid Ali, Waad Ali, Sheikh Mohammad Alif, Sami Almustanyir, Nelson Alvis-Guzman, Nelson J Alvis-Zakzuk, Hany Aly, Hubert Amu, Ganiyu Adeniyi Amusa, Tadele Fentabel Anagaw, Boluwatife Stephen Anuoluwa, Iyadunni Adesola Anuoluwa, Saeid Anvari, Ekenedilichukwu Emmanuel Anyabolo, Geminn Louis Carace Apostol, Demelash Areda, Brhane Berhe Aregawi, Olatunde Aremu, Akeza Awealom Asgedom, Mubarek Yesse Ashemo, Tahira Ashraf, Seyyed Shamsadin Athari, Sina Azadnajafabad, Ahmed Y Azzam, Giridhara Rathnaiah Babu, Saeed Bahramian, Maciej Banach, Biswajit Banik, Mehmet Firat Baran, Sandra Barteit, Hameed Akande Bashiru, Pritish Baskaran, Mohammad-Mahdi Bastan, Sanjay Basu, Saurav Basu, Sefealem Assefa Belay, Melesse Belayneh, Apostolos Beloukas, Derrick A Bennett, Fiona B Bennitt, Devidas S Bhagat, Dinesh Bhandari, Pankaj Bhardwaj, Sonu Bhaskar, Ajay Nagesh Bhat, Priyadarshini Bhattacharjee, Gurjit Kaur Bhatti, Manpreet S Singh Bhatti, Cem Bilgin, Sri Harsha Boppana, Samuel Adolf Bosoka, Michael Brauer, Katrin Burkart, Fan Cao, Kate Causey, Gashaw Sisay Chanie, Vijay Kumar Chattu, Akhilanand Chaurasia, Guangjin Chen, Ritesh Chimoriya, Bryan Chong, Devasahayam J Christopher, Isaac Sunday Chukwu, Aaron J Cohen, Natalia Cruz-Martins, Omid Dadras, Xiaochen Dai, Patience Unekwuojo Daikwo, Samuel Demissie Darcho, Saswati Das, Belay Desye, Sagnik Dey, Meghnath Dhimal, Daniel Diaz, Thanh Chi Do, Ojas Prakashbhai Doshi, Abdel Rahman E'mar, Alireza Ebrahimi, Hisham Atan Edinur, Aziz Eftekharimehrabad, Temitope Cyrus Ekundayo, Ibrahim Farahat El Bayoumy, Syed Emdadul Haque, Theophilus I Emeto, Habtamu Demelash Enyew, Ayesha Fahim, Adekunle Gregory Fakunle, Sasan Faridi, Timur Fazylov, Alireza Feizkhah, Florian Fischer, Sridevi G, Muktar A Gadanya, Xiang Gao, Vanessa Garcia, Miglas Welay Gebregergis, Mesfin Gebrehiwot, Teferi Gebru Gebremeskel, Afsaneh Ghasemzadeh, Nermin Ghith, Mahaveer Golechha, Shi-Yang Guan, Zhifeng Guo, Bhawna Gupta, Lalit Gupta, Rabih Halwani, Ahmed I Hasaballah, Md Saquib Hasnain, Simon I Hay, Demisu Zenbaba Heyi, Kamal Hezam, Nguyen Quoc Hoan, Ramesh Holla, Hassan Hosseinzadeh, Chengxi Hu, Hong-Han Huynh, Bing-Fang Hwang, Segun Emmanuel Ibitoye, Oluwatope Olaniyi Idowu, Mustapha Immurana, Arit Inok, Sheikh Mohammed Shariful Islam, Vinothini J, Ammar Abdulrahman Jairoun, Abhishek Jaiswal, Mihajlo Jakovljevic, Reza Jalilzadeh Yengejeh, Shubha Jayaram, Alelign Tasew Jema, Ravi Prakash Jha, Jost B Jonas, Nitin Joseph, Vidya Kadashetti, Kehinde Kazeem Kanmodi, Sushil Kumar Kansal, Ibraheem M Karaye, Gbenga A Kayode, Himanshu Khajuria, Amirmohammad Khalaji, Vishnu Khanal, Khaled Khatab, Khalid A Kheirallah, Atulya Aman Khosla, Majid Khosravi, Shivakumar KM, Gerbrand Koren, Parvaiz A Koul, Kewal Krishan, Barthelemy Kuate Defo, Mohammed Kuddus, Ashish Kumar, Dewesh Kumar, Nithin Kumar, Om P Kurmi, Chandrakant Lahariya, Hanpeng Lai, Tuo Lan, Paolo Lauriola, Nhi Huu Hanh Le, Munjae Lee, Seung Won Lee, Stephen S Lim, Gang Liu, Wei Liu, José Francisco López-Gil, Kashish Malhotra, Ahmad Azam Malik, Iram Malik, Alexander G Mathioudakis, Rita Mattiello, Andrea Maugeri, Tesfahun Mekene Meto, Hadush Negash Meles, Ritesh G Menezes, Sultan Ayoub Meo, Seid Tiku Mereta, Tomislav Mestrovic, Laurette Mhlanga, Ted R Miller, Andreea Mirica, Erkin M Mirrakhimov, Moonis Mirza, Prasanna Mithra, Jama Mohamed, Nouh Saad Mohamed, Abdollah Mohammadian-Hafshejani, Mustapha Mohammed, Shafiu Mohammed, Ali H Mokdad, Shaher Momani, Himel Mondal, Lidia Morawska, Rohith Motappa, Sumaira Mubarik, Kavita Munjal, Yanjinlkham Munkhsaikhan, Christopher J L Murray, Sanjeev Nair, Vinay Nangia, Muhammad Naveed, Nawsherwan, Rawlance Ndejjo, Dang Nguyen, Hien Quang Nguyen, Van Thanh Nguyen, Taxiarchis Konstantinos Nikolouzakis, Vikram Niranjan, Efaq Ali Noman, Syed Toukir Ahmed Noor, Jean Jacques Noubiap, Ogochukwu Janet Nzoputam, Bogdan Oancea, Ismail A Odetokun, Akinyemi O D Ofakunrin, Onome Bright Oghenetega, Osaretin Christabel Okonji, Andrew T Olagunju, Tosin Abiola Olasehinde, Isaac Iyinoluwa Olufadewa, Gideon Olamilekan Oluwatunase, Ahmed Omar Bali, Mohammad Mehdi Ommati,

Abiodun Olusola Omotayo, Maureene Auma Ondayo, Adrian Otoiu, Mayowa O Owolabi, Mahesh Padukudru P A, Jagadish Rao Padubidri, Ioannis Pantazopoulos, Shahina Pardhan, Pragyan Paramita Parija, Romil R Parikh, Eun-Kee Park, Ashwaghosha Parthasarathi, Jay Patel, Shrikant Pawar, Prince Peprah, Gavin Pereira, Hoang Tran Pham, Ramesh Poluru, Akram Pourshams, Jalandhar Pradhan, Elton Junio Sady Prates, Jagadeesh Puvvula, Pankaja Raghav, Fakher Rahim, Mohammad Hifz Ur Rahman, Muhammad Aziz Rahman, Amir Masoud Rahmani, Mohammad Rahmanian, Sathish Rajaa, Prashant Rajput, Mahmoud Mohammed Ramadan, Juwel Rana, Kritika Rana, Drona Prakash Rasali, Santosh Kumar Rauniyar, Salman Rawaf, Elrashdy M Moustafa Mohamed Redwan, Nazila Rezaei, Jefferson Antonio Buendia Rodriguez, Susanne Röhr, Gholamreza Roshandel, Himanshu Sekhar Rout, Priyanka Roy, Michele Russo, Cameron John Sabet, Basema Ahmad Saddik, Umar Saeed, Narjes Saheb Sharif-Askari, Pragyan Monalisa Sahoo, Afeez Abolarinwa Salami, Abdallah M Samy, Milena M Santric-Milicevic, Tanmay Sarkar, Maheswar Satpathy, Ganesh Kumar Saya, Md Abu Sayeed, Mihretu Tagesse Sergindo, Yashendra Sethi, Samiah Shahid, Muhammad Aaqib Shamim, Anas Shamsi, Aziz Sheikh, Aminu Shittu, Ivy Shiue, Emmanuel Edwar Siddig, Paramdeep Singh, Md Shahjahan Siraj, Kurt Straif, Chandan Kumar Swain, Berwin Singh Swami Vetha, Seyyed Mohammad Tabatabaei, Mircea Tampa, Haosu Tang, Manoj Tanwar, Elvis Enowbeyang Tarkang, Yibekal Manaye Tefera, Mohamad-Hani Temsah, Ramna Thakur, Friedrich Thienemann, Nigusie Selomon Tibebu, Krishna Tiwari, Marcos Roberto Tovani-Palone, Jaya Prasad Tripathy, Munkhtuya Tumurkhuu, Aniefiok John Udoakang, Sana Ullah, Sanaz Vahdati, Siavash Vaziri, Madhur Verma, Simona Villani, Karn Vohra, Theo Vos, Gizachew Tadesse Wassie, Haftom Legese Weldetinsaa, Adhena Ayaliew Werkneh, Nuwan Darshana Wickramasinghe, Tewodros Eshete Wonde, Sarah Wozniak, Felicia Wu, Zenghong Wu, Hong Xiao, Suowen Xu, Mukesh Kumar Yadav, Saba Yahoo Syed, Sanni Yaya, Arzu Yiğit, Vahit Yiğit, Dehui Yin, Dong Keon Yon, Naohiro Yonemoto, Leila Zaki, Mohammed G M Zeariya, Youjie Zeng, Chunxia Zhai, Haijun Zhang, Zhiqiang Zhang, Bin Zhu, and Sa'ed H Zyoud.

Drafting the work or revising it critically for important intellectual content

Atef Abdelkader, Muhammed Jemal Abdurebi, Armita Abedi, Roberto Ariel Abeldaño Zuñiga, Bilyaminu Abubakar, Ahmed Abu-Zaid, Mesafint Molla Adane, Oyelola A Adegboye, Victor Adekanmbi, Abiola Victor Adepoju, Olorunsola Israel Adeyomoye, Muhammad Sohail Afzal, Saira Afzal, Feleke Doyore Agide, Danish Ahmad, Muayyad M Ahmad, Ali Ahmadi, Sepideh Ahmadi, Anisuddin Ahmed, Ayman Ahmed, Haroon Ahmed, Marjan Ajami, Rufus Olusola Akinyemi, Muaaz M Alajlani, Mulubirhan Assefa Alemayohu, Abid Ali, Waad Ali, Sami Almustanyir, Nelson Alvis-Guzman, Nelson J Alvis-Zakzuk, Hany Aly, Hubert Amu, Ganiyu Adeniyi Amusa, Tadele Fentabel Anagaw, Boluwatife Stephen Anuoluwa, Iyadunni Adesola Anuoluwa, Saeid Anvari, Geminn Louis Carace Apostol, Olatunde Aremu, Akeza Awealom Asgedom, Seyyed Shamsadin Athari, Sina Azadnajafabad, Ahmed Y Azzam, Giridhara Rathnaiah Babu, Kiran Bam, Maciej Banach, Mehmet Firat Baran, Francesco Barone-Adesi, Hameed Akande Bashiru, Pritish Baskaran, Mohammad-Mahdi Bastan, Sanjay Basu, Melesse Belayneh, Apostolos Beloukas, Fiona B Bennitt, Dinesh Bhandari, Sonu Bhaskar, Ajay Nagesh Bhat, Priyadarshini Bhattacharjee, Gurjit Kaur Bhatti, Mary Sefa Boampong, Sri Harsha Boppana, Samuel Adolf Bosoka, Sofiane Boudalia, Michael Brauer, Katrin Burkart, Rama Mohan Chandika, Vijay Kumar Chattu, Anis Ahmad Chaudhary, Akhilanand Chaurasia, Guangjin Chen, Yifan Chen, Ritesh Chimoriya, Bryan Chong, Natalia Cruz-Martins, Patience Unekwuojo Daikwo, Samuel Demissie Darcho, Juana Maria Delgado-Saborit, Belay Desye, Sagnik Dey, Meghnath Dhimal, Daniel Diaz, Thanh Chi Do, Ojas Prakashbhai Doshi, Abdel Rahman E’mar, Aziz Eftekharimehrabad, Ibrahim Farahat El Bayoumy, Theophilus I Emeto, Ayesha Fahim, Adekunle Gregory Fakunle, Florian Fischer, Morenike Oluwatoyin Folayan, Sridevi G, Muktar A Gadanya, Vanessa Garcia,

Miglas Welay Gebregergis, Afsaneh Ghasemzadeh, Nermin Ghith, Mahaveer Golechha, Davide Golinelli, Shi-Yang Guan, Zhifeng Guo, Bhawna Gupta, Lalit Gupta, Rabih Halwani, Ahmed I Hasaballah, Md Saquib Hasnain, Simon I Hay, Kamal Hezam, Nguyen Quoc Hoan, Ramesh Holla, Hong-Han Huynh, Segun Emmanuel Ibitoye, Adalia Ikiroma, Mustapha Immurana, Arit Inok, Muhammad Iqhrammullah, Rakibul M Islam, Sheikh Mohammed Shariful Islam, Abhishek Jaiswal, Mihajlo Jakovljevic, Manthan Dilipkumar Janodia, Shubha Jayaram, Alelign Tasew Jema, Ravi Prakash Jha, Jost B Jonas, Nitin Joseph, Vidya Kadashetti, Kehinde Kazeem Kanmodi, Sushil Kumar Kansal, Gbenga A Kayode, Himanshu Khajuria, Amirmohammad Khalaji, Vishnu Khanal, Khaled Khatab, Khalid A Kheirallah, Atulya Aman Khosla, Majid Khosravi, Shivakumar KM, Kewal Krishan, Barthelemy Kuate Defo, Mohammed Kuddus, Mukhtar Kulimbet, Vishnutheertha Kulkarni, Ashish Kumar, Dewesh Kumar, Om P Kurmi, Chandrakant Lahariya, Hanpeng Lai, Nhi Huu Hanh Le, Shuke Liu, Wei Liu, José Francisco López-Gil, Jay B Lusk, Kashish Malhotra, Ahmad Azam Malik, Lesibana Anthony Malinga, Alexander G Mathioudakis, Andrea Maugeri, Hadush Negash Meles, Ritesh G Menezes, Sultan Ayoub Meo, Tuomo J Meretoja, Tomislav Mestrovic, Ted R Miller, Moonis Mirza, Awoke Misganaw, Prasanna Mithra, Nouh Saad Mohamed, Abdollah Mohammadian-Hafshejani, Mustapha Mohammed, Shafiu Mohammed, Ali H Mokdad, Himel Mondal, Yanjinlkham Munkhsaikhan, Woojae Myung, Sanjeev Nair, Nawsherwan, Dang Nguyen, Hien Quang Nguyen, Van Thanh Nguyen, Taxiarchis Konstantinos Nikolouzakis, Abbas Norouzian Baghani, Jean Jacques Noubiap, Ogochukwu Janet Nzoputam, Bogdan Oancea, Ismail A Odetokun, Daniel Bogale Odo, Akinyemi O D Ofakunrin, Onome Bright Oghenetega, Osaretin Christabel Okonji, Andrew T Olagunju, Gideon Olamilekan Oluwatunase, Abiodun Olusola Omotayo, Maureene Auma Ondayo, Adrian Otoiu, Mayowa O Owolabi, Mahesh Padukudru P A, Jagadish Rao Padubidri, Ioannis Pantazopoulos, Shahina Pardhan, Romil R Parikh, Ashwaghosha Parthasarathi, Jay Patel, Siddhartha Pati, Shrikant Pawar, Gavin Pereira, Arokiasamy Perianayagam, Hoang Tran Pham, Jalandhar Pradhan, Elton Junio Sady Prates, Jagadeesh Puvvula, Ata Rafiee, Pankaja Raghav, Fakher Rahim, Mohammad Hifz Ur Rahman, Amir Masoud Rahmani, Mohammad Rahmanian, Sathish Rajaa, Rayan Rajabi, Prashant Rajput, Mahmoud Mohammed Ramadan, Kritika Rana, Salman Rawaf, Elrashdy M Moustafa Mohamed Redwan, Jefferson Antonio Buendia Rodriguez, Susanne Röhr, Gholamreza Roshandel, Michele Russo, Cameron John Sabet, Basema Ahmad Saddik, Umar Saeed, Amirhossein Sahebkar, Afeez Abolarinwa Salami, Dauda Salihu, Abdallah M Samy, Milena M Santric-Milicevic, Tanmay Sarkar, Maheswar Satpathy, Ganesh Kumar Saya, Md Abu Sayeed, Yashendra Sethi, Allen Seylani, Samiah Shahid, Muhammad Aaqib Shamim, Anas Shamsi, Pavanchand H Shetty, Aminu Shittu, Emmanuel Edwar Siddig, Paramdeep Singh, Surjit Singh, Leo Stockfelt, Mircea Tampa, Manoj Tanwar, Yibekal Manaye Tefera, Mohamad-Hani Temsah, Reem Mohamad Hani Temsah, Ramna Thakur, Friedrich Thienemann, Krishna Tiwari, Marcos Roberto Tovani-Palone, Jaya Prasad Tripathy, Aristidis Tsatsakis, Aniefiok John Udoakang, Sanaz Vahdati, Madhur Verma, Simone Vidale, Simona Villani, Nuwan Darshana Wickramasinghe, Marcin W Wojewodzic, Tewodros Eshete Wonde, Sarah Wozniak, Mukesh Kumar Yadav, Saba Yahoo Syed, Sanni Yaya, Arzu Yiğit, Vahit Yiğit, Dong Keon Yon, Naohiro Yonemoto, Mohammed G M Zeariya, Chunxia Zhai, Haijun Zhang, Zhiqiang Zhang, Bin Zhu, Sa'ed H Zyoud, and Samer H Zyoud.

Managing the estimation or publications process

Katrin Burkart, Simon I Hay, Ali H Mokdad, and Christopher J L Murray.
